# Scientific mobilization of keystone actors for biosphere stewardship

**DOI:** 10.1038/s41598-022-07023-8

**Published:** 2022-03-04

**Authors:** Henrik Österblom, Carl Folke, Juan Rocha, Jan Bebbington, Robert Blasiak, Jean-Baptiste Jouffray, Elizabeth R. Selig, Colette C. C. Wabnitz, Frida Bengtsson, Beatrice Crona, Radhika Gupta, Patrik J. G. Henriksson, Karolin A. Johansson, Andrew Merrie, Shinnosuke Nakayama, Guillermo Ortuño Crespo, Johan Rockström, Lisen Schultz, Madlen Sobkowiak, Peter Søgaard Jørgensen, Jessica Spijkers, Max Troell, Patricia Villarrubia-Gómez, Jane Lubchenco

**Affiliations:** 1 Stockholm Resilience Centre, Stockholm University, 106 91 Stockholm, Sweden; 2 South American Institute for Resilience and Sustainability Studies, Maldonado, Uruguay; 3 Graduate School of Agricultural and Life Sciences, The University of Tokyo, 1-1-1 Yayoi, Bunkyo-ku, Tokyo, 113-8657 Japan; 4 The Beijer Institute for Ecological Economics, Royal Swedish Academy of Science, 104 05 Stockholm, Sweden; 5 The Global Economic Dynamics and the Biosphere Academy Program, Royal Swedish Academy of Science, 104 05 Stockholm, Sweden; 6 Future Earth, Swedish Royal Academy of Sciences, Box 50005, 104 05 Stockholm, Sweden; 7 Pentland Centre for Sustainability, University of Lancaster, Bailrigg, LA1 4YW UK; 8 Stanford Centre for Ocean Solutions, Stanford University, 473 Via Ortega, Stanford, CA 94305 USA; 9 Institute for the Oceans and Fisheries, The University of British Columbia, 2202 Main Mall, Vancouver, BC V6T1Z4 Canada; 10 WorldFish, Jalan Batu Maung, Penang, Malaysia; 11 Potsdam Institute for Climate Impact Research, Telegraphenberg A31, 14473 Potsdam, Germany; 12 University of Birmingham, Edgbaston, Birmingham, B15 2TT UK; 13 Department of Integrative Biology, Oregon State University, Corvallis, OR 97331 USA

**Keywords:** Sustainability, Ocean sciences, Psychology and behaviour

## Abstract

The biosphere crisis requires changes to existing business practices. We ask how corporations can become sustainability leaders, when constrained by multiple barriers to collaboration for biosphere stewardship. We describe how scientists motivated, inspired and engaged with ten of the world’s largest seafood companies, in a collaborative process aimed to enable science-based and systemic transformations (2015–2021). CEOs faced multiple industry crises in 2015 that incentivized novel approaches. New scientific insights, an invitation to collaborate, and a bold vision of transformative change towards ocean stewardship, created new opportunities and direction. Co-creation of solutions resulted in new knowledge and trust, a joint agenda for action, new capacities, international recognition, formalization of an organization, increased policy influence, time-bound goals, and convergence of corporate change. Independently funded scientists helped remove barriers to cooperation, provided means for reflection, and guided corporate strategies and actions toward ocean stewardship. By 2021, multiple individuals exercised leadership and the initiative had transitioned from preliminary and uncomfortable conversations, to a dynamic, operational organization, with capacity to perform global leadership in the seafood industry. Mobilizing transformational agency through learning, collaboration, and innovation represents a cultural evolution with potential to redirect and accelerate corporate action, to the benefit of business, people and the planet.

## Introduction

Scientists interested in sustainability have devoted substantial efforts to study cooperation within local communities^1,2^, between national governments^3,4^ and among private corporations^5,6^. Studies on the role of scientists in such processes tend to focus on their ability to co-produce knowledge and action with local stakeholders^7,8^, transfer knowledge to government agencies^9,10^, or influence policy and politics^11^. Scientific cooperation with private corporations for sustainability is rarely a focus of study^12–14^, despite a widespread recognition that government action alone will not be sufficient to effectively address sustainability challenges—corporations need to support and accelerate change^15,16^. If that is the case, how can such engagement be achieved, and is there a role for scientists to support cooperative processes between private actors? We investigate whether transnational corporations associated with the ocean economy can transition from a non-cooperative to a cooperative state, and actively engage with their peers in collective action for ocean stewardship [defined as *an adaptive and learning based, collaborative process, of responsibility and ethics, aimed to shepherd and safeguard the resilience and sustainability of ocean ecosystems for human well-being*^17^].

In 2015, co-authors of this study identified 13 seafood companies with disproportionate influence (Supplementary Table S1) and termed them “keystone actors”^18^, as they dominate revenues and production volumes, control major segments of seafood production (in wild capture fisheries, aquaculture and feeds), connect ecosystems globally through subsidiaries, and influence governance processes and institutions. Given the combined size, influence and power of these 13 companies (in a sample of 160 corporations), we hypothesised that collective leadership in sustainability from such actors could generate cascading effects and norm changes in strategies and practice throughout the global seafood industry^18^. Systemic change would first require consistent and credible leadership by keystone actors. To mobilize such leadership, and create the conditions for future cascading effects, we approached these companies with an offer to organize a global keystone dialogue^17^. Eight companies initially responded positively to this invitation. The Seafood Business for Ocean Stewardship (SeaBOS) initiative emerged after the first keystone dialogue (2016), developed from subsequent interactions, became a formal co-operative organisation in 2019 and now engages ten of the world’s largest seafood companies^17^. Combined, they account for more than 100,000 employees, 600 subsidiaries in 95 countries, and 10% of global fish catches. The purpose of SeaBOS is to *lead a global transformation towards sustainable seafood production and a healthy ocean*.

Here we investigate how scientists can inspire, initiate, guide and support a pre-competitive business coalition striving to mobilize action for a sustainable future. We describe the evolutionary process of cooperation, the efforts required, how the nature of cooperation changed over time, and the associated incentives, enabling conditions, benefits, costs and risks. While previous studies have described the initial stages of SeaBOS^12,17^, the goal of this study is to illuminate the process of learning and cooperation over time, and in particular how scientists can facilitate strategic and long-term change within corporations. Given the gap in the sustainability science literature on processes of engagement with powerful actors^13^, and general scepticism about corporations in relation to sustainability^19,20^, we provide a comprehensive account of the entire process, from tentative conversations to tangible results. We reflect on the inherent barriers and limitations of such engagement, and show how cooperation between scientists and SeaBOS members has shifted priorities and activities of corporations, with a focus on science-based action for ocean stewardship. The study aims, therefore, to provide insights for scientists interested in co-developing and learning from processes of change with corporations.

## Results

Collaborative efforts to finalize and publish a paper on “keystone actors in marine ecosystems” (2014–2015) sparked an internal conversation among scientists about the opportunities to mobilise such corporate actors for sustainability. The rationale for initiating the keystone actor analysis in the first place, and subsequently engage in the process described here, was a continuous frustration about the slow progress towards ocean sustainability and excitement about the potential to engage large-scale producers in positive change. It was further enabled by an offer from the Soneva Foundation and Forum for the Future to host and facilitate a global dialogue, and the expressed intent from HRH Crown Princess Victoria of Sweden, who had just been appointed United Nations Sustainable Development Goals (UN SDGs) advocate, to engage in such a dialogue as Patron.

**Figure 1:**
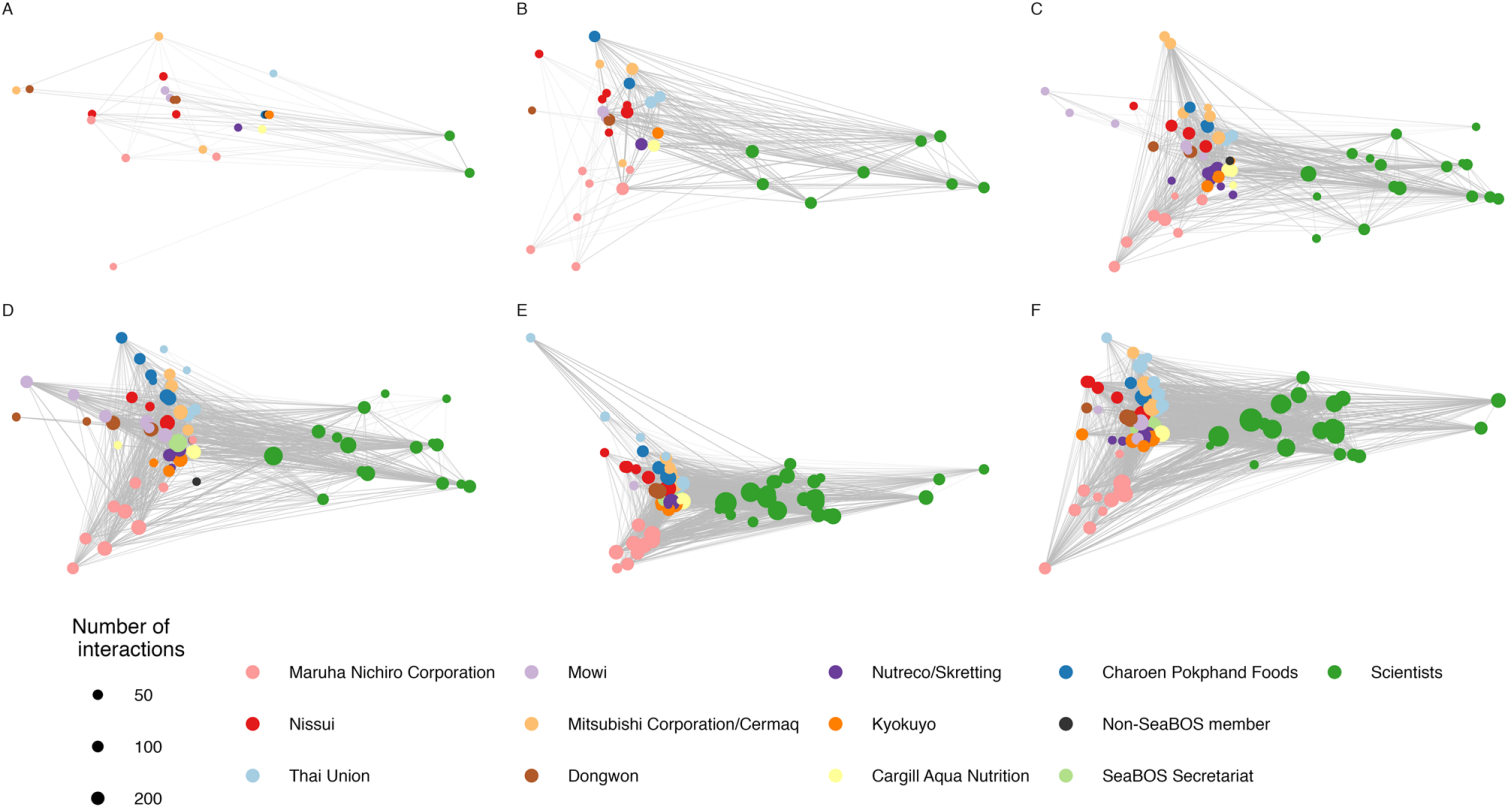
Evolution of connectivity between scientists and SeaBOS companies. **(A)** Initial regional connectivity, took place during Phase I, when scientists engaged multiple industry actors in individual or regional dialogues in either Japan, Norway or South Korea. **(B)** Phase II included two keystone dialogues, and resulted in the establishment of a global science-business network. **(C)** The formation of task forces during Phase III involved strengthened and diversified interactions between multiple scientists and company representatives. **(D)** Phase IV included a new coordinating function (a formal SeaBOS secretariat), and a set of time bound goals were defined during Phase V **(E)**. SeaBOS produced tangible results and can be described as having become fully functional during Phase VI **(F)**. Each node represents one individual and the size of the node corresponds to the sum of all interactions for each time period. The color of the node corresponds to the type of actor engaged in the networks.

During subsequent engagement (2015–2021), we documented 558 interactions between 135 industry representatives (from 13 companies headquartered in 8 countries), and 28 scientists (from 9 institutions in 6 countries), see Fig. 1. During this process, scientists and business representatives exchanged and translated knowledge, engaged diverse human capacity and external organisations, developed time-bound goals for action and produced tangible results. These interactions and their associated effects can be divided into six distinct phases, identified as the result of an in-depth engagement with and first-hand knowledge of, the SeaBOS initiative.

### Six emergent phases of stewardship

#### Phase I: Local meetings and individual incentives (March 2015–October 2016)

The collaborative process started with two scientists engaging in informal, primarily bilateral, meetings with CEOs and other individuals in keystone actor companies, to explain the scientific background and hypothesis—that a small number of powerful actors could enable transformative change^18^. These exploratory meetings were mediated by third-party brokers, including individuals with personal relationships to the CEOs or diplomatic responsibilities (e.g., Embassy of Sweden in Japan)^12,17^. Meetings in Norway were conducted in Norwegian/Swedish, whereas all other dialogues during this phase were conducted in English, with some Japanese translation. These initial meetings were characterised by a mutual interest, but exchanges were reserved and relied on indirect trust between participants. CEOs expressed their individual, strategic priorities and incentives for cooperation, and most of them appeared proud to have been associated with a company identified as a keystone actor. Several CEOs acknowledged that power and influence were also associated with responsibilities. Eight companies indicated their openness to further interaction and agreed to participate in a first global dialogue. Other identified keystone actor companies found limited, or no benefits from continued interaction (Table 1). Constructive meetings resulted in new networks and increased connectivity, and helped build knowledge and an understanding of potential gains and risks from collaboration^17^. They stimulated collaborative learning between science and business, and between companies in individual countries, but did not connect across geographies—since they primarily involved companies in either Japan, Norway, or South Korea^17^ (Fig. 1).

**Table 1:** Representative quotes from the described six phases.

	Quote	Comment
Phase I	“*I don’t have time for any [profanity] NGOs”*	Example quote from a company representative (not a SeaBOS member) representing a misunderstanding when scientists were not introduced by an “ambassador”
Phase II	“*I knew that I had to engage in sustainable seafood production, but I had never realised that I needed to be a leader in ocean stewardship”*	Comment from a CEO illustrating how the first keystone dialogue provided a broader perspective
	“*This is the first time that science and business work together, the first time that fisheries and aquaculture work together, and the first time that companies from all three major markets* [i.e. Europe, Japan, North America] *work together”*	The way the CEOs at the first keystone dialogue described the unique nature of SeaBOS
Phase III	*“you [scientists] need to remain as an engine and locomotive. I don’t think we can continue if you sit back and just stay as a research organisation*”	Comment by a CEO when scientists remarked that they did not have the capacity to continue coordinating the initiative
Phase IV	*“Anyone who attended the panel of the Seafood Business for Ocean Stewardship (SeaBOS) [….] might just have heard the most important development in the entire seafood industry”*	A description of SeaBOS, and in particular their partnership with GDST, by a leading seafood industry journal
Phase V	“*A frog at the bottom of the well knows nothing about what is happening in the Great Ocean”*	A company representative citing a Japanese proverb to illustrate the way learning was accelerating as a result of SeaBOS
Phase VI	“*We need cooperation between companies and geographies as we are operating in our common ocean. We need politicians to make science-based regulations and their support to make bold decisions”*	Comment by a CEO advocating for more ambitious politicians and more forceful ocean policies

#### Phase II. Global network with a shared vision (November 2016–June 2017)

The first (November 2016) and second (May 2017) keystone dialogue (Table 2, Supplementary Data S1) were attended by representatives from eight and ten keystone actor companies respectively, primarily CEOs, a handful of scientists, ocean experts, and HRH Crown Princess Victoria of Sweden^17^. Her participation, along with engagement of the Swedish Deputy Prime Minister during the second dialogue, served as prestigious attractors and likely helped signal that the issues and scientists were relevant and legitimate. Different incentives motivated participation from companies and the meetings took special care to integrate the priorities identified during the first phase (see “Methods”), along with scientific priorities and the recently established UN SDGs. These dialogues enabled new connections (Fig. 1), communication and trust-building between actors that had previously not cooperated around shared ocean challenges (although individual companies had cooperated in species- or region-specific initiatives)^17^. Scientific syntheses of challenges and opportunities in the Anthropocene^21^ were presented at the dialogues and placed in the context of the ocean. The dialogues were explicitly stated to represent “experiments” (not in a formal laboratory sense, but in a way to reflect the uncertain and previously untried open nature of the engagement) with an aim to explore the keystone actor hypothesis (see “Methods”). The dialogues, conducted in English, facilitated knowledge sharing between CEOs, and resulted in the development of a shared vision for ocean stewardship (Table 1). The scientific presentations of the systemic challenges were underpinned by a series of scientific background briefs, synthesising substantial amounts of information displayed in a format that was easily accessible to CEOs. The scientific information, combined with insights from CEOs, generated an understanding of the scale of the challenges, and their intertwined and profound impacts on seafood production and ocean sustainability. The sentiment that emerged was that there was no option but to work together—this was perceived both as an opportunity and as a responsibility. The scientific foundation was recognized by CEOs as a central and unique feature of the initiative (Table 1).

**Table 2:** Summary of main events, outcomes and supporting mechanisms.

Event	Details	Outcomes	Supporting mechanisms
1st Keystone Dialogue* Nov 2016, the Maldives *Transformative risks and opportunities for the global seafood industry*	Host: Soneva Patron: HRH Chair: FFF Companies: 8 CEOs: 5 Operational staff: 3 Advisors: 4 Scientists: 4	A shared vision for ocean stewardship Establishment of SeaBOS and agreement of commitments 1st statement published: *A commitment to ocean stewardship*	A sense of urgency, crisis, and opportunities in the seafood industry Openness and trust building at core of meeting (location, format, host, facilitator) A vision framed in science, guided by company priorities, and aligned with UN SDGs Anti-trust statements
2nd Keystone Dialogue* May 2017, Sweden *Advancing the SeaBOS initiative*	Host: SRC/KVA Chair: SRC Companies: 10 CEOs: 6 Operational staff: 6 Advisors: 2 Scientists: 7	Identification of priorities Task force leadership defined 2nd statement published: *A pledge and a plea for ocean stewardship* Interim secretariat established and interim chairman elected	Short time span between first and second dialogue to maintain momentum One CEO volunteer as strategic leader, scientists host secretariat, for one year. Co-investment model defined Swedish Deputy PM welcome SeaBOS to UN Ocean meeting
1st Working Meeting May 2018, Netherlands	Host: Nutreco Chair: Nutreco Companies: 9 CEOs: 3 Operational staff: 11 Advisors: 0 Scientists: 13	Connection between task forces established and action agenda defined Identification of priorities for 3rd keystone dialogue	Detailed operational workplan with focus on learning, sharing experience and increase trust Internal progress report is “wake up call” CEOs stress need to engage with reducing antibiotic use, plastics, to deliver results, and “earn the right to speak”
3rd Keystone Dialogue* Sept 2018, Japan *From Commitments to action*	Host: MNC, NSK, KK, MC Chair: Nutreco Companies: 11 CEOs: 10 Operational staff: 16 Advisors: 1 Scientists: 2	1st formal chair appointed Article of association, annual fee and budget defined Partnership with GDST established 3rd statement published: *Our pledge for combatting IUU fishing related to Japan*	All CEOs present, Japanese translation CEO leadership on supply chain mapping Greater scientific understanding of Anthropocene challenges, corporate realities and how to advance implementation
2nd working meeting* May 2019, Norway	Host: Mowi Chair: Independent Companies: 10 CEOs: 3 Operational staff: 16 Advisors: 1 Scientists: 8	Development of Task Force activities Identification of priorities for 4th keystone dialogue Agreement to provide information on use of antibiotics Agreement to develop company-specific plastic strategies	Task Force leaders take ownership and argue for novel technologies, plastics, transformation Scientists present risk map and request access to data Legal investigation trigger sensitive conversations about performance and reputational risks Anti-trust lawyers present, encouraging greater openness within clear legal bounds
4th Keystone Dialogue Sept 2019, Phuket *Global connectivity: consolidating and accelerating change*	Hosts: TU, CPF Chair: SeaBOS chair Facilitator: SeaBOS MD Companies: 10 CEOs: 8 Operational staff: 19 Advisors: 2 Scientists: 3	Agreement to report using GRI standards by 2020 Agreement to support Global Compact Ocean Action Platform and join GGGI Agreement to host workshops on traceability and AMR (cancelled due to pandemic) Agreement to pilot scientific risk map with one company Task Force on Climate Resilience established Governance developed (compliance and budget)	Membership fees paid and companies have financial stake, signalling commitment CEO progress report established as normal Scientific analysis highlight climate change risks and opportunities Informal social pressure by CEOs help advance norms Japanese members more active Translation to Japanese and presence of lawyers established as normal
3rd working meeting* May 2020, A virtual meeting due to the COVID-19 pandemic	Chair: SeaBOS MD Companies: 10 CEOs: 2 Operational staff: 20 Advisors: 1 Scientists: 13	Agreement to define time-bound goals for addressing IUU fishing, modern slavery, antibiotics, plastic pollution and climate Agreement to engage with endangered species	Anti-trust introduction normalised Company staff provide active leadership in task forces, present ambitious goals to peers, scientists, and HRH, thereby increasing confidence and trust Agreement on approach for goals
5th Keystone Dialogue* The Oct 2020, virtual keystone dialogue *Resilience through Ocean Stewardship—Leadership, actions, and opportunities*	Chair: SeaBOS chair Facilitator: SeaBOS MD Companies: 10 CEOs: 9 Operational staff: 19 Advisors: 1 Scientists: 18	2nd formal chair appointed Agreement to engage more actively in task forces Roles and responsibilities of companies, the SeaBOS secretariat, and the science team, formally established Agreement on time-bound goals, including; Oct 2021: Have no IUU fishing or modern slavery in our own seafood operations Dec 2020: Announce a time plan for implementing a series of science-based measures that when combined, substantially reduce the risk of IUU fishery products or modern slavery in our supply chain Oct 2021: Agree on time-bound goals for minimising bycatch of endangered species Oct 2021: Develop a road map for identifying ways to significantly reduce/phase out prioritised antibiotics and develop a code of conduct for antibiotics use Oct 2021: Establish science-based goals and reporting approaches for reducing greenhouse gas emissions 2023 timelines established for supply chain aspects of impacts	Active facilitation by SeaBOS MD and pressure from scientist prior to meeting important for agreement on goals Sensitive conversations about antibiotics help progress topic Confidence to make near term, time bound goals that encompass supply chain Participation by respected external scientist help reinforce that SeaBOS is working with right issues, in progressive way
4th Working Meeting* May 2021, A virtual meeting due to the COVID-19 pandemic *Updates and opportunities*	Chair and facilitator: SeaBOS MD Companies: 10 CEOs: 8 Operational staff: 20 Advisors: 1 Scientists: 20	Update on progress towards consistent reporting on goals by CEO meeting	Substantial and diverse results presented by all task forces. A feeling that momentum is building Companies take pride in demonstrating progress on range of issues Scientific reminder of stewardship vision
6th Keystone Dialogue* October 2021 virtual meeting *Delivering transformation for ocean stewardship*	Chair: SeaBOS chair Companies: 10 CEOs:10 Operational staff: 19 Advisors: 2 Scientists: 18	Reporting on progress towards agreed goals Agreement on strategy for reducing impacts on endangered species, roadmap for reducing use of antibiotics in aquaculture, raise ambition in relation to climate goals, and increase focus on communication and working with governments	CEOs communicate benefits of sustainability and task force leaders take pride in progress Failure to reach jointly agreed climate goals stimulate candid and trust building conversations Allegations of IUU fishing generate CEO statement about improving and learning from crises

For detailed agendas, scientific background and results, see Supplementary Data S1.

*FFF* forum for the future, *SRC* Stockholm Resilience Centre, *KVA* Royal Swedish Academy of Science, *MNC* Maruha Nichiro Corporation, *NSK* Nissui, *KK* Kyokuyo, *MC* Mitsubishi Corporation, *TU* Thai Union, *CPF* Charoen Phokphand Food, *PM* Prime Minister, *MD* Managing Director, *GDST* Global Dialogue on Seafood Traceability, *GRI* Global Reporting Initiative, *GGGI* Global Ghost Gear Initiative, *AMR* Antimicrobial resistance, *IUU* Illegal, Unreported and Unregulated. *HRH Crown Princess Victoria of Sweden present.

The first dialogue resulted in the formulation of an intent to engage in SeaBOS, with an agreed set of ten commitments as a foundation for joint, pre-competitive activities (Tables 2 and 3). These commitments were based on the scientific background briefs and associated discussions during the meeting. They were drafted by the science team, and revised and approved by the CEOs. The second dialogue resulted in prioritization among these commitments (Table 3), the appointment of the CEO of *Nutreco* as interim chairman of SeaBOS, and the science team volunteering as an interim SeaBOS secretariat (Table 2). These outcomes were communicated during the first UN Ocean conference in New York (June 2017), where SeaBOS was met with both excitement and scepticism. The first steps towards global cooperation had been taken, but SeaBOS was not functional—it did not yet have the capacity to mobilize action towards the stated vision of transformative change and the associated commitments.

**Table 3:** Commitments and action towards ocean stewardship.

Commitment at Soneva dialogue 2016	Companies that have	2016	2017	2018	2019	2020	2021
1. Improve transparency and traceability in our own operations, and work together to share information and best practice, building on existing industry partnerships and collaborations	Assessed materiality	7	7	8	9	10	10
	Reported with GRI	6	6	7	8	10	10
	Disclosed production volumes (ODP)	4 (1)	4 (2)	6 (2)	7 (2)	7 (2)	8 (3)
	Completed internal traceability assessment**						9
	Used GDST						4
2. Engage in concerted efforts to help reduce IUU fishing and seek to ensure that IUU products and endangered species are not present in our supply chains	Compliance policy	9	9	9	9	10	10
	Assessed risks with scientists**					1	4
	Time-bound IUU goal*					10	10
	Time-bound endangered species goal*						10
3. Engage in science-based efforts to improve fisheries and aquaculture management and productivity, through collaboration with industry, regulators and civil society	New formal partnerships (Supplementary Table S3)			1	2	1	1
	New policy statements (Details in Supplementary Table S3)		1	1	1	2	3
4. Engage in concerted efforts to eliminate any form of modern slavery including forced, bonded and child labour in our supply chains	Time-bound goal for reducing labor abuse*					10	10
5. Work towards reducing the use of antibiotics in aquaculture	Shared sensitive data with scientists**					4	6
	Road-map for reducing antibiotics*						10
6. Reduce the use of plastics in seafood operations, and encourage global efforts to reduce plastic pollution	Plastic inventory						10
	Strategy for reducing plastics*					10	10
	Time-bound plastic target				1	4	5
7. Reduce our own greenhouse gas emissions	Time-bound climate goal (SBTi goal)	3	4	7	8 (2)	9 (2)	9 (4)
8. Secure new growth in aquaculture, by deploying best practices in preventive health management, including improved regulatory regimes		Not yet a priority					
9. Collaborate and invest in the development and deployment of emerging approaches and technologies for sustainable fisheries and aquaculture		Not yet a priority					
10. Support novel initiatives and innovations for ocean stewardship		Not yet a priority					

*A common SeaBOS goal or activity, **information currently not available in the public record. Commitment 1–4 were identified as priorities by CEOs in 2017, 5–6 were added in 2018, and 7 in 2019. *ODP* Ocean Disclosure Project, *SBTi* Science Based Targets initiative

#### Phase III. Mobilising diverse capacities (August 2017–September 2018)

An extended group of scientists, the SeaBOS interim chairman, and other company representatives (Fig. 1) co-developed an action plan, and established and engaged in four task forces aimed at translating the identified priorities of the CEO commitments into operational activities, while also developing the formal governance of SeaBOS (see Task Forces I–IV, Supplementary Table S2). The interim SeaBOS secretariat consisted of scientists, who led and facilitated interactions and enhanced communication, thereby ensuring that the costs of collaboration for companies were limited to their individual time commitments and travel. The interim chairman of SeaBOS provided important leadership, acted as main point of contact between science and business (Fig. 1) and helped co-develop strategic priorities of the initiative. Collaboration within task forces also integrated substantial advice from multiple informal and formal partners (Supplementary Table S3). This operationally focused work strengthened and stabilized the relationships, while also generating trust and informal norms of cooperation. Starting from different contexts, cultures and situations, the CEOs ensured that companies mobilised internally to develop the adequate capacity to produce results in line with their commitments. Such activities included revisions to codes of conduct, employment of additional staff, internal reorganisation of units and processes to facilitate an integration of sustainability across departments, as well as updated risk assessments, and new inventories of raw materials procured (Supplementary Data S2). These aspects emerged reflexively from a mix of CEO leadership, company strategic orientation and operational capacity, not from a single source.

All operational individuals and three CEOs met for the first time in a global working meeting in May 2018, hosted by *Nutreco* at their headquarter in Amersfoort, the Netherlands (Table 2). Task forces were able to connect with and learn from each other, and scientists provided definitions of key terms, to ensure a common starting point for action. Improved transparency and traceability were identified as important areas to focus on, as a foundation for all commitments. A scientific assessment of reporting practices concluded that each company should first conduct a materiality assessment (a process to define environmental, social and governance issues of relevance for their business), and then report in accordance with the Global Reporting Initiative (GRI) standard as a primary means to improve transparency. Companies were also encouraged to make voluntary disclosures through the Ocean Disclosure Project (ODP) and to engage with the Global Dialogue on Seafood Traceability (GDST) to advance traceability (Table 3). The meeting included a presentation of *Nissui*’s first ever mapping of their entire production portfolio in relation to sustainability (which was subsequently published and replicated by the other two Japanese members, see Supplementary Table S2). The formal governance of SeaBOS started to develop, and steps were taken to recruit a managing director, although the continued engagement by scientists was identified as instrumental (Table 1). Members agreed that SeaBOS companies needed to “earn the right to speak”—they would only be regarded as credible if they had addressed their own challenges (Table 2).

The third keystone dialogue was hosted by *Maruha Nichiro Corporation, Nissui, Kyokuyo* and *Mitsubishi Corporation* in Karuizawa, Japan (September 2018). The meeting was facilitated by the interim chairman and included professional Japanese translation. HRH Crown Princess Victoria of Sweden was present throughout this meeting, where She engaged, encouraged and challenged the CEOs. This was the first time that all CEOs participated, together with company staff. Governance and funding of SeaBOS were defined, and it was agreed that all companies would pay an annual contribution that would be used to employ a managing director and a small secretariat, independent from the science team. The first SeaBOS chairman (The CEO of *Maruha Nichiro Corporation*) was elected and companies agreed to work with the GDST to advance traceability (Table 2). An internal scientific assessment of members’ sustainability performance and outcomes of a survey on risks of exposure to Illegal, Unreported and Unregulated (IUU) fishing and labour abuse provided means for reflection. Draft voluntary actions for addressing these issues, along with Key Performance Indicators (KPIs), were discussed.

#### Phase IV. Global recognition established (October 2018–December 2019)

The frequency of interactions increased and additional scientists were engaged to develop a set of maps identifying where risks of IUU fishing and labour abuse were greatest to help guide company priorities for action (Fig. 1). These issues had been identified by CEOs as particularly salient and risk maps had been requested by companies in 2017 that would increasingly represent an important tool for their due diligence processes (Table 3). An onboard monitoring program was piloted by *Nutreco* to help address challenges associated with IUU fishing and labour abuse through electronic monitoring, species identification, and facial recognition software. These activities were presented at a second global working meeting, hosted by *Mowi* in Bergen, Norway (May 2019), where discussions addressed the challenges of reducing antibiotics use and sharing of company data. This meeting also covered sensitive topics, including how companies would act if a member would be identified as guilty of breaking laws (Table 2). Such candid conversations helped build trust, stabilise relationships, and reinforced the commitment to a joint vision.

SeaBOS became a legal entity and was established as a Swedish fundraising foundation in June 2019. The process of setting up this organisation had been led by the SeaBOS interim chairman, with external legal support. SeaBOS members started paying an annual contribution to the foundation, to employ a managing director. A number of coordinating responsibilities were subsequently transferred from the interim secretariat (the scientists), to this SeaBOS secretariat (the managing director). Both scientists and SeaBOS company representatives served as executive board members of the SeaBOS fundraising foundation^12^.

Global recognition started to accelerate, in part through articles in the Financial Times^22^ and a leading seafood industry journal (Table 1)^23^. At the fourth keystone dialogue, hosted by *Thai Union* and *CP Foods* in Phuket (September 2019), companies agreed to accelerate action, adopt a joint reporting approach (using the GRI standard by October 2020), and establish a new task force on climate resilience, thereby further expanding the scope beyond existing task forces (Supplementary Table S2). New partnerships were established with the Global Ghost Gear Initiative (GGGI) to reduce plastic pollution from fishing and aquaculture activities, and with the United Nations Global Compact Action Platform for Sustainable Ocean Business (renamed the “Ocean Stewardship Coalition” in 2021). Formal governance mechanisms were refined, such as decision making, compliance, conflict resolution, member exclusion, and logotype. The roles and responsibilities of scientists, the companies and the SeaBOS secretariat, respectively, were clarified and explicitly defined (Table 2).

However, companies were not yet able to agree on a set of KPIs, perceived as too restrictive and inflexible, and encouraging a “one-size fits all” approach that companies were not ready for. Internal reports clearly illustrated that all companies were committed to change, however, and were starting to show progress (Supplementary Data S2). This meeting h﻿e﻿﻿lped establish Japanese translations at dialogues as the new norm, which likely contributed to the Japanese members being more confident and engaged. We also observed that CEOs were becoming more invested in their joint agenda. A few leading CEOs were exercising informal social pressure in plenary conversations to ensure that all companies had the same level of ambition, and to influence companies that were identified as lagging behind (Table 2). Such activities were likely a result of them now being financially invested in SeaBOS and under growing scrutiny—with global recognition and the risk that laggards could negatively influence the perception and seriousness of SeaBOS and its agenda.

#### Phase V. Agreement on time-bound goals (January–December 2020)

Numerous science-business interactions (Fig. 1) stimulated further collaborative learning between companies, as well as relationship building with two new CEOs. These meetings included conversations in task forces to agree on a set of ambitious but realistic goals, and also involved external stakeholders that could support the process of making such goals achievable. A third working meeting took place virtually (May 2020) and resulted in identification of potential time-bound goals (rather than KPIs) for eliminating IUU fishing and labour abuse; an ambition to set science-based emissions reduction targets for greenhouse gases; a commitment to develop a strategy to limit impacts on endangered species, a pledge to assess and reduce plastics footprints; and the development of strategies to reduce the use of antibiotics in aquaculture (Table 2). This expansion of focus indicated a desire to address a wider range of ocean stewardship issues. Company representatives described how the new scientific knowledge they now had received helped them learn faster than prior to SeaBOS (Table 1)*.* The increase in the number of issues addressed also represented a challenge, since companies prioritized differently, and some issues were simply more relevant for some companies than others.

Member companies became increasingly involved in ocean policy developments, including in the High Level Panel for a Sustainable Ocean Economy (HLP)^24^ and in a coalition focusing on addressing IUU fishing. Joint public advocacy by SeaBOS companies focused on science-based fishing quotas and government support for boat crews and workers (Supplementary Table S2). SeaBOS had become a platform for increased global policy influence and collaborative learning, but also needed to demonstrate tangible results. Agreeing on time-bound goals was instrumental to illustrate credible progress and demonstrate accountability. The fifth (virtual) keystone dialogue (October 2020) was preceded by substantial interactions to reach consensus on a set of acceptable goals. The SeaBOS managing director engaged in multiple conversations with member companies while the scientists expressed what they found to be non-negotiable to companies that were hesitant to agree on ambitious goals. This was the first time that the tone of scientists changed from being mostly encouraging to also using their established influence as leverage to require a certain level of ambition. This process also clarified important cultural differences between companies in relation to goal setting, with Japanese members hesitant to agree on targets that they were not convinced that they could reach. These companies also had more limited experiences with integrating sustainability in strategies and operations, where exposed to less consumer pressure for sustainability and had more complex supply chains than their peers. At the meeting itself, CEOs agreed and committed to time-bound goals for eliminating IUU fishing and labour abuse, setting science-based goals for reducing greenhouse gas emissions, and plans to develop a strategy for reducing impacts on endangered species, address antibiotic use and decrease plastic use (Table 2). These goals were communicated in December 2020, along with operational support that would make them a reality (a set of voluntary procurement actions and a tool kit for action, see Supplementary Data S1). The goals represented an important signal that companies were now ready to be held individually accountable, with sufficient confidence in SeaBOS and that other members would also deliver results. Although the level of ambition of the agreed goals were lower than what had been discussed at the working meeting in 2020, they were demanding, realistic and acceptable to all members.

#### Phase VI. Convergence of action for stewardship (January–October 2021)

In February 2021, SeaBOS released a call for private and public action to address IUU fishing, together with several seafood multi-stakeholder platforms^25^. A working meeting in May indicated that all companies had initiated changes in their strategies and operations. They had also initiated work on a general seafood industry guide for setting science-based targets for reducing CO_2_ emissions. Several SeaBOS CEOs volunteered to engage as strategic sponsors for individual commitments, which effectively meant that they could help advance the issues that they were most committed to as individuals and business leaders. The result was that the CEOs started to work more actively with individual task force leaders, and with each other.

By the 6th (virtual) keystone dialogue (October 2021), the CEOs engaging as strategic sponsors took on a more active leadership role than in previous meetings. This meant that rather than having a meeting dominated by scientists, or an individual chair or facilitator, it was now a more active conversation led and advanced by the CEOs themselves. By this meeting, all companies had adopted the GRI standard. Nine out of ten members had set a climate goal, and all of them agreed on a shared strategy for reducing negative impacts on endangered species (Table 3). They also agreed on a joint road map for reducing antibiotics use (Supplementary Data S1), although work on antibiotics remained a challenge. Members had advanced transparency and traceability (Table 3) and now all had operational (and more sophisticated) policies that governed their sourcing of raw materials (from own operations and from their suppliers), with codes of conduct for biodiversity, human rights, vessel management and worker recruitment. Companies had used the co-designed and publicly available science-based voluntary procurement measures and the tool kit for action to address this goal. A number of companies were also working with scientists to identify geographical areas, fishing gears, ports or flags that were particularly risky in relation to IUU fishing and labour issues, using the scientific risk maps (Table 3). CEOs stated that these measures (combined with their own due diligence approach) had reduced the risks of such issues in their own operations, and that the time had now come to focus attention on the challenging aspects of ensuring the reduction of such risks throughout their supply chains. Although more information was needed to credibly illustrate how companies had operationally changed their practices and reached their goals, the CEOs expressed that they had delivered initial results on their commitments and argued for increased communication and work with governments to enable transformative change (Table 1). Such communication would include publishing a first SeaBOS progress report for launch during the 2022 UN Ocean Conference. Despite not having reached all the time-bound goals from 2020, the combined activities illustrated a convergence of priorities and associated actions (Table 3), suggesting that SeaBOS had become operational.

### Characteristics of the six phases

The six phases varied substantially in the number and type of individuals involved, meeting frequency, and whether or not interactions were mostly in person or virtual, carbon emissions, and available funding for the scientists (Fig. 3). There were also common denominators across all phases. Prior to every keystone dialogue or working meeting, we described them internally (in the science team) as representing a critical turning point and a “make or break” moment. After every meeting, we reflected that it had exceeded most expectations. This feeling of balancing on the edge of our capacity and between failing and succeeding, likely suggests that we were pushing ourselves, and the companies, to the extent thought possible at the time. Another consistent feature of all phases was the active engagement by HRH Crown Princess Victoria. Her opening remarks at the Embassy of Sweden in Japan during an informal meeting between the first and second dialogue, at the UN Ocean Conference in 2017, and during keystone dialogues or working meetings, helped set the tone of each meeting and the intended level of ambition. Her closing statement in turn, signalled to what extent the companies had produced results in line with these opening statements, while also highlighting future challenges.

One or two key individuals in each company were particularly engaged, with the most active typically participating in twice as many meetings as the second ranked (constituting a CEO in eight of ten instances). The most active participant from all companies featured in meetings throughout Phases III and VI. This pattern of long-term and consistent engagement by individuals was paralleled by an increasing number of active representatives from companies. Supplementary Fig. S1 illustrate the increasing number of person-meetings, by organisation over time, and highlight the coordinating role played by scientists and increasingly also by the SeaBOS secretariat.

**Figure 2:**
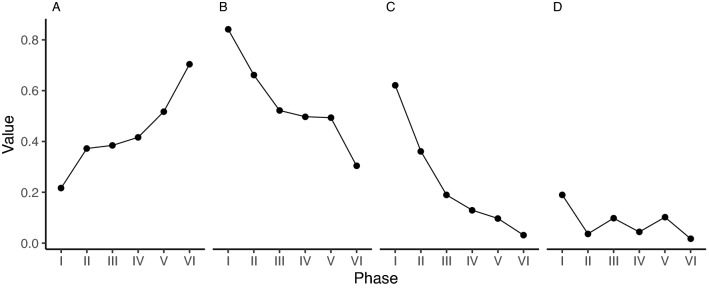
Properties of the network during the six phases of emergence. Density **(A)** shows the proportion of connections realized over the maximum number of connections possible. Local and global centrality metrics of all science-business interactions were calculated as normalized mean degree **(B)** and betweenness **(C)** centrality, respectively. The Bonacich power centralization (see “Methods”) measures how power is distributed across the network **(D)**.

Initially, the network had almost a bipartite structure, when scientists had many connections to companies, but company individuals were not connected unless they were from the same corporation (Fig. 1). This initial reliance on a small number of coordinating scientists, working closely with the interim chairman during Phase III, developed in to an increasingly diverse network (Fig. 2), where the SeaBOS secretariat and primarily its managing director occupied a central position from Phase IV (Fig. 1). Degree centrality decreased over time (Fig. 2), illustrating how cooperation became less dependent on one particular set of actors. This local network feature is consistent with measures of betweenness centrality, illustrating how the global importance of each individual was decreasing (Fig. 2). The average power of individuals was relatively stable over time (Fig. 2), suggesting that power was evenly distributed across the network.

**Figure 3:**
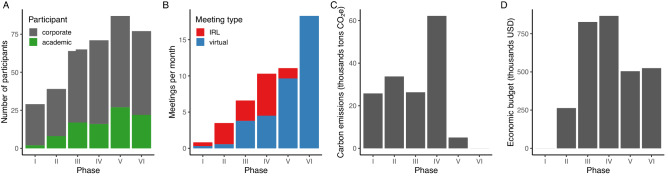
Science-business engagement, meeting frequency and costs. **(A)** The number of participants from companies and from science. **(B)** The frequency of virtual and in person (IRL) meetings. **(C)** The science-team travel-related carbon emissions (CO_2_e). **(D)** Dedicated science budget (USD).

**Figure 4:**
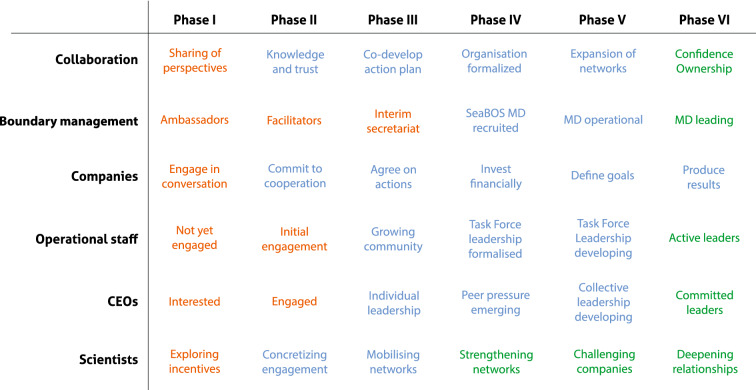
Evolutionary components of science-business cooperation. Colour coding of different components and actors engaged in collaboration, representing a qualitative assessment of the evolution of cooperation from an early and unstable phase, to operational, stabilised and functional cooperation. Colours refer to: insufficient, short term and vulnerable (orange); positive developments and long-term commitments, but not stable (blue); Adequate and resilient (green). *MD* managing director.

The number of individuals engaged increased substantially during Phase III, when task forces and operational activities were initiated (Fig. 3). Interactions during Phase I-II were limited and mostly conducted in person. Phase IV included a large number of long-distance trips, and high levels of CO_2_ emissions (Fig. 3). Travel during Phases V–VI was restricted due to COVID-19 and meetings were mostly virtual. The initiative has consistently relied on substantial and frequent engagement by multiple scientists and company representatives (Fig. 3), underlining the continuous dependence on science for progress. The scientific engagement has received dedicated and independent funding through a series of philanthropy grants awarded between 2016 and 2019. Companies acted as hosts for two working meetings and two keystone dialogues, and their annual contribution funded the SeaBOS secretariat and part of meeting logistics (see “Methods”). This long-term development has resulted in a collaborative, and increasingly institutionalised culture and shared sense of purpose (Fig. 4). Cooperation now transcends companies, geographies and scientific institutions and has likely made SeaBOS less vulnerable to changes in engagement of specific individuals and shifts of CEOs.

## Discussion

Transnational corporations are often described as problematic from the perspective of sustainability^19,20^, as a consequence of their primary profit motive, power to stifle innovation and sustainability practices, capacity to set barriers to entry for smaller actors, or impose unfair conditions for suppliers, or lobby against regulations^16^. They have been described as able to frame sustainability narratives in ways that advance the sustainability of their own operations, rather than that of the planet^19^. Our approach has been to explore whether keystone actor companies, if guided by a scientific vision of ocean stewardship and provided with substantial new knowledge and capacity, can operate as leaders of biosphere stewardship^6,12,16^. To have a scientific analysis as basis for interaction, and scientists that co-developed priorities and strategies, was in our assessment, instrumental for company engagement. This was often described by them as unique and the most valuable aspect of SeaBOS. Our results suggest that corporations can work with each other and with science, and that such engagement results in collaborative learning and converging corporate change. To our knowledge, this is the first time that an empirical scientific analysis of power (as defined by the concept of keystone actors) in an industry has directly resulted in a carefully curated process of science-based change with an ambition to facilitate transformative change.

Corporate cooperation is unlikely to occur without clear incentives^26^, since immediate issues are likely to take precedence over more costly, and longer-term systemic stewardship engagement^16^. Similar conditions apply to scientists, incentivised to focus on academic publications, grant writing and teaching. The urgency of the ocean challenges, the potential to try something novel and conceptually promising, although with uncertain effects and high risks, stimulated both corporate actors and scientists to engage in this process. Maintaining cooperation requires attention to formal and informal design principles^1,5,27^, including ensuring that decision-making processes are legitimate, rules and expectations adequate, and that monitoring, sanctioning and conflict resolution mechanisms are appropriate. The process of co-developing a set of formal and informal governance mechanisms resulted from the numerous interactions described here. These mechanisms are now largely in place, setting the stage for public reporting of progress, and subsequent monitoring and accountability.

### Scientists remove barriers to cooperation

Previous work has illustrated how scientists can operate as brokers between science and policy to achieve outcomes associated with sustainability^9,10,28^. This literature often assumes that there are cooperative organisations and institutions to work with and that challenges are primarily associated with knowledge exchange. This is not the case for the private sector, where corporations are competitors and there are few mechanisms for industry collaboration associated with the wider stewardship challenge. The literature on corporate-based voluntary environmental programs is substantial^5,29^, but such programs are primarily focused on specific challenges and few (if any) have to our knowledge been facilitated and enabled by university-based scientists. Many voluntary corporate sustainability initiatives have been initiated by environmental NGOs^30^ and this is also the case for several market-based seafood sustainability initiatives^31^. However many CEOs in our study expressed an initial unwillingness to work with NGOs, as they were perceived to have a pre-defined agenda and often narrow issue focus^17^. Scientists, instead, were regarded as trustworthy knowledge-providers and neutral conveners, with expertise to cover a wide array of issue areas.

The sustainability focus of keystone actor companies prior to 2016 was on immediate operational concerns rather than wider ocean stewardship challenges and opportunities^17,18^. The interactions (2015–2021) clarified the importance of a healthy ocean for long-term seafood production and illustrated the interdependencies of companies that operate in interconnected supply chains—they share reputational, biological, social and economic risks^32^.

Fragmented social networks, competition, limited vision, a lack of knowledge or expertise, existing legal barriers (including worries about breaching anti-trust regulations), prioritization of short-term profits and cost concerns, represent barriers to collaboration, and can be seen as explanations for limited cooperation between corporations on long-term sustainability challenges^2,16,33^. The costs of coordination can be high, especially when engaging a diversity of companies with different languages, structures and practices. Scientists can however help overcome such barriers, by presenting the scientific state of the art, facilitating local learning (to understand incentives of individual companies) and global connections (to clarify interdependencies and build trust). Multiple individuals and institutions played important boundary spanning (managing) functions during the course of the study, which facilitated communication, translation and mediation^14^. The interim chairman of SeaBOS (the CEO of *Nutreco*) co-developed an ambitious agenda and set the initial pace, whereas the first and second chairpersons helped build further legitimacy of the initiative. Corporate staff provided operational leadership, and generously shared their experiences, knowledge and networks with competitors (Fig. 4). The SeaBOS managing director was collaboratively governed by companies and scientists, which formed the board of the SeaBOS fundraising foundation. This dual accountability helped facilitate negotiations and develop trust between diverse interests, as has been observed in other organisations working at the boundary of knowledge and action^14^.

Ocean stewardship served as an attractive vision, and cooperation was inexpensive for individual companies at the onset, since academic institutions initially bore all financial and coordination costs. Scientists consequently served as “boundary spanners”, by connecting diverse companies in a social network^2^. Scientists also developed most of the background documents, facilitated connections to external organisations that supported the process of translating commitment to operational activities, co-developed the strategies for task forces, and produced other material and connections that helped advance the SeaBOS agenda. Refined SeaBOS governance, new funding mechanism, increased leadership by CEOs and operational staff, and the increasingly central role of the SeaBOS managing director in the network, generated support for these time-consuming coordination functions, similar to observations from other collaborative networks^2^. Continuous legal advice associated with anti-trust issues remains a developing and crucial component of SeaBOS, as cooperation between companies evolves.

The establishment of novel bonds is critical for enabling human cooperation^34^. The first bonds between actors in either Norway or Japan formed rapidly and were stabilized by a shared geographical identity—companies of the same nationality and experiencing similar operational contexts understood each other and the problems they were facing. Studies of cooperation illustrate that humans preferentially learn from individuals of the same ethnic group, where a set of common norms are shared^35^. In the Anthropocene, the sustainability challenges are interdependent and require a systems perspective with concerted action across sub-systems to be effective^6,32,36^. This requires social learning beyond the immediate community^2,37^. Consequently, scientists facilitated knowledge sharing and learning across cultural groups, thereby mobilising diverse capacities, skills and knowledge systems^38^ to collaboratively explore and test new solutions. Such connection of otherwise (largely) disconnected networks, stimulates information access and influence^39^. The stronger ties and dense network structure that subsequently developed facilitate collective action and effective collaboration^2,40^. Our findings are consistent with other studies of environmental governance, which emphasise that leadership is a time consuming, collaborative, and mutually supportive process, where networks are consciously expanded to enhance their transformative potential^8,41^. Now that SeaBOS has become functional, more efforts by companies can be directed towards focused and collaborative work to improve their own supply-chains and strengthen government regulations in multiple geographical locations, so that other companies also face legal frameworks more aligned with principles of ocean stewardship.

### Scientists as enablers of cultural evolution

Cultural evolution is a process whereby norms and practice spread through learning and mimicking^42,43^. Mechanisms for adopting new skills include mimicking individuals of high social status (prestige-biased transmission) or successful individuals (success-biased transmission)^35,37^. HRH and the CEOs have a high social status and their continuous engagement is a strong signal to other individuals among the companies to prioritize stewardship^44^. Tentative observations suggest that SeaBOS members tended to mimic the sustainability practice of their “more successful” peers (Table 3, Supplementary Data S2).

Cooperation requires trust, which implies that there is a mutual belief that expressed intentions are also reflected in attitudes and behaviour^28,45–47^. Our long-term, face-to-face interaction resulted in the emergence of collaborative norms of reciprocity and trust^2,45^, which facilitated a cultural evolution^37^ where new insights, norms, and practice benefitted all participants. Leadership in terms of vision, methods, specific knowledge and depth of operational expertise was distributed across the group. Different elements were important at different times, and there was no single transformational leader in the cohort. Goal setting has contributed to tangible results for SeaBOS members as a group, and similar approaches have been described as instrumental for the success of international governance regimes^4^. Whether these combined activities represent an integration of ocean stewardship as a core business strategy, and if they are sufficiently ambitious to be transformative, remain to be determined. Transformative change likely require stronger political leadership^16^, and clear incentives for companies to act as leaders, including financial instruments that reward biosphere stewardship^48^, new knowledge, and demand from markets, consumers and civil society^31,49^. Ocean stewardship is however increasingly prioritized by multiple political agendas, which may increase the prospects for transformative change^24^. The SeaBOS initiative is becoming a source of inspiration for other ocean-based sectors^50,51^ and the keystone actor idea is gaining traction in science^50,52,53^.

All SeaBOS members have made changes to their sustainability priorities and action since 2016, but they operate in complex environments and are subject to multiple influences, including global trends toward increased industry transparency and engagement in sustainability^31,54^. The first dialogue in 2016 was preceded by international media reports of labour abuse in wild capture fisheries, agreement on the UN SDGs, new investments by U.S. foundations in sustainable seafood (including in Japan), and preparations for the Tokyo Olympics. During the course of the process described here, ocean issues became more salient among politicians and the private sector, including as a result of the UN Ocean Conference, scientific analyses underpinning the HLP and the UN Global Compact, and new findings that helped mainstream the concept of ocean stewardship^50,51^. Other notable developments include the first Japanese fisheries policy reform in 70 years (2018), the first assessment of the Seafood Stewardship Index^55^ (2019), the COP 26 meeting on climate change in Glasgow and the UN Food Systems Summit (both in 2021). These developments influenced the perception of risks and opportunities for companies. Consequently, not all observed changes can be attributed to the SeaBOS initiative and it is impossible to identify single causes for corporate engagement or subsequent outcomes. Annual reports from SeaBOS members (Supplementary Data S2), however, suggest that many of the observed developments are a direct result of the support provided by the scientific team. This study did not evaluate the hypothesis that keystone actors are capable of generating cascading industry change, but it provides a baseline for evaluating such possible effects in the future.

### Scientific risks and unavoidable challenges

Scientific engagement with industry leaders may represent a substantial risk^12,17^, including potentially damaging scientific careers and reputation^14^ (particularly if such engagement results in little more than greenwashing). However, transdisciplinary science requires engaging in experimentation and acceptance of risks^56^. Scientists continue to be engaged because the process has resulted in new scientific knowledge and trajectories, new scientific insights and (what is perceived as) accelerating corporate action and an honest intent. It represents an opportunity to learn from company representatives and corporate perspectives, a chance to support transformative actions, and an exploration of the role that science can play in society^57^. Financial independence from companies (see “Methods”) and core funding from employing institutions for academic research enable unconstrained scientific work and an opportunity to publicly hold companies accountable. However, scientists have no formal powers to coerce companies to engage in any specific action: the relationship remains informal within an agreed set of principles (Supplementary Data S3–S4). The balance of power is maintained by companies respecting and appreciating the scientific advice and guidance obtained. Like many collaborative efforts, effectiveness relies on informal social norms, trust between individuals and a long-term commitment to strive towards a joint vision^58,59^.

Reluctance of companies to agree on shared goals, failures or challenges to reach them, or allegation of involvement in collusion or illegal activities during the course of this study, did not represent existential crises for the initiative or sensitive topics to avoid. Such critical events were instead used as opportunities to strengthen relationships and build further trust between participants. Although co-authors of this study strive to remain impartial in the interpretation of progress and have developed a corresponding methodology (“Methods”), independent assessments of the sustainability performance of the seafood industry^55^ represent important and complementary mechanisms for evaluating the performance of SeaBOS members and the extent to which they exercise leadership in comparison to their peers. Such assessments, along with public SeaBOS reporting are also likely to increase public pressure for accountability of SeaBOS members to their commitments.

Trust depends on continuous, face-to-face engagement and longevity of relationships. However, SeaBOS has had to navigate seven out of the ten members changing CEOs at least once since 2016, where existing CEOs consistently briefed their replacements on the salience of SeaBOS. The virtual meeting format during the COVID-19 pandemic has resulted in a marked absence of the informal conversations associated with earlier meetings. Other noteworthy challenges include motivating initial engagement of all CEOs, understanding the complex operations of the diverse companies involved, transferring coordinating responsibilities from science to companies^12^, obtaining sensitive data, agreeing on time bound goals, cultural clashes or misunderstandings, and the risk that companies will not be willing or able to move from incremental changes to transformative actions. Perhaps some of these challenges could be overcome by simply interacting more, but would likely also benefit from stronger social pressure^58,60^ and other external incentives^48^.

### Collective leadership for ocean stewardship

This case study describes co-production of knowledge in ways that differ from many examples of environmental governance—specifically since it aims to “broker power”, *cf*.^13^. Scientists are defining the problems, but solutions are co-designed with industry leaders. This work has been described as an experiment and an opportunity to collaboratively explore an evolving vision of stewardship^59^ and a joint capacity to enable systemic change^17^. The study represents an opportunity for participants to explore potential other values of business (in addition to profit) and science (in addition to publications). It has similarities with recent findings on how academics engage in environmental collective leadership^8^, including how leaders build trust and co-produce knowledge and action by using six different “dimensions” (inquire, connect, engage, strategize, empower, reflect)^8^. An increasingly ambitious approach taken by companies, growing engagement and enthusiasm by CEOs, and a distinct movement towards concrete results, have established a relationship characterized by empathy and mutual respect *cf*. ^8^. This is a marked difference from the initial meetings, characterized by limited trust and an uncomfortable uncertainty.

Transnational corporations are not typically known for enabling systemic and transformative change towards sustainability. There are however indications that such historical corporate logics and purpose are changing to also consider a longer-term perspective, including by engaging in *corporate biosphere stewardship*^16^, *regenerative capitalism*^61^ or *doughnut economics*^62^. The SeaBOS initiative has contributed to staking out a new vision, direction and opportunities for an industry in crisis. Our study describes the ability of scientists to remove barriers to cooperation, how private actors can contribute to shaping a new narrative for the ocean^63^, and start acting on it. This is not a quick fix, and credible industry leadership requires substantial work by diverse individuals and organisations. The keystone actor companies in SeaBOS engaged in, and embarked on an ambitious process with a vision striving towards ocean stewardship. Scientists reframed the ocean crisis as a stewardship opportunity, and continuously provided scientific insights as a bottom-line for action. Progress to date is the result of an emergent combined effort, building on expertise and energy of multiple individuals, who collectively constitute a new form of collaborative transformative agency. For participating scientists, it has resulted in a marked change of focus and direction—from observers of change and formulators of problems, to becoming active facilitators of, and partners in, transformational corporate change.

## Methods

Scientists (co-authors of this study) provided scientific guidance, process leadership, coordination and strategic advice during the development of the initiative. We collected information on industry “best practices”, identified and connected SeaBOS members and task forces to relevant seafood or ocean sustainability organisations to be able to build from and expand on existing efforts, methods and expertise (Supplementary Table S3), and gathered data about progress among individual SeaBOS member companies (see below). This material was communicated to participants from companies to strengthen their operational support, and their ability to engage in reflection and learning.

### Experimental design

The keystone dialogues were defined as carefully curated interactions between scientists and executives from keystone actor companies, based on science and aimed to stimulate collaborative learning^38^, co-production of knowledge^7^ and action for biosphere stewardship^16^. The aim of these dialogues and associated interactions was to investigate the hypothesis posed by Österblom et al.^18^ that keystone actors could potentially generate cascading social-ecological change for ocean stewardship. This required engagement at the strategic level, hence the priority was to first engage CEOs in a bold vision, rather than operational staff. We approached the CEOs of the identified keystone actor companies (Supplementary Table S1) but were unable to convince all of them to engage, despite multiple attempts. With ten companies accepting to participate, it soon became apparent that one annual CEO-level meeting was not enough to keep pace with the level of ambition of the initiative and deliver tangible results. An annual working meeting gathering scientists, operational staff and sometime CEOs, was therefore established systematically a few months before and in anticipation of the CEO keystone dialogue, after the second keystone dialogue.

The focus on *ocean stewardship*, rather than *sustainable seafood,* aimed to develop a sense of responsibility for the entire ocean, including its ecosystems, seascapes, and people, on which seafood business depends. Rather than individual companies, we engaged a group of corporations in collective action.

Keystone dialogues involved substantial scientific preparation and literature reviews, which was communicated to companies in scientific background briefs (see http://www.seabos.org). New scientific material, identified as important by either scientists or business representatives, was subsequently developed for advancing work in individual task forces (e.g., on ocean equity, an instrumental issue for ocean stewardship that was not addressed by the original SeaBOS commitments), see Supplementary Table S2. These findings were presented to business representatives as the basis for discussions and for the development of priorities and actions. Scientific presentations at keystone dialogues also included information about the wider global social-ecological challenges—of which the ocean is part—to illustrate the dynamic earth-system interactions and the need for stewardship. Knowledge exchange from business to science also critically informed the process as observations from, and experiences by, industry representatives contributed to developing new knowledge among scientists.

The initiative was novel for participating scientists and business representatives, and was not possible to plan in detail from the beginning, due to elements of unknowns, uncertainty and surprise. Instead, it developed through a process of learning by doing. The first keystone dialogue was extensively planned in collaboration with Forum for the Future and the Soneva Foundation, who also co-hosted the dialogue at their resort in the Maldives^17^. In retrospect, participants in this first dialogue noted that the multi-day, casual, retreat setting was critical in breaking down barriers between the CEOs and between CEOs and scientists. Living and eating together in a relaxed setting and at a beautiful place created the enabling conditions for establishing personal relationships and openness to collaboration. Subsequent steps and dialogues were planned, initiated and conducted in an emergent way, based on prior experience, active listening to participants, and advice from experts.

### Iterative interactions

All interactions were: (1) based on science, (2) respectful of other knowledge systems and sources of information, (3) mindful of diverging views and perspectives to provide conditions for learning, and (4) striving to develop a bold vision and action for social-ecological change in line with the SDGs, with a focus on the ocean. These four characteristics have contributed to developing an informal culture of trust, learning through respectful dialogue, and a shared sense of urgency, responsibility, and purpose. Interactions first strived to engage the CEOs in dialogue about the strategic importance of integrating ocean stewardship as a priority. Operational staff within the companies were later engaged (based on the same four principles), e.g., in task forces and working meetings, to ensure that CEO priorities could be operationalized.

During initial meetings, the scientific background consisted of a presentation of the keystone actor analysis^18^, and the hypothesis that such actors could potentially enable change, as a way to generate positive outcomes for ecosystems, people and business. These meetings were not dominated by science, but addressed knowledge and priorities of business representatives, and explored potential areas where collaboration with other keystone actors could be beneficial. To identify areas of particular interest, a central question for CEOs in these meetings was: *What problems, critical for your business, are you unable to solve by yourself, but could be solved in collaboration with the largest seafood companies in the world, and with support from science*? Answers to these questions included the identification of systemic and collective action problems such as IUU fishing, labour abuse, and unsustainable use of antibiotics, and helped identify priorities for the background materials and agenda for the first dialogue. Initial contacts provided space for dialogue about benefits and risks of engaging in collaboration, as well as the responsibilities of the largest actors to provide leadership for a healthy ocean^17^.

Initial interactions focused on developing a relationship of mutual understanding and trust and to eliminate suspicion or any potential animosity. These first meetings were followed by informal dialogues between scientists and CEOs, between the first and second keystone dialogue, aiming to identify key priorities by all companies out of the ten commitments defined at the first dialogue. Subsequent interactions facilitated learning, development of a shared vision, formalisation of a strategy, and engagement in, and evaluation of, concrete action. Scientists aimed to engage as non-partisan, and long-term knowledge providers, and keystone actors expressed an intention to strive for leadership in ocean stewardship. Collaboration consequently relied on good faith [*bona fides*].

### Data collection

The paper describes an emergent process as we did not have a clear roadmap to follow, nor had prior literature provided insight into what factors would be significant or should be tracked. Due to the novelty of the keystone actor and dialogue approach, it was impossible to specify in advance what research evidence should be collected. This led to as much data being gathered as possible. Our main goal was to understand if the nature and frequency of interactions between collaborators changed as SeaBOS activities developed, along with the underlying reasons for the observed dynamics.

The scientific background briefs and other documents provided updates on the status of individual companies in relation to their commitments. Meeting notes and summaries from keystone dialogues and other interactions, progress reports by SeaBOS members, weekly reports from the managing director, press releases, a project logbook (inspired by^64^), and findings from previous studies^6,12,13,16,17,49^, were also used to analyse the process.

To collect information on progress of individual companies and to evaluate the hypothesis that keystone actor companies could generate cascading change, we collected data at three different levels: the *individual*, the *keystone actor company*, and the *seafood industry*. Individual data collection consisted of interviews with participating company representatives (CEO or other staff), with a focus on identifying the perceived values, benefits and risks of collaborations. Data collection at the company level included regular assessment of publicly available reporting, dates, baselines and level of ambition in company targets, policies in place, and expression of priorities by the CEOs as identified in annual corporate reporting. We also used the internal (and previously unpublished) annual reports (2018–2020) produced to inform HRH Crown Princess Victoria of Sweden on progress (Supplementary Data S2).

Data collection at the industry level included assessment of the SeaBOS companies in relation to the remaining companies in a sample of 30 seafood companies regularly assessed by the World Benchmarking Alliance (The Seafood Stewardship Index)^55^ and also by using historical data collected during 2016 and 2017 by a seafood industry expert (B. Charron, Seafood Intelligence) on a larger set of corporations. We also assessed to what extent SeaBOS appeared to have an influence on, or inspire, initiatives in other industries, in policy or in science (by reviewing reports from ocean industry initiatives, ocean policy documents and the scientific literature citing the work on keystone actors).

An internal process among scientists was also initiated to improve the capacity for learning and reflection, where an external scientist conducted regular interviews with multiple members of the science team. These data sets were primarily used for internal communication with companies and between scientists, and were also used to assess progress among companies as reported in this study. Although special care was taken to identify evaluation criteria for progress that were generic to all companies, we also collected data that only applied to companies involved in aquaculture, feeds or wild capture fisheries. The progress reported here should be treated as preliminary, and a more comprehensive evaluation framework (and its subsequent application to data) is currently in development. To evaluate potential cascading effects is a subject of future studies.

To understand the efforts required by participants and the changing network of collaboration, we collected information on all science-business interactions between 2015 and 2021, including meeting date, type of meeting (virtual or physical), participants, affiliation, meeting location, travel-related carbon emissions, and whether it was associated to a task force. The interactions included six global keystone dialogues, four global working meetings, other working meetings, task force activities, company visits, internal or public events, and shared meals. They ranged from short telephone calls to full day meetings. Interactions that exclusively included scientists or industry members were not included, nor were interactions with individuals based in scientific institutions with administrative or communication tasks (although their support was critical).

### A global advocate for change

HRH Crown Princess Victoria of Sweden has been, and continue to be, engaged in several interactions, where She is providing leadership, vision, encouragement, thoughtful criticism, and a long-term perspective. Her opening remarks at keystone dialogues are available at https://www.kungahuset.se. HRH actively helped build a global and positive recognition of SeaBOS, while also incentivizing participants to engage in ambitious actions and reflection. The first interactions with HRH started several years prior to this initiative, in a series of knowledge exchanges between Her and members of the science team. As HRH assumed a role as global advocate for the SDGs, scientists approached Her for possible engagement in the keystone dialogues process. An agreement to participate was associated with a series of lectures given to Her to ensure development of relevant knowledge and expertise.

### Ambassadors, advisors and partners

To access the social networks of the executives of keystone actors, we engaged individuals formally employed as, or conducting a function similar to that of, ambassadors in Norway, South Korea and Japan^12^. These individuals volunteered to make introductions and facilitate initial meetings, and played instrumental roles in successfully connecting scientists to relevant networks and individuals. Advisors were engaged during the keystone dialogues to provide strategic support and guidance. We engaged with science, industry and civil society organizations with expertise in sustainable seafood, to learn from, and explore potential synergies between their activities. Special care was taken to ensure that they were driven by science, and operating with a collaborative culture.

### Law, money and ethics

All interactions paid strict attention to respecting anti-trust provisions and to ensure that conversations between companies complied with existing laws, including through regular engagement with lawyers.

Scientific activities were carried out by natural and social scientists and were funded by three US-based philanthropic organizations as well as employing academic institutions through core capability funding. This independent funding enabled contracting individuals with expertise to advance work in task forces and also to support the development and monitoring of the initiative. The Soneva Foundation generously hosted the first dialogue (providing room and board for all participants) and participants paid for their own travel to and from this meeting. The second dialogue was hosted by the Stockholm Resilience Centre and the Royal Swedish Academy of Sciences, and subsequent in person dialogues were hosted by participating companies. Hosting a meeting typically involved covering costs for the meeting venue and meals. All participants paid for their own travel and hotels. Participating scientists were guided by their respective university rules (e.g., associated with bribery) and did not receive any funds for any of their activities from seafood companies or the seafood industry.

This research was reviewed by the Stockholm Resilience Centre Research ethics sub- committee, who agreed with the assessment and plan for research ethics, and that the research did not require assessment by the Swedish Ethical Review Authority according to Swedish law. Written and oral consent was obtained for the use of personal or sensitive data from companies, and all data were analyzed and presented anonymously.

### Statistical analysis

We analysed the information on all interactions, divided in phases that generally corresponded to the annual cycle of SeaBOS meetings (one working meeting, one CEO-meeting, and multiple interactions between them). Participants (from industry) were anonymized, pooled and visualised in a social network as one symbol for each company (owner and subsidiary company are indicated as one colour) and the SeaBOS secretariat. Scientists from multiple institutions are illustrated as one colour. Individuals participating in the same meeting are described as connected in the social network. The evolving networks of science and business representatives illustrate individual participants engaged in each phase. The size of the nodes corresponds to the number of interactions that each individual engaged in during the corresponding phase. The frequency of interactions and the number of participants were averaged for each phase. Carbon emissions (estimated using https://traveltracker.world) and the scientific budget were summed for each phase.

We used networks and relied on a few widely-used metrics as descriptive tools to characterize the evolving features of collaboration as it unfolded. Local and global centrality metrics were used to describe the role of individual actors in the network and how interactions changed over time. Degree centrality is the number of connections an actor has, and it is a common metric of how connected an actor is locally. Betweenness centrality is related to the average number of shorter paths that transit through a particular actor, and is a proxy of its global importance. Density is the number of connections realized over all possible connections. We used the Bonacich power centralization^65^, a common metric in the social sciences to approximate how power and influence is distributed across a network. We used an exponent of 3 (rather than the default 1) to account for influence across multiple nodes (in our case, meetings).

## Supplementary Information

Supplementary Information.

## Data Availability

All data are available in the main text or the supplementary materials.
