# Supplementary material for: Scientific mobilization of keystone actors for biosphere stewardship
Source: Sci Rep. 2022 Mar 4;12:3802. doi: 10.1038/s41598-022-07023-8 (PMC8897441; doi:10.1038/s41598-022-07023-8)
Supplement: Supplementary file 1 — Supplementary Information. [file 41598_2022_7023_MOESM1_ESM.pdf]

Supplementary Materials for

Scientific mobilization of keystone actors for biosphere stewardship

Henrik Österblom*, Carl Folke, Juan Rocha, Jan Bebbington, Robert Blasiak, Jean-Baptiste Jouffray, Elizabeth R. Selig, Colette C.C. Wabnitz, Frida Bengtsson, Beatrice Crona, Radhika Gupta, Patrik J. G. Henriksson, Karolin A. Johansson, Andrew Merrie, Shinnusuke. Nakayama, Guillermo Ortuño Crespo, Johan Rockström, Lisen Schultz, Madlen Sobkowiak, Peter Søgaard Jørgensen, Jessica Spijkers, Max Troell, Patricia Villarubia-Gómez, Jane Lubchenco

*Corresponding author. Email: henrik.osterblom@su.se

This PDF file includes:

Supplementary Table S1: The keystone actors summarized
Supplementary Table S2: SeaBOS task forces – priorities and outcomes
Supplementary Table S3: Informal and formal partners
Supplementary Fig. S1: The combined engagement in meetings of participating organisation
Supplementary Data S1: Agendas of meetings, including information on the scientific background material provided (2016-2021) and associated public commitments
Supplementary Data S2: Annual Reports to HRH Crown Princess Victoria of Sweden (2018-2020)
Supplementary Data S3: Research Protocol for SeaBOS
Supplementary Data S4: Secretariat and SRC Operational Principles

Supplementary Table S1. The keystone actors summarized. Companies identified in Österblom et al.[1], and/or engaged in SeaBOS. The Mitsubishi acquisition of Cermaq (October 2015) motivated their inclusion in the first keystone dialogue although not part of the initial 13 keystone actors. Parent company Mitsubishi Corporation is not a member, but is participating in some of the meetings. Revenues and rank (by seafood revenues) estimated by Geerts et al.[2].

**Company**	**Revenues and rank**	**Headquarters**	**Main market**	**Main segment**	**SeaBOS membership**
Maruha Nichiro Corporation	7.2 mUSD (1)	Tokyo, Japan	Asia	Whitefish, tuna, shrimp	Nov 2016
Nissui	5.7 mUSD (2)	Tokyo, Japan	Asia	Whitefish, tuna, pelagics	Nov 2016
Thai Union	3,8 mUSD (3)	Samutsakorn, Thailand	USA	Tuna, pelagics, shrimp	Nov 2016
Mowi (previously Marine Harvest)	3,7 mUSD (4)	Bergen, Norway	Europe	Salmon	Nov 2016
Cermaq (subsidiary of Mitsubishi Corporation)	3,4 (5) (Mitsubishi Corporation)	Oslo, Norway (Tokyo Japan)	Europe	Salmon	Nov 2016
Dongwon Group	3.2 mUSD (6)	Seoul, South Korea	Asia	Tuna, pelagics	Nov 2016
Skretting (subsidiary of Nutreco)	2,5 mUSD (8) (Nutreco)	Stavanger, Norway (Amersfoort, Netherlands)	Europe	Feeds	Nov 2016
Trident Seafood	2,4 mUSD (9)	Seattle, USA	USA	Whitefish	Not a member
Austevoll Seafood	2,2 mUSD (10)	Storebö, Norway	Europe	Pelagic, salmon	Not a member
Kyokuyo Co. Ltd.	2,1 mUSD (11)	Tokyo, Japan	Asia	Tuna, pelagics	April 2017
Cargill Aqua Nutrition (previously EWOS)	2,1 mUSD (12)	Minneapolis, USA (Bergen, Norway)	USA	Feeds	Nov 2016
Charoen Pokphand Foods	1,9 mUSD (13)	Bangkok, Thailand	Asia	Feeds, shrimp	May 2017
Nueva Pescanova	1,1 mUSD (18)	Pontevedra, Spain	Europe	Shrimp, whitefish	Not a member
Pacific Andes	Unknown	Hong Kong, China	Asia	Whitefish	Not a member

Supplementary Table S2. SeaBOS task forces – priorities and outcomes.

Beijer = Beijer Institute for Ecological Economics, CAN = Cargill Aqua Nutrition, COS = Stanford Centre for Ocean Solutions, CPF = Charoen Pokphand Foods, DW = Dongwon, KVA = Royal Swedish Academy of Science, LU = Lancaster University, MNC = Maruha Nichiro Corporation, NSK = Nissui, SRC = Stockholm Resilience Centre, TU = Thai Union.

Task Force (TF) and leadership	Priority commitment	Main outcomes
TF I: Reducing IUU fishing and eliminating modern slavery.<br><br>SeaBOS; DW, Skretting, MNC, CPF<br><br>Science: SRC, COS, LU	Engage in concerted efforts to help reduce IUU fishing and seek to ensure that IUU products and endangered species are not present in our supply chains.<br><br>Engage in concerted efforts to eliminate any form of modern slavery including forced, bonded and child labor in our supply chains.	Development of scientific risk map (2017-2021)<br><br>Developments of voluntary actions in high-risk areas (2018-2021), piloted with companies in 2020-2021<br><br>Publication of a toolkit for tackling IUU fishing and labour issues (2020)<br><br>Piloting of block chain technology in Mexican sardine fishery (2018-2019)<br><br>Collaborative learning about code of conduct between companies (2017-2018)<br><br>CAN commit 700 suppliers of raw material to their code of conduct (2018)<br><br>Time-bound goals for reducing IUU fishing and modern slavery in own operations by Oct 2021 and for announcing measures in Dec 2020 that applies to tier 1 and 2 suppliers.
TF II: Improving transparency and traceability in global seafood<br><br>SeaBOS: TU, Skretting<br><br>Science: KVA	Improve transparency and traceability in our own operations, and work together to share information and best practice, building on existing industry partnerships and collaborations.	Collaborative learning of materiality assessment between companies (2017-2018), resulting in all members performing materiality assessments from 2020<br><br>Transparent reporting using GRI standard by all companies from 2020 (KK and MNC reports in line with GRI for first the time in 2018 and 2019 respectively, with NSK and DW both producing their first GRI reports in 2020<br><br>Skretting disclose production volumes with ODP from 2015, with CAN and TU establishing this practice in 2017 and 2021 respectively<br><br>Formal partnership with GDST established in 2018<br><br>Collaborative learning about traceability through partnership with GDST from 2018 and ongoing<br><br>NSK makes first ever inventory of raw materials in supply chain public in 2019[3], which is later mimicked by MNC (published in Japanese in 2021)[4] and KK (scheduled for publication in 2022)

TF III: Working with governments to improve regulations<br><br>SeaBOS: CAN, Nissui, DW, CPF<br><br>Science: SRC, Beijer	Engage in science-based efforts to improve fisheries and aquaculture management and productivity, through collaboration with industry, regulators and civil society.	Formal partnership with UNGC established in 2019<br><br>Formal partnership 2020: SeaBOS, CAN, Cermaq, Mowi, NSK, TU become members of advisory network to the High Level Panel on a Sustainable Ocean Economy (2019-2020)<br><br>Formal partnership 2021: SeaBOS publicly announces membership in "meta-coalition" as a means to advocate for ratification of PSMA<br><br>Cooperation with multiple UN agencies, including the FAO (from 2017, ongoing)<br><br>Policy statement 2017: Communication directed at governments (2nd statement), see Supplementary Text S1<br><br>Policy statement 2018: MNC, NSK and KK leads engagement with Japanese Government in 2018 (3rd statement), see Supplementary Text S1<br><br>Policy statement 2019: Letter in Financial Times advocating for ratification of the UN Agreement on Port States Measures (PSMA), co-signed by SeaBOS Chairman and Norwegian Prime Minister[5]<br><br>Policy statements 2020: CAN, Skretting and Mowi advocates for science-based fisheries quotas in the North Sea[6], and SeaBOS calls on governments to support boat crews and workers due to challenges associated to COVID-19 pandemic[7].<br><br>Policy statements 2021: SeaBOS publish joint statement with "meta-coalition" advocating for ratification of PSMA (2021)[8], members endorse Japanese policy development to address IUU fishing[9], and advocate for stronger policy action to address climate change with Environmental Defence Fund[10].<br><br>Establishment of time-bound goals for developing a plan for reducing/phasing out prioritised antibiotics and a code of conduct for antibiotics use by Oct 2021
TF IV: Vision, strategy, monitoring and communication<br><br>Cermaq, NSK<br><br>Science: SRC	Support novel initiatives and innovations for ocean stewardship	Development and agreement of articles of association, budget, recruitment profile and recruitment of managing director, and multiple internal policies (2018-2020)<br><br>Establishment of joint communications network (2020) and strategy (2021)

TF V: Reducing Plastic in seafood supply chain<br><br>SeaBOS: TU, Mowi, KK<br><br>Science: SRC	Reduce the use of plastics in seafood operations, and encourage global efforts to reduce plastic pollution.	Sharing of knowledge and approaches to quantify and reduce the use of plastics in seafood supply chains (2019-2020)<br><br>Formal partnership with GGGI in 2019[11]<br><br>Agreement to report on plastic packaging by 2021
TF VI: Climate Resilience<br><br>SeaBOS: Mowi, MNC, Cermaq, CAN<br><br>Science: SRC, KVA, Beijer	Reduce our own greenhouse gas emissions.	Work to identify scientific partners, scope of work, best practice and level of ambition (2019-2021)<br><br>Agreement to establish science-based goals for greenhouse gas emission reduction by October 2021 and to include scope 3 emissions by 2022.

Supplementary Table S3. Informal and formal partners. Organisations engaged by scientists (2015-2021), ranging from conversations aimed to explore synergies to engagement as formal partners (in bold). The SeaBOS secretariat engaged with additional organisations during Phases V and VI.

Phase I	**FFF (Forum For the Future)**, FFTF (Fishing for The Future), MSC (Marine Stewardship Council), O5 (Oceans 5), Ocean Unite, Sanford, SFP (Sustainable Fisheries Partnership), **Soneva (Soneva Foundation)**, SWE (Swedish Government), WWF (World Wide Fund for Nature)
Phase II	Fishtracker, **FFF,** FFTF, GP (Greeenpeace), JFA (Japanese Fisheries Agency), JFFA (Japan Forum Fisheries Agency), O5, SFP, **Soneva**, SSI (Seafood Stewardship Index), SWE, WEF (World Economic Forum), WOC (World Ocean Council), WWF
Phase III	5Gyres, AB InBev (Anheuser-Busch InBev), Conservation Alliance, ASC (Aquaculture Stewardship Council), DIHR (The Danish Institute for Human Rights), EDF (Environment Defence Fund), EJF (Environmental Justice Foundation), FAO (Food and Agriculture Organisation of the United Nations), Fishhacker, Fishtracker, FishWise, FiTI (Fisheries Transparencey Initiative), FOA (Friends of Ocean Action), **GDST (Global Dialogue on Seafood Traceability)**, GFW (Global Fishing Watch), GGGI (Global Ghost Gear Initiative), GRI (Global Reporting Initiative), GR Japan, GP, GSI (Global Salmon Initiative), GSSI (Global Seafood Sustainability Initiative), ICMM (International Council on Mining & Metals), IFPRI (International Food Policy research Institute), ILO (International Labour Organisation), ICI (International Cocoa Initiative), IMCS (International Monitoring, Control and Surveillance Network), IOM (International Organization for Migration), MBA (Monterrey Bay Aquarium), MSC, O5, OA (Ocean Action), PEW (The Pew Charitable Trusts), PRI (Principles for Responsible Investments), Seafood Intelligence, Seafood Legacy (SL), SOA (Sustainable Ocean Alliance), SSI, TNC (The Nature Conservancy), UK MSC (United Kingdom Modern Slavery Commission), UNGC (United Nations Global Compact), UNODC (United Nations Office of Drugs and Crime), WorldFish, WWF
Phase IV	ASC, EDF, Fishtracker, FishWise, FOA, **GDST**, **GGGI**, GSSI, **HLP (High Level Panel for a Sustainable Ocean Economy)**, ILRF (International Labor Rights Forum), ISSF (International Seafood Sustainability Foundation), KSAT (Kongsberg Satellite Services), MBA, MSC, ORRAA (Ocean Risk and Resilience Action Alliance), PEW, Saveoursons, SL, SFP, SSI, SWE, TMT (Trygg Mat Tracking), **UNGC**, WEF, WWF
Phase V	**GDST, GGGI**, GTA, **HLP**, IMCS, ISSF, KSAT, ORRAA, SL, SFP, SSI, **UNGC**
Phase VI	Birdlife International, European Commission, FAO, **GDST,** GSSI, GTA, **HLP,** International Union for the Conservation of Nature (IUCN), ISSF, Marine Trust, MBA, PEW, Science-Based Target Initiative (SBTi), SL, SFP, **UNGC**

Supplementary Fig. S1. The combined engagement in meetings of participating organisation Person-meetings were determined by multiplying the number of individuals participating from each organisation with the number of meetings they participated in. Note the difference in scale of the y-axis. The colour of each organisation corresponds to those of Fig. 1.

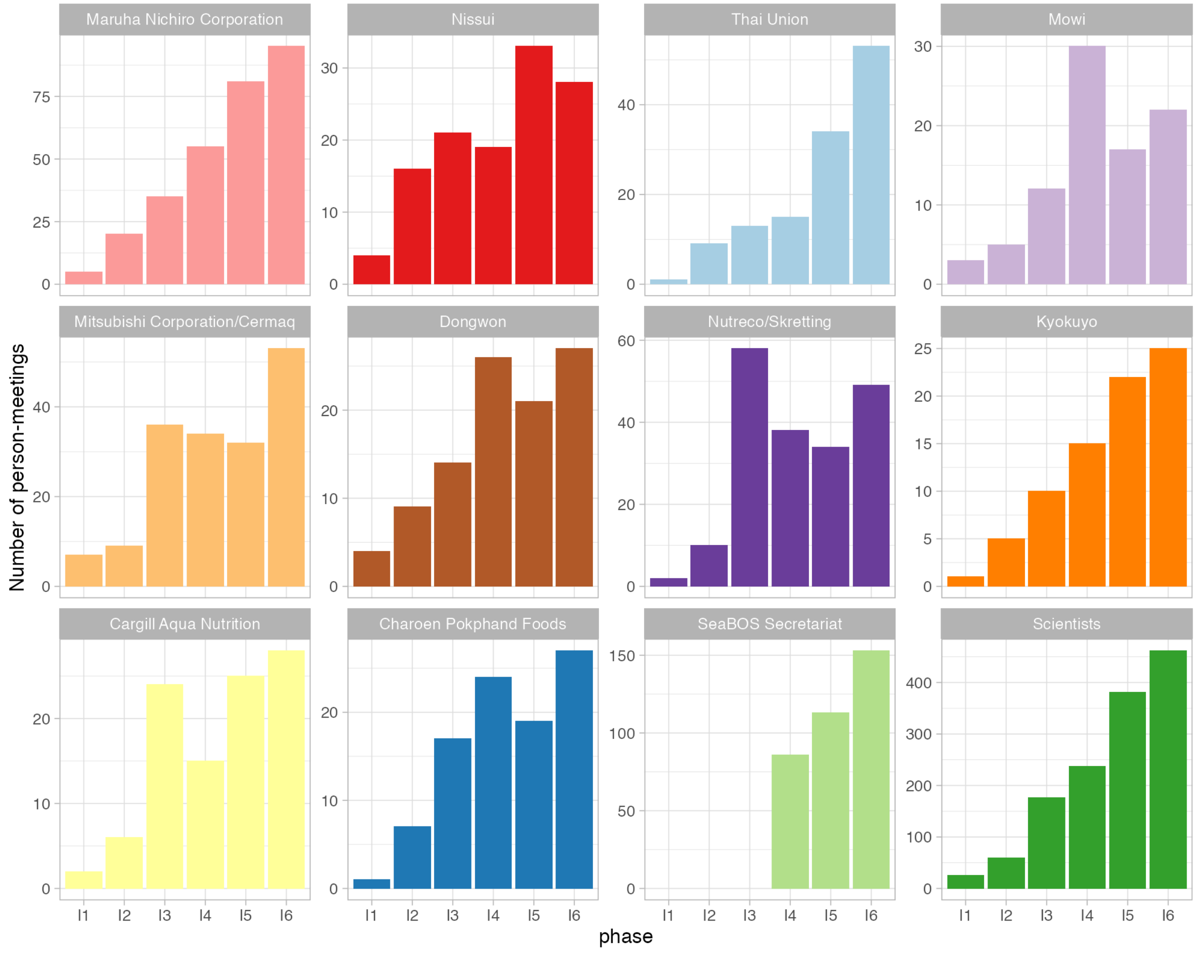

Supplementary Data S1: Agendas of meetings, including information on the scientific background material provided (2016-2021) and associated public commitments.

THE
SONEVA
DIALOGUE

PROGRAM

Transformative Risks and Opportunities for the Global Seafood Industry

Saturday 12th November

SESSION 1: SETTING THE SCENE
(*Background briefs 1 and 5*)

09:00 to 09:15	Introductions
09:15 to 09:25	Expectations for the Dialogue [redacted]
09:25 to 09:50	People and the planet – climate change, planetary boundaries and the United Nations Sustainable Development Goals [redacted]
09:50 to 10:25	General discussion
10:25 to 10:30	Summary of principal conclusions

SESSION 2: LEADERSHIP PERSPECTIVES

11:00 to 12:00	Grand challenges for the seafood industry [redacted]
	Reflections from advisors ([redacted]
12:00 to 12:30	General discussion on industry priorities within the wider global context

SESSION 3: SUSTAINABILITY ISSUES IN THE SEAFOOD INDUSTRY
(*Background briefs 2, 3 and 4*)

14:30 to 14:50	The global protein challenge [redacted]
14:50 to 15:10	Status and trends of wild capture fisheries [redacted]
15:10 to 15:30	Status and trends of aquaculture industry [redacted]
15:30 to 16:30	Breakout groups for more detailed discussions

Sunday 13th November

SESSION 4: INDUSTRY RESPONSES
(*Background briefs 6, 7 and 9*)

09:00 to 09:45 Governance and regulations ([redacted]

09:45 to 10:30 Innovations and market dynamics [redacted]

10:30 to 10:45 Ocean plastics [redacted]

10:45 to 11:15 Break for refreshments

SESSION 5: CORPORATE LEADERSHIP IN A DISRUPTED WORLD
(*Background brief 8*)

11:15 to 11:45 Corporate sustainability leadership – lessons from other sectors [redacted]

11:45 to 12:45 Breakout groups

12:45 to 13:00 Feedback from breakout groups

SESSION 6: NEXT STEPS – BRINGING IT TOGETHER

14:30 to 15:15 Perspectives from CEOs and advisors on leadership opportunities

15:15 to 15:30 Potential for industry leadership – defining thematic groups

15:30 to 16:30 Thematic group sessions

16:30 to 17:00 Report back and next steps

List of background briefs

Background brief 1: People and the Planet
Background brief 2: The Global Protein Challenge
Background brief 3: Wild Capture Fisheries
Background brief 4: Aquaculture
Background brief 5: Climate Change
Background brief 6: Governance and Regulations
Background brief 7: Innovations and Markets Dynamics
Background brief 8: Corporate Sustainability Leadership
Background brief 9: Ocean Plastics

Joint Statement from the 1st Keystone Dialogue

SEAFOOD BUSINESS FOR OCEAN STEWARDSHIP

PREAMBLE

We represent eight of the world's largest seafood companies, collectively operating in all segments of marine fisheries and aquaculture production, across the entire world. As leaders in the global seafood industry, we are concerned about the state of the ocean and the global environment.

We depend on a stable and resilient planet for human prosperity. However, science is already providing evidence that we have entered the Anthropocene, an epoch where humanity is now challenging the stability of Earth and its ocean.

We, as keystone actors in the global seafood industry[1], recognize that together we represent a global force, not only in the operation of the seafood industry, but also in contributing to a resilient planet with marine ecosystems continuing to produce food of high quality for present and future generations.

We already make a significant contribution to healthy and nutritious diets, as well as to employment all around the world, helping to provide food security for all. We are confident that an increased production of seafood – caught in a healthy ocean using sustainable fishing methods or farmed in sustainable production systems, by people employed in safe and fair working conditions – is both possible and critical for the future of humankind.

However, oceans are under enormous pressure. There is strong scientific evidence of growing impacts on marine ecosystems. Ocean temperatures and acidification are increasing; degradation of coastal mangroves and coral reefs is threatening critical life support systems; habitats are being destroyed; nutrient run-off and toxic substances are causing serious pollution; and the build-up of plastic waste in the oceans is a threat to many species and to human health.

Many of these challenges in the oceans are not caused by the seafood industry itself, but they all impact us directly and indirectly.

We acknowledge that the ocean is also directly affected by activities of wild capture fisheries, such as Illegal, Unreported and Unregulated (IUU) fishing, bycatch, overfishing and modern slavery.

In the face of a growing and wealthier world population, the reliance on aquaculture as a crucial contributor to sustainable food production will increase. However, badly managed, aquaculture can have detrimental social and environmental impacts.

1 Keystone actors dominate global production revenues and volumes, control important segments of production, connect ecosystems globally through subsidiaries and influence critical governance processes and institutions, see: Österblom, H. et al. 2015. PLoS ONE 10(5): e0127533 http://journals.plos.org/plosone/article?id=10.1371/journal.pone.0127533

THE WAY FORWARD

We are committed to playing a leading role in addressing these challenges. As keystone actors, we are resolved to set up a new global initiative – "*Seafood Business for Ocean Stewardship*" – that brings together, for the first time, leaders in science and business, the wild capture and aquaculture sectors of the seafood industry, and companies from Asia, Europe and North America, operating globally.

We fully support and endorse the UN Sustainable Development Goals (SDGs) as a new framework for economic and social development operating within the capacity of the biosphere and its ocean. Not only do we urge all governments to implement the SDGs, we also encourage businesses to integrate them in their strategies. For us, it is particularly important that governments should ensure the right conditions for sustainable fisheries, aquaculture and a healthy ocean. Measures aimed at implementing the Paris Agreement on climate, reducing pollution and halting the destruction of coastal habitats also need to be urgently implemented.

OUR COMMITMENTS

Efforts by governments are critical, but the industry has an equally important role to play in developing and implementing solutions. Leadership in corporate sustainability is a priority for us. We believe this enhances our operations, helps make the industry more resilient, improves our access to markets and consumers, attracts new financial flows, and makes us more attractive as employers. We know better than anyone that the seafood industry depends on sustainable use of the ocean for long-term value creation.

Our companies have been in the seafood business for decades, some for more than a century. We take pride in what we already do, but acknowledge that we ourselves have further to go in improving our performance. For us, this is a strategic and long-term commitment.

Through "*Seafood Business for Ocean Stewardship*", we intend to develop a common understanding and a common approach among the keystone actors globally, building on the many existing initiatives in which our companies are already engaged.

This is an initiative between science and business, with an ambition to engage with governments and other stakeholders for positive change. It is not only about supplying sustainable seafood to consumers; it is about becoming stewards of the world's ocean and aquaculture environments.

We will act on the following:

- Improve transparency and traceability in our own operations, and work together to share information and best practice, building on existing industry partnerships and collaborations.
- Engage in concerted efforts to help reduce IUU fishing and seek to ensure that IUU products and endangered species are not present in our supply chains.
- Engage in science-based efforts to improve fisheries and aquaculture management and productivity, through collaboration with industry, regulators and civil society.
- Engage in concerted efforts to eliminate any form of modern slavery including forced, bonded and child labour in our supply chains.
- Work towards reducing the use of antibiotics in aquaculture.
- Reduce the use of plastics in seafood operations, and encourage global efforts to reduce plastic pollution.
- Reduce our own greenhouse gas emissions.

- Secure new growth in aquaculture, by deploying best practices in preventive health management, including improved regulatory regimes.
- Collaborate and invest in the development and deployment of emerging approaches and technologies for sustainable fisheries and aquaculture.
- Support novel initiatives and innovations for ocean stewardship.

The potential of the ocean to help provide healthy food for hundreds of millions of people is tremendous. But bold and firm action will be needed to make this happen. As the keystone actors in the international seafood industry, depending as we do on a healthy and resilient ocean, we know we can and must make a major contribution.

The seafood industry cannot thrive on an unsustainable planet, and we will not have a thriving planet with an unsustainable seafood industry. Now is the time to step up together as proactive leaders for ocean stewardship[2].

---

2 Ocean stewardship is an adaptive and learning based, collaborative process, of responsibility and ethics, aimed to shepherd and safeguard the resilience and sustainability of ocean ecosystems for human well-being.

SIGNATORIES

Shigeru Ito
PRESIDENT
Maruha Nichiro Corporation

Norio Hosomi
PRESIDENT AND CEO
Nippon Suisan Kaisha, Ltd

Thiraphong Chansiri
PRESIDENT AND CEO
Thai Union Group

Ole-Eirik Lerøy
CHAIRMAN OF THE BOARD
Marine Harvest ASA

Myoung Woo Lee
CEO
Dongwon Industries

Knut Nesse
CEO AND CHAIRMAN
Nutreco (owner of Skretting)

Einar Wathne
GROUP LEADER AND PRESIDENT
Cargill Aqua Nutrition

Geir Molvik
CEO
Cermaq (subsidiary of Mitsubishi Corporation)

海洋管理のための水産事業

前文

我々は世界最大の水産企業8社を代表しており、事業の範囲は世界中の海洋漁業および水産養殖のあらゆる分野を網羅しています。我々は世界の水産業界のリーダーとして、海洋と地球環境の状態に懸念を抱いています。

人類の繁栄にとって、安定した、回復力のある[i]地球環境は不可欠です。しかし、いまや人新世（アントロポセン）[ii]、つまり人類が地球の安定と海を脅かす時代に入っているという科学的根拠があります。

世界の水産業界の命運を握るキーストーン[iii]アクター[1]として、我々は業界の経営だけでなく、現在と将来の世代に継続的に高品質な食糧を生産する海洋生態系を有する地球の回復力に対しても、国際的な影響力を持っていると認識しています。

既に我々は、世界中でヘルシーで栄養価の高い食生活ならびに雇用に大いに貢献しており、すべての人々への食糧安全保障における役割を担っています。そして、安全で公平な労働条件の下、持続可能な漁法による健全な海での漁業と、持続可能な生産システムによる養殖により、水産物の生産を増やすことは可能であり、かつ人類の未来にとって不可欠であると確信しています。

しかしながら、海洋への負荷は深刻なものです。海洋生態系への影響拡大を示す確固たる科学的証拠もあります。海水温の上昇および海洋の酸性化、沿岸域のマングローブやサンゴ礁の劣化が重要な生命維持システムを脅かしています。生息域は次々に破壊されており、富栄養化や有害物質が深刻な汚染を引き起こしています。また、海洋におけるプラスチックごみの蓄積は、多くの生物種、そして人間の健康にとっても脅威となっています。

海洋におけるこうした課題の多くは水産業界そのものに起因するものではないものの、その影響を我々は直接的および間接的に被ります。

我々は、違法、無報告、無規制のIUU漁業や混獲、過剰漁獲、現代の奴隷制ともいえる強制労働といった天然漁業における活動が、海洋に直接的な影響を及ぼしているという認識を持っています。

世界的な人口増加および富裕層の拡大により、持続可能な食糧生産を担う水産養殖への依存が増すことになります。しかし、不適切な管理の下での水産養殖は、社会および環境への被害をもたらすことにもなりかねません。

1 キーストーンアクターは、世界的な生産において利益および数量で大半を占め、生産において重要なセグメントをコントロールし、子会社を通じて世界的にエコシステムをつなぎ、重要な管理プロセスおよび組織に対して影響力を持つ。参照： Österblom, H. et al. 2015. PLoS ONE 10(5): e0127533 http://journals.plos.org/plosone/article?id=10.1371/journal.pone.0127533

今後の対応

我々は、こうした課題を率先して解決するために真摯に取り組みます。我々は、水産業界のキーストーンアクターとして、科学やビジネス、天然漁業と養殖業、そして世界中で事業を展開しているアジア、ヨーロッパ、北米の企業のリーダーが集う、史上初のグローバルイニシアチブを立ち上げることを決意しました。それが Seafood Business for Ocean Stewardship （シーフード ビジネス フォー オーシャン スチュワードシップ：「海洋管理のための水産事業」の意）です。

我々は、国連の持続可能な開発目標（SDGs）が生物圏と海洋の許容範囲内での経済的および社会的発展のための新しい枠組みであると認識しており、これを全面的に支援し支持します。各国政府にSDGsの実施を促すだけでなく、企業に対しても戦略へ統合することを奨励します。我々にとって特に重要なことは、持続可能な漁業、水産養殖業、および健全な海のための適切な条件が各国政府によって確保されるということです。パリ協定の実施、汚染の低減および沿岸生息域の破壊を阻止するための措置も、緊急に行う必要があります。

我々の取組み

政府の取り組みは重要ですが、解決策の構築と実施における業界の役割も同様に重要です。企業の持続可能性への取り組みにおいてリーダーシップを発揮することは、我々の最優先事項の一つです。これにより、経営の向上、業界の回復力向上、市場や消費者へのアクセス向上、新たな資金フローの誘致、そして雇用主としての魅力増大にもつながるものと確信しています。水産業界における長期的な価値の創造は、海洋の持続可能な利用にかかっていることを我々は誰よりも認識しています。

我々は、何十年も前から水産業に携わってきました。中には100年以上の歴史を誇る企業もあります。我々はこれまでに行ったことに誇りを持っていますが、業績のさらなる向上が必要であることも認識しています。我々にとってこのことは、戦略的かつ長期的な取り組みなのです。

我々は、「海洋管理のための水産事業」を通じ、各企業が既に取り組んでいる多くの既存のイニシアチブを基に、世界のキーストーンアクター間で共通の理解とアプローチを構築する考えです。

これは科学とビジネスの間のイニシアチブであり、いずれは政府やその他の利害関係者を巻き込んでポジティブな変化を起こすことを目指しています。持続可能な水産物を消費者に供給するだけでなく、世界の海洋および養殖環境の管理者になるための取り組みです。

我々は以下のことに取り組みます

- 自社の業務における透明性とトレーサビリティを向上させ、既存の業界パートナーシップと協調の上に、情報およびベストプラクティス（最良事例）の共有に取り組む。
- IUUの漁業の減少と、IUUによる製品と絶滅危惧種がサプライチェーンに入らないようにするために協力して取り組む。
- 業界、規制機関および市民社会との協働により、漁業や水産養殖の管理および生産性を改善するための科学的根拠に則った活動に取り組む。
- サプライチェーンにおける現代の奴隷制ともいえる強制労働や身売り労働、児童労働などはいかなる形であろうと、それを排除するために協力して取り組む。
- 水産養殖における抗生物質の使用を減らすように努める。
- 水産業におけるプラスチックの使用を減らし、プラスチック汚染を減らすための世界的な取り組みを推進する。
- 自社の温室効果ガス排出量を削減する。

- 規制制度の改善を含む予防的健康管理のベストプラクティス（最良事例）を導入することによって、水産養殖の新たな成長を促す。
- 持続可能な漁業および水産養殖のための新たなアプローチや技術の開発および導入に協力し、投資を行う。
- 海洋管理における新しいイニシアチブとイノベーションを支援する。

数億人に及ぶ人々のために健康的な食糧を提供することができる海の持つ可能性は計り知れません。しかし、そのためには、大胆かつ確固たる行動が求められます。健全で回復力のある海洋に依存するグローバルな水産業界のキーストーンアクターとして、我々は大いに貢献できるものと自負しています。そしてまた、我々にはその責任があるのです。

持続不可能な地球環境では、水産業界の繁栄はありえず、持続不可能な水産業界では豊かな地球環境を育むことができません。今こそ、先を見越して海洋管理（オーシャンスチュワードシップ[2]）を積極的に進めるリーダーとして共に行動する時です[2]。

---

2 オーシャン スチュワードシップは、適応と学習に基づく協働プロセスであり、責任と倫理規範によって、人類の幸福のために海洋生態系の回復力と持続可能性を守ることを目指す取り組み。

---

[i] Resilience （回復力、強靭性）
レジリエンス（Resilience）とは「精神的回復力」「抵抗力」「復元力」「耐久力」などとも訳される心理学用語。元々はストレス (stress) とともに物理学の用語であった。 ストレスは「外力による歪み」を意味し、レジリエンスはそれに対して「外力による歪みを跳ね返す力」として使われ始めた。ストレスや逆境に直面したとき、それに対応し、克服していく（しなやかな）能力のこと。事業継続的な意味でも使われ、危機的状況からの柔軟な復旧能力ということ。

[ii] 人新世：ひとしんせい、じんしんせい＝Anthropocene（アントロポセン）とは、ノーベル化学賞受賞のドイツ人大気化学者、パウル・クルッツェンによって提案された造語で、人類が地球の生態系や気候に大きな影響を及ぼすようになった近年の地質学的な時代を表しています。 更新世の次の地質時代の、人類の時代という意味です。

[iii] キーストーンは頂上であることを示すと同時に、周囲の建材が崩れないように締める役目を持つ。この用語は、理論や組織など大きな構造を中心で支えている要素を指して比喩的に使われることがある。すなわち、それがないと構造全体が崩壊するという意味で使う。例えば群集生態学ではキーストーン種という語がある。

THE STOCKHOLM DIALOGUE

PROGRAM

Advancing the Seafood Business for Ocean Stewardship Initiative

Monday 15th May

SESSION 1: SETTING THE SCENE

09:00 to 10:30

Opening statement [redacted]

Welcoming statement [redacted]

Overview of the agenda and work flow [redacted]

Summary of the "keystone actors" process [redacted]

Industry progress in relation to SeaBOS – achievements and challenges [redacted]

Reflections from the host of the upcoming Ocean Conference in New York [redacted]

SESSION 2: SCIENTIFIC UPDATES

(*Background briefs 1 and 2*)

11:00 to 12:30

Trade, People and Ecosystems [redacted]

Seafood for Human and Planetary Health [redacted]

Ocean Stewardship – the grand challenge [redacted]

Discussion

SESSION 3: MOVING THE COMMITMENTS FORWARD (1)

14:00 to 15:00

Summary of bilateral conversations [redacted]

Articulating the rationale for identified priorities – reflections ([redacted]

SESSION 4: MOVING THE COMMITMENTS FORWARD (2)

15:30 to 16:30

Organizing in task forces

Initial exploration of vision, targets and actions

Tuesday 16th May

SESSION 5: MOVING THE COMMITMENTS FORWARD (3)

09:00 to 10:30 Summary of day one and agreed priorities [redacted]

Breakout discussions on task forces:

- Formulating vision and targets
- Identifying actions
- Knowledge gaps

SESSION 6: MOVING THE COMMITMENTS FORWARD (4)

11:00 to 12:30 Group presentations and discussion

Exploring potential procedures for monitoring, reporting and adaptation

SESSION 7: OPERATIONAL PROCEDURES

14:00 to 15:30 Location of secretariat

Funding mechanisms

Terms of reference for operations

SESSION 8: NEXT STEPS

16:00 to 17:00 Communication opportunities: The Ocean Conference in New York , The Stockholm EAT Forum, Our Ocean Conference in Malta [redacted]

Next meeting

Reflections and conclusions

List of background briefs

Background brief 1: Trade, People and Ecosystems
Background brief 2: Seafood for Human and Planetary Health

Seafood Business for Ocean Stewardship (SeaBOS)

OUR PLEDGE FOR OCEAN STEWARDSHIP – OUR PLEA TO GOVERNMENTS

PREAMBLE

Producing healthy food to feed more than nine billion people by 2050, while sustaining the biosphere, is one of the greatest challenges facing humanity. We are convinced that an expansion of global seafood production, including both from wild capture fisheries and aquaculture, represents a critical opportunity to achieve the UN Sustainable Development Goals (SDGs). Adequately regulated and managed seafood production, together with innovative technologies, will increase the supply of healthy food, which in turn can alleviate pressure on terrestrial food production systems.

Seafood already plays a central role in the global food portfolio, contributing 20 % of the global intake of animal protein. Capture fisheries – the only large-scale food production system based on harvest of a wild resource – provide half of global seafood. However, many improvements must be made for all wild-capture fisheries to be sustainable. While some regulated fisheries result in overfishing, the global community has so far failed to eliminate illegal, unreported and unregulated (IUU) fisheries, modern slavery on fishing vessels, and destructive impacts on habitats and non-target species. These problems could be addressed with better scientific information, more effective regulations and better mechanisms for monitoring and enforcing compliance. Governments also have much to learn from each other. Improved fishery management, sometimes including a reduction of fishing pressure over the short term, often corresponds to rebuilding of stocks and long-term benefits for both ecosystems and people. Global fisheries landings could increase substantially if stocks are managed properly.

Aquaculture has dramatically changed global seafood supply. The provision of healthy and sustainable seafood needs to increase further to meet future demand. However, the aquaculture sector is highly diverse in terms of environmental performance. To meet future needs, governance needs to address more comprehensively the challenges of feeds, pollution, spread of diseases and overuse of antibiotics.

Increasing the production of healthy and sustainable food from the ocean is not just about seafood production – it must rely on a foundation of ocean stewardship. Seafood producers from around the world, regardless of their scale of operations, have one thing in common: they all depend on healthy, functioning ecosystems. If the ocean is not managed in a sustainable way, fisheries and aquaculture will not be able to deliver its full potential.

Well managed fisheries and aquaculture, produced in resilient ecosystems, result in healthy and sustainable protein supplies. Adequate regulations and best practices are critical for sustaining and expanding future seafood production in ways that contribute to meeting the SDGs. There is no seafood industry in a dead ocean. And we believe there is no chance to meet the Global Goals without seafood.

WHO WE ARE

We, members of the Seafood Business for Ocean Stewardship (SeaBOS) initiative, represent nine of the largest seafood producers in the world. We operate on every continent and in all segments of seafood production. We are the global industry leaders in fisheries and aquaculture.

We work closely together with scientists to identify problems and solutions to achieve a healthy ocean and a planet where people from all parts of the world can eat healthy and sustainable seafood. Together, we represent a global force, with a unique ability to inspire business actors along the entire seafood value chain and support governments in achieving the SDGs. We are committed to use our combined power to lead by example, and to use our united voice to argue for change.

We will improve our own operations, and challenge the rest of the industry to follow. We will also offer our support to regulators, in order to ensure that fisheries, aquaculture, and the ocean at large, are managed in a sustainable way.

Our work started in November of 2016, when eight of us met for the first time in a global dialogue about the future of the ocean. This meeting, termed the Soneva Dialogue, resulted in a joint statement, published in December of 2016, where we expressed our intent to take on a leadership role for the ocean and when we announced the Seafood Business for Ocean Stewardship initiative. In May 2017, we met again, this time in Stockholm and with additional companies, in order to further develop this initiative.

OUR PLEDGE

We represent a young initiative, but are already able to make a number of strong commitments for ocean stewardship.

We pledge to work diligently to eliminate IUU products and any form of modern slavery in our supply chains. We will develop a code of conduct for our own operations and for our suppliers, in order to deliver on these commitments. We will also work towards full traceability and transparency throughout our supply chains.

We pledge to make efficient use of aquaculture feeds and to use fish feed resources from sustainably harvested stocks. We pledge to actively use and develop fish health management systems and health prevention methods before treatments. We will actively use and apply existing certification standards and prevent harmful discharges and habitat destruction. We call on the whole industry to do the same.

We also pledge to work actively together with governments to improve existing regulations for fisheries, for aquaculture, and for the ocean.

We will report on our progress with this work, one year from now, in June of 2018.

OUR PLEA

We strongly urge all governments to work together to address IUU fishing. In order for wild capture fisheries to further develop, we encourage governments to end overfishing and rebuild depleted stocks. This critically means respecting scientific advice on quotas and the sharing of best practices in regulations.

We urge all governments to work together to improve regulations in international waters. We call on all governments to sign up to the FAO Port States Measures Agreements (PSMA) and to develop a regulatory international treaty, analogous to the PSMA, which bans all landing of IUU fish, for UN members to ratify.

We urge governments to develop a shared framework for sustainable aquaculture. Governments have an important role to play in developing regulations that improve resource usage and manage diseases. It is critical to take this responsibility seriously. We also urge governments to actively share experiences between countries to improve existing policies.

We welcome new technologies and innovation that contribute to improved fisheries and aquaculture management, regulations and policy, as well as the monitoring of compliance to such regulations.

Finally, we demand that governments of the world address the challenges currently facing our industry, originating from outside of our sector. We are unable to safeguard and much less expand our production of healthy and sustainable protein to meet the needs of a growing and wealthier world population if toxic pollutants or plastic particles contaminate fish, if there is no clean water for our aquaculture operations nor if climate change, ocean acidification, and low dissolved oxygen levels undermine our long-term operations.

We will support you in all these efforts to the best of our ability.

SIGNATORIES

Shigeru Ito
PRESIDENT
Maruha Nichiro Corporation

Norio Hosomi
PRESIDENT AND CEO
Nippon Suisan Kaisha, Ltd

Thiraphong Chansiri
PRESIDENT AND CEO
Thai Union Group

Ole-Eirik Lerøy
CHAIRMAN OF THE BOARD
Marine Harvest ASA

Myoung Woo Lee
CEO
Dongwon Industries

Knut Nesse
CEO AND CHAIRMAN
Nutreco (owner of Skretting)

Einar Wathne
GROUP LEADER AND PRESIDENT
Cargill Aqua Nutrition

Geir Molvik
CEO
Cermaq (subsidiary of Mitsubishi Corporation)

Hisaki Tada
CHAIRMAN
Kyokuyo Co., Ltd

Adirek Sripratak
CHAIRMAN OF THE EXECUTIVE COMMITTEE
Chareon Pokphand Foods PCL

海洋管理のための水産事業 (SeaBOS)

海洋管理に対する我々の取り組み – 政府への嘆願

前文

生物圏を維持しながら、2050年までに90億人以上の人間のために健康的な食糧を生産することは、人類が直面している最大の課題の1つです。我々は、天然漁業と養殖業からの両方を含む、世界の水産物生産の拡大が、国連の持続可能な開発目標(SDGs)を達成する重要な機会であると確信しています。適切に規制、管理された水産物の生産は、革新的な技術とともに、健康的な食糧の供給を増加させ、地上の食糧生産システムに対する圧力を緩和するでしょう。

水産物は、既に世界の食品ポートフォリオにおいて中心的な役割を果たしており、動物性タンパク質の世界的摂取量の20%を占めています。捕獲漁業は、天然資源の捕獲に基づく、唯一の大規模食糧生産システムで、世界の水産物の供給量の半分を

占めています。しかし、すべての捕獲漁業が持続可能になるためには、多くの改善が必要です。規制された漁業でも過剰漁獲状態に陥っている場合もあり、我々の社会は、これまで、IUU(違法、未報告、無規制)漁業、漁船での現代の奴隷制 ともいえる強制労働、生息地および（混獲により意図せず捕獲される）目的外の生物種に対する破壊的影響を排除できていません。これらの問題は、より良い科学的情報、より効果的な規制、コンプライアンスの監視および実施のための、より良い仕組みで対処することができます。政府間で、お互いに学ぶべきこともたくさんあります。短期間の間漁獲圧を低減させるなどの漁業管理の改善は、資源の回復と生態系および人類両方にとっての長期的な便益となるのです。資源が適切に管理されれば、世界の漁業の水揚げ量は大幅に増加する可能性があります。

水産養殖は世界の水産物の供給を、劇的に変えました。健康的で持続可能な水産物の供給は、将来の需要を満たすためにさらに増加する必要があります。しかし、水産養殖部門は、環境問題への対応となると個々に差があります。将来のニーズに応えるため、飼料、汚染、病気の流行、抗生物質の過剰使用の課題に、ガバナンス強化によってより包括的に対処する必要があります。

海洋からの健康的で持続可能な食糧の生産を増やすことは、水産物の生産だけに関わることではありません – 海洋管理の基礎に頼る必要があるからです。世界各地の水産物生産者は、事業規模にかかわらず、共通点が1つあります。すべて、健康で機能している生態系に依存しているという点です。海洋が持続可能な方法で管理されていなければ、漁業および水産養殖はその潜在能力を完全に発揮することはできません。

適切に管理された漁業および水産養殖が、回復力のある生態系で行われれば、健康的で持続可能なタンパク質の供給につながります。十分な規制とベストプラクティスは、SDGsの達成に貢献すると同時に、将来の水産物の生産を維持、拡大するために非常に重要です。海が死んでしまえば、水産業界の存続はありません。また、水産物がなければ、持続可能な開発目標を達成する機会はないと信じています。

SEABOSとは

我々は、海洋管理のための水産事業(SeaBOS)イニシアチブの会員で、世界最大の水産企業9社を代表しています。我々は世界各地で操業し、事業の範囲は、水産業のあらゆる分野を網羅しています。我々は、世界の漁業、水産養殖業のリーダーです。

我々は科学者と緊密に協力し合い、海洋と地球を健全なものにするための問題と解決策を特定し、それにより世界中の人々が健康的で持続可能な水産物を口にすることができるようにします。我々は、水産業界のバリューチェーン全体で他の事業者への影響力を持ち、SDGs達成において政府を支援する独自の能力を備えたグローバルなグループです。我々は、結集させた影響力により模範を示すことで先例を示し、共同声明を出すことで変革を主張していきます。

我々は、自社の業務を改善し、業界他社にも追従するよう働きかけます。また、漁業、水産養殖、ひいては海洋全体が、確実に持続可能な形で管理されるよう、規制当局への支援も提供します。

我々の取組みは2016年11月、我々8社が海洋の将来についてのグローバルな対話をするべく一堂に会したことから始まりました。ソネバ・ダイアログと呼ばれるこの会議では、共同声明が2016年12月に出され、この声明文で海洋に対するリーダーシップの役割を果たす意思を表明し、海洋管理のための水産事業イニシアチブを発表しました。2017年5月には、このイニシアチブをさらに進展させるため、ストックホルムで2度目の会議を開催し、新たな企業も加わりました。

我々の取組み

我々の取組みは始まったばかりですが、既に海洋管理に関して数多くの強力なコミットメントを決めています。

サプライチェーンにおいて、IUU製品および現代的な奴隷制度を排除するために協力して取り組みます。これらのコミットメントを遂行するため、自社の事業およびサプライヤーのための行動規範を策定します。また、サプライチェーン全体での完全なトレーサビリティと透明性に向けて取り組みます。

水産養殖飼料を効率的に利用し、持続可能な漁法で捕獲された資源から魚の飼料資源を使用することを約束します。魚の健康管理システムおよび治療に至る前の予防の方法を積極的に使用、開発することを約束します。既存の認証基準を積極的に活用、適用し、有害廃棄物や生息域の破壊を防止します。業界全体に対し、同様な取組みをするよう呼びかけます。

また、漁業、水産養殖、海洋に対する既存の規制を改善するため、政府と積極的に連携することを約束します。

2018年の6月に、今から1年後のこの取組みの進捗状況を報告します。

我々の嘆願

IUU漁業に対処するため、すべての各国政府が協力することを強く要請します。天然漁業のさらなる発展のために、政府が過剰漁獲を終わらせ、枯渇した資源を回復させることを奨励します。これには、割り当て制度に関する科学的助言を尊重し、規制におけるベストプラクティスを共有することが非常に重要です。

国際水域の規制を改善するため、すべての各国政府が協力することを要請します。すべての政府に対し、FAO寄港国措置協定(PSMA)に署名し、国連加盟国が批准するため、IUU魚のすべての水揚げを禁止するPSMAに類似した国際条約規制を作成するよう求めます。

持続可能な水産養殖のための共通の枠組みを構築するよう、各国政府に要請します。政府は、資源の使用を改善し、疾病を管理する規制を構築する上で重要な役割を果たしています。この責任を真摯に受け止めることが重要です。また、既存の政策を改善するため、積極的に経験を各国間で共有するよう、各国政府に要請します。

漁業や水産養殖の管理、規制、政策の改善、新たな規制の遵守の監視に貢献する、新たな技術およびイノベーションを歓迎します。

最後に、水産業に起因する問題ではないものの、水産業が現在直面している課題に世界の政府が取り組むことを要求します。汚染物質やプラスチック粒子による魚の汚染、養殖事業に必要なきれいな水の欠如、気候変動、海洋酸性化や低溶存酸素レベルにより我々の長期的な事業が損なわれるのであれば、世界的な人口増加および富裕層の拡大による需要に応じるために、健康的で持続可能なタンパク質の生産を保全、拡大することは出来ません。

我々は、出来る限り、これらの取組みにおいて、政府をサポートします。

THE
AMERSFOORT
DIALOGUE

PROGRAM

Seafood Business for Ocean Stewardship

WORKING MEETING

Monday 14th May

SESSION 1: SETTING THE SCENE

10:30 to 11:15
- Opening statement [redacted]
- Overview of the agenda and work flow [redacted]
- Industry progress in relation to SeaBOS [redacted]

SESSION 2: TASK FORCE I - REDUCING IUU FISHING AND ELIMINATING MODERN SLAVERY

(*Background brief 1, Document 2*)

11:30 to 13:00
- Task Force I – Priorities and strategy [redacted]
- Modern slavery and IUU fishing [redacted]
- Draft global maps of hotspots for IUU fishing and modern slavery [redacted]
- Discussion
- Comparing SeaBOS members Codes of Conduct [redacted]
- Preliminary results from questionnaire [redacted]
- Piloting block-chain technology to address IUU fishing and modern slavery [redacted]
- Potential voluntary actions by SeaBOS members [redacted]
- Discussion

14:00 to 14:15
- Towards September for Task Force I – summary of next steps [redacted]

SESSION 3: TASK FORCE II – TRANSPARENCY AND TRACEABILITY

(*Documents 3, 4, 5*)

14:15 to 15:15
- Task Force II – Priorities and strategy [redacted]
- Global Dialogue on Seafood Traceability (GDST) [redacted]
- Transparency and Traceability - State of the art [redacted]
- Benefits of materiality analyses and reporting [redacted]
- Materiality analysis and raw materials sourcing [redacted]
- Discussion

SESSION 4: TASK FORCE II (CONTINUED)

15:30 to 16:30
- Towards September for Task Force II – summary of next steps [redacted]
- Ocean stewardship – what is it and how to do it? [redacted]
- Discussion

16:30 to 17:00
- Conclusion day 1 and outstanding questions

Tuesday 15th May

SESSION 5: TASK FORCE III – IMPROVING REGULATIONS

(*Document 6*)

09:00 to 10:00
- Task Force III – priorities and strategy [redacted]
- Development of Japanese legislation [redacted]
- The FAO process to develop global policies on antibiotics [redacted]
- The UN Global Compact [redacted]
- Speaking with a shared voice: a draft placeholder document [redacted]
- Discussion
- Decision on placeholder document
- Towards September for Task Force III – summary of next steps [redacted]

SESSION 6: TASK FORCE IV – OPERATIONAL PROCEDURES

(*Background brief 2, Documents 8-10*)

10:15 to 11:15
- Article of association for SeaBOS [redacted]
- SeaBOS Director – Draft advertisement [redacted]
- Funding mechanism [redacted]
- Discussion
- Decision on SeaBOS Director advertisement

SESSION 7: MONITORING OF PROGRESS

(*Document 11*)

11:15 to 12:30
- Monitoring strategy [redacted]
- Interviews with SeaBOS members [redacted]
- Internal monitoring [redacted]
- External monitoring [redacted]
- Discussion
- Towards September for Task Force IV – summary of next steps [redacted]

SESSION 8: OTHER ACTIVITIES AND NEXT STEPS

13:30 to 14:30	The largest actors in Chinese seafood, draft report [redacted]
	Any other business
14:30 to 15:00	Concluding remarks

List of documents

Document 1 (Background brief 1): Voluntary environmental programs
Document 2: IUU fishing
Document 3: Inventory of transparency and traceability initiatives in the seafood industry
Document 4: Assessing transparency in sustainability reporting by SeaBOS members
Document 5: Advancing transparency through materiality analysis
Document 6: Proposed placeholder document
Document 7 (Background brief 2): Slavery in marine fisheries
Document 8: Proposed Articles of Association for SeaBOS
Document 9: Proposed advertisement for a SeaBOS director
Document 10: Proposed funding mechanism for SeaBOS
Document 11: Proposed Monitoring strategy for SeaBOS

THE KARUIZAWA DIALOGUE

Seafood Business for Ocean Stewardship

FROM COMMITMENTS TO ACTION

Monday, September 3rd

SESSION 1: SETTING THE SCENE

10:30 to 11:00 Opening statement [redacted]

What SeaBOS has set out to achieve – vision and mission [redacted]

How SeaBOS is advancing the frontiers of science [redacted]

Overview of the agenda and workflow [redacted]

SESSION 2: SEABOS ACTIVITIES

11:00 to 11:15 Overview of SeaBOS activities and progress to date [redacted]

SESSION 3: TASK FORCES – STATUS, ADVANCING PROGRESS AND EXECUTIVE DECISIONS

(Background briefs 1, 2, Document 1)

11:15 to 16:15 Task Force I – Reducing IUU fishing and eliminating modern slavery

- Status of Task Force and SeaBOS commitments [redacted]
- Identified problems and potential voluntary actions [redacted]
- Piloting voluntary actions through a Proof of Concept [redacted]
- Discussion: Process for agreement on SeaBOS voluntary actions to address IUU fishing and modern slavery

Task Force II – Advancing traceability and transparency

- Status of Task Force and SeaBOS commitments [redacted]
- Update on Global Dialogue on Seafood Traceability (GDST) [redacted]
- Discussion: Agreement on strategy for advancing traceability and transparency

Task Force III – Advancing work with governments and the regulatory context

- Status of the Task Force and SeaBOS commitments [redacted]
- United Nations Global Compact Ocean Action Platform [redacted]
- Draft voluntary actions to reduce disease in aquaculture [redacted]
- Discussion: Agreement on process to identify policy priorities

Task Force V – Innovation

- Progress by Marine Harvest on reducing marine plastics [redacted]
- Discussion: Approach for reducing marine plastics in aquaculture, feeds and capture fisheries

Summary of day 1 [redacted]

Tuesday 15th May

SESSION 4: TASK FORCE IV – GOVERNANCE AND MONITORING OF SEABOS

(Documents 2, 3, 4)

09:00 to 11:00 The need for an articles of association, funding mechanisms and a new Director [redacted].

Discussion: Agreement on SeaBOS Articles of Association [redacted]

Discussion: Agreement on SeaBOS funding mechanism [redacted]

Discussion: Agreement on SeaBOS director [redacted]

Discussion: Agreement on process to identify Key Performance Indicators (KPIs) and associated deadlines [redacted]

SESSION 5: TASK FORCE IV (CONT'D) - STRATEGY, ORGANIZATION AND FUTURE VISION OF SEABOS

(*Background brief 3*)

11:00 to 12:30 Perception of progress to date – process, information flow, leadership [redacted]

Discussion: Alignment, progress and expectations from members

Discussion: How to improve SeaBOS operations

SESSION 6: JAPANESE EFFORTS IN SEABOS AND POSSIBLE CHINESE ENGAGEMENT

13:30 to 14:30 Maruha Nichiro Corporation

Nippon Suisan Kaisha

Zhejiang Ocean Family Co. (observer from China)

SESSION 7: NEXT STEPS AND FINAL REMARKS

14:30 to 15:30 Suggested next steps [redacted]

Agreement on next steps

End of meeting [redacted]

List of briefs and documents

Background Brief 1: Supply Chain Questionnaire Survey: IUU Fishing and Modern Slavery
Background Brief 2: Sustainability reporting by SeaBOS members
Background Brief 3: What do members have to say about SeaBOS? An analysis of interviews conducted in 2018
Document 1: Draft voluntary actions for IUU fishing and modern slavery
Document 2: Articles of association
Document 3: Funding mechanism and budget
Document 4: Draft Key Performance Indicators (KPIs)

Seafood Business for Ocean Stewardship (SeaBOS)

OUR PLEDGE FOR COMBATTING IUU FISHING RELATED TO JAPAN

PREAMBLE

1. **Welcoming the amendment to the Fishery Act of Japan:**
   As members of the Seafood Business for Ocean Stewardship initiative (SeaBOS), our companies welcome the recent amendment to the Fishery Act of Japan that was adopted on 8th December 2018. By contributing in an important way to developing and improving the scientific management of Japan's fishery resources, the Fishery Act amendment will help ensure the sustainability and profitability of the Japanese seafood industry for years to come. We support the appropriate implementation and enforcement of the amended law.

2. **Our aspirations for future policy developments in Japan:**
   As major seafood companies, we recognize and appreciate the role that Japan plays as one of the largest and most important fish consuming and producing nations. By modernizing the scientific basis for management of Japan's fisheries and aquaculture, the new Fishery Act amendment is an important step forward. However, efforts to ensure the sustainability and profitability of Japan's seafood industry continue to face negative impacts from illegal, unreported, and unregulated (IUU) fishing, which persists in many fisheries around the world including in the EEZ of Japan. As long as the products of IUU fishing are able to enter markets in Japan and elsewhere, responsible seafood products will face unfair competition. For this reason, we strongly support the future development of systems that help eliminate IUU fishing from seafood supply chains.

OUR PLEDGE

3. **Elimination of any IUU fishing and modern slavery from our supply chain:**
   In our capacity as an initiative of ten of the largest seafood companies in the world, we have pledged to make it a priority to ensure that IUU fishing and modern slavery are eliminated from our supply chains. This work has only recently begun, but we are already making progress in developing corporate strategies, policies and practices, that will substantially reduce the risks of such problems. One important mechanism is through improved traceability, including through our collaboration with the Global Dialogue on Seafood Traceability (GDST).

4. **Sharing our progress and experiences in eliminating IUU fishing:**
   We pledge to share our progress and experiences in eliminating IUU fishing and modern slavery, and in improving traceability. We welcome an opportunity to collaborate with the Japanese government and production countries' governments in the move towards such goals.

Chairman Shigeru Ito,
on behalf of all SeaBOS members

THE BERGEN DIALOGUE

PROGRAM

Seafood Business for Ocean Stewardship

2ND WORKING MEETING

Monday, May 13th

19:00 Dinner

Tuesday, May 14th

SESSION 1: OPENING AND REFLECTIONS

08:30 to 08:50 *Opening remarks*

- Opening statement [redacted]
- Welcome to Bergen and Mowi: [redacted]
- From words to action – the world is watching: [redacted]
- Overview of the agenda and work flow: [redacted]

08:50 to 09:30 *How did we get to where we are now?*

- Keystone actors – can and will they make a difference?: [redacted]
- What is the value of SeaBOS – insights from members: [redacted]
- Reflections – how is SeaBOS adding value and what are main challenges for advancement? Tour around the table

SESSION 2: PROBLEMS OF, AND APPROACHES TO, IUU FISHING AND MODERN SLAVERY

(Background document 1, 2, 3)

09.30 to 10.35 *Defining the problems*

- What problems are we trying to solve, and how? [redacted]
- A global risk map (IUU fishing/modern slavery) – identified priorities: [redacted]
- Discussion – what are the problems, and where? What SeaBOS focus?
- Questions for clarification

10.35 to 11.20 *Identifying success factors and priorities*

- Example of success 1: Addressing modern slavery in South East Asia: [redacted]
- Example of success 2: Improving transparency in Peru: [redacted]
- Identification of voluntary actions and ways to address them: [redacted]
- Discussion

11.20 to 11.40 Coffee

SESSION 3: ACHIEVING A STEP CHANGE - INCREASE IN TRACEABILITY

(Background document 2, 3)

11.40 to 11.50 *Proof of concept: Piloting the risk map*

- Identification of company to volunteer application of risk map

11.50 to 12.45 *Novel technologies and industry approaches to traceability*

- Proof-of-concept: rationale for, and results from, blockchain pilot: [redacted]
- Global Dialogue on Seafood Traceability – key data elements: [redacted]
- Discussion of applicability (blockchain & GDST) and suggested ways forward

12.45 to13.45 Lunch

SESSION 4: ADDRESSING THE OCEAN PLASTIC ISSUE

(Background document 4)

13.45 to 14.30 *State of knowledge and examples from leaders*

- State of scientific knowledge and mapping of promising initiatives: [redacted]
- Member advances 1: Addressing plastics in aquaculture: [redacted]
- Member advances 2: Addressing plastics in fisheries: [redacted]
- Member advances 3: Clean Ocean Material Alliance: [redacted]

14.30 to 14.50 *Identifying a SeaBOS strategy on plastics*

- Key components in SeaBOS strategy – house in order and global approach: [redacted]
- Discussion and goals for September

14.50 to 15.00 Coffee

SESSION 5: ANTIBIOTICS – A NEW PRIORITY FOR SEABOS

15.00 to 15.45

- Scientific update – progress, problems and potential: [redacted]
- Example of success: Reducing antibiotics in Norway: [redacted]
- Example of success: Benefits of transparency in antibiotics use: [redacted]
- Discussion of way forward and goals for September
- End session

16.00 Boat trip from Dræggekaien (right outside Radisson Blu Royal Bergen)

17.00 to18.00 Presentation and tour of Haverøy seawater farm

18.00 to 19.00 Transport by boat from Haverøy to Cornelius restaurant

TBD Transport by boat back to Dræggekaien (30 mins)

Wednesday, May 15th

SESSION 6: SEABOS PROGRESS IN ADVANCING COMMITMENTS
(Background document 5)

08.30 to 09.30

- A summary of SeaBOS progress – as reported in 2018: [redacted]
- The Seafood Stewardship Index (SSI) and SeaBOS member status: [redacted]
- Suggested ways to further advance transparency: [redacted]
- Discussion in groups – how to accelerate progress in SeaBOS?
- Reporting back

SESSION 7: MONITORING AND EVALUATION OF SEABOS
(*Background brief 6)*

09.30 to 10.30

- How to monitor the keystone actor experiment: [redacted]
- Example of successes: Setting the right incentives within companies: [redacted]
- Ensuring advances of commitments through KPIs: [redacted]
- Discussion: Timeline for developments of KPIs, joint reporting and new members

10.30-10.45 Coffee

SESSION 8: KNOWLEDGE GAPS AND CHALLENGES OF COOPERATION

10.45 to 11.45

- Knowledge gaps and challenges as perceived by scientists: [redacted]
- Knowledge gaps and challenges as perceived by SeaBOS members (tour around table)
- Group discussion: How to eliminate knowledge gaps and challenges of making progress?
- Reporting back and summary

SESSION 9: POLICY AND PRACTICE - TOWARDS THE 4TH KEYSTONE DIALOGUE IN THAILAND

11.45 to 12.45

- Japanese policy developments and SeaBOS collaboration: [redacted]
- Ocean policy developments – an update, suggested focus and voluntary actions: [redacted]
- Roles and responsibilities: SeaBOS members, secretariat, SRC: [redacted]
- Identified action items, leadership and deadlines: [redacted]
- Are we making progress towards ocean stewardship: [redacted]
- Round of final reflections

12.45 Lunch

List of background documents

Background document 1: An updated risk map
Background document 2: Task Force I deliverables
Background document 3: Moving ahead with SeaBOS Commitments: Insights from company visits
Background document 4: Ocean Plastic Pollution – Trends, Impacts, and Action
Background document 5: Monitoring industry impact of the SeaBOS Initiative
Background document 6: KPI approach: From Bergen to Thailand

THE
PHUKET
DIALOGUE

PROGRAM
September 2019

Seafood Business for Ocean Stewardship

GLOBAL CONNECTIVITY: CONSOLIDATING AND ACCELERATING CHANGE

Sunday, September 1st

18:00 **Welcome Dinner** – Hosted by Thai Union Group

Venue: Layan Beach, Anantara Layan Phuket Resort

Monday, September 2nd

06:30 to 10:30 Breakfast: Dee Plee Restaurant, next to the lobby

SESSION 1: SETTING THE SCENE

Venue: Layan Chapel

09:00 to 09:30 **1.1 Opening**

- Welcome from Chairman Ito
- Introductions, meeting arrangements, and apologies for absence
- Final outcomes from Karuizawa Dialogue
- *Background brief 1 – SeaBOS: a brief history*
- SeaBOS formalised [redacted]

SESSION 2: LEADERS FOR OCEAN STEWARDSHIP

09:30 to 10:45 **2.1 Member aspirations for SeaBOS**

- Interventions on aspirations for SeaBOS from each Keystone Actor
  (Cermaq, Dongwon, Cargill, Maruha Nichiro, Nutreco, CP Foods, Nippon Suisan, Thai Union, Kyokuyo, Mowi)
- Intervention from Stockholm Resilience Centre [redacted]
- Joint reflection

10.45 to 11.15 **Coffee break**

SESSION 3: TASK FORCES - PART I

11.15 to 12.00 **2.2 Transparency**

- *Background Brief 2: SeaBOS commitments and current alignment with material issues across SeaBOS members*
- Brief introductory presentation [redacted]
- Specific initiatives and recommendations
  - o Global Reporting Initiative [redacted]
    - - Use by SeaBOS companies;
  - o Seafood Stewardship Index
    - - Media statement and future engagement from SeaBOS
  - o Global Compact Action Platform for Sustainable Ocean Business [redacted]
- Strategy discussions
  - o Where do we want transparency to be by October 2020 for SeaBOS members?
  - o How are we going to achieve that?

12.00 to 13.00 **2.3 Plastics in the Ocean**

- *Background brief 3: Ocean Plastic Pollution*
- Brief introductory presentation [redacted]
- Presentations and leadership updates
  - o Mowi – examples and targets for plastics
  - o CP Foods – plastics collection by fishers
  - o Thai Union – Global Ghost Gear Initiative (GGGI)
  - o Maruha Nichiro –Clean Ocean Material Alliance (CLOMA)
- Strategy discussions
  - o Where do we want to get to, by October 2020, in relation to reduction of plastics in the supply chain and/or pollution?
  - o How are we going to achieve that?

13.00 to 14.00 **Lunch**: Anantara hotel, Venue: Dee Plee Restaurant

14.00 to 15.00 **2.4 Traceability**

- Presentations:
  - o Global Dialogue on Seafood Traceability update [redacted]
  - o Getting Data on Fish Meal and Fish Oil (IFFO) update [redacted]
  - o Pilot study on traceability in Japan [redacted]
  - o Pilot study on traceability in Australia [redacted]
  - o Pilot study on blockchain technology as a means of enhancing traceability, and outcomes of task force considerations and recommendations [redacted]
- Strategy discussions
  - o Where do we want to get to by October 2020, in relation to traceability issues?
  - o How are we going to achieve it?

15.00 to 15.30 **Coffee break**

15.30 to 16.20 **2.5 Anti-Microbial Resistance (AMR)**

- *Background brief 4: Antibiotics in aquaculture*
- Presentation – Why is this an important topic [redacted]
- Strategy discussions
  - o Where does SeaBOS want to be by October 2020?
  - o How are we able to make this happen?

16:20 to 16:40 **2.6 Reducing IUU fishing and eliminating forced labour**

- *Background brief 5: Risk map results and principles for use*
- Presentation summary [redacted]
- Strategy discussions part 1 (to be continued September 3rd)
  - o Where do we want to be, by when?

16:40 to 17:30 **2.7 Working with governments to improve regulations**

- Presentation from invited guest, [redacted]
  - o *Thailand Fisheries Governance and Transformation - the impacts of EU 'Yellow Card' on Thailand fisheries, and lessons for SeaBOS to help governments to improve regulations* [redacted]
- Update on SeaBOS policy efforts [redacted]
- Update on High Level Panel for a Sustainable Ocean Economy [redacted]
- General discussions on lessons, and future approaches from SeaBOS to assist governments with improved regulations

17.30 **End of meeting for the day**

18.15 **Bus to depart from Reception for dinner**

19.00 **Dinner** – Hosted by Charoen Pokphand Foods

Venue: Black Ginger Restaurant

Tuesday, September 3rd

06:30 to 10:30 Breakfast: Dee Plee Restaurant

SESSION 3: TASK FORCES - PART II

09.00 to 09.05 **Summary from previous day**

09.05 to 10.45 **3.1 Reducing IUU fishing and eliminating forced labour (continued)**

- *Background brief 6: Task Force 1: progress update*
- Brief introductory presentation [redacted]
- Presentation
  - o How to deal with results of traceability showing unsustainable stocks, and the links to risk mapping (Toshiya Yabuki: update on actions to progress sustainable supply by 2030)

continued on next page

- o Identifying where the people are; doing labour audits and what that means for the risk map [redacted]
- o Raw materials and country sources [redacted]
- Discussions and agreement on using the Risk Map and Voluntary Actions

10.45 to 11.15 **Coffee break**

SESSION 4: PERFORMANCE MANAGEMENT

11.15 to 12.00 **4.1 Monitoring of SeaBOS**

- *Background brief 7 – Key Performance Indicators: survey results and a way forward*
- Presentation and outline [redacted]
- Where do we want to be by October 2020 with KPIs?
- Agree on recommendations and/or process for finalising KPIs

12.00 to 13.00 **Lunch**: Anantara hotel, Venue: Dee Plee Restaurant

SESSION 5: SEABOS GOVERNANCE

13.00 to 14.00 **5.1 Governance of SeaBOS**

- Presentation: Roles and responsibilities - SRC collaborative deed and future funding (Henrik Österblom)
- Discussions and approvals
  - o Legal and administrative requirements
  - o Next SeaBOS meetings; venues and dates
  - o Communication and connections
    - - SeaBOS logo, website, info-graphics for presentations
    - - SeaBOS participation at events in 2020
    - - Scientific horizon-scanning [redacted]
    - - Promoting benefits of seafood towards reducing climate change [redacted]
  - o Budget 2020
  - o New member invitations for 2020
  - o Items for Information
    - - Accounting, auditing and legal appointments
    - - Insurance
    - - Secretariat establishment
  - o Other Business
  - o Closing remarks [redacted]

14.00 **Meeting closes**

List of background documents

Background document 1: Seafood Business for Ocean Stewardship: a brief history
Background document 2: SeaBOS commitments and current alignment with material issues across SeaBOS members
Background document 3: Ocean plastic pollution
Background document 4: Antibiotics in aquaculture
Background document 5: Risk map approach and principles for use
Background document 6: Task Force I: Progress update
Background document 7: Key Performance Indicators: Survey results and a way forward and propositions

THE STOCKHOLM DIALOGUE

PROGRAM
May 2020

Seafood Business for Ocean Stewardship

3RD WORKING MEETING

A virtual meeting due to the COVID-19 pandemic

Time (GMT+2)	Monday, May 11
**08:30 – 09:45**	**OPENING REMARKS, SCIENCE SUMMARY AND BIG PICTURE** (SESSION 1)
08:30 – 08:45	Opening statement [redacted]
08:45 – 08:55	Welcome/Overview of agenda [redacted]
08:55 – 09:00	Anti-trust legislation and considerations [redacted] **1.1**
09:00 – 09:20	SeaBOS science summary and grand challenges [redacted] **1.2**
09:20 – 09:45	Discussions - what can SeaBOS deliver that is more than individual companies?
09:45 – 10:00	*Break*
**10:00 – 12:30**	**TASK FORCE I (IUU, ENDANGERED SPECIES, AND MODERN SLAVERY)** (SESSION 2)
10:00 – 10:25	IUU and labour abuse risk mapping [redacted]) **2.1**
10:25 – 10:40	Discussion and questions
10:40 – 10:50	Moving forward with human and labour rights issues [redacted] **2.2**
10:50 – 11:00	Survey of Fishery improvement programmes ([redacted]) **2.3**
11:00 – 11:10	Voluntary Actions outcomes ([redacted]) **2.4**
11:10 – 11:20	Next steps on IUU and modern slavery ([redacted]) **2.5 (rev), 2.5(a), 2.5(b)**
11:20 – 11:25	*Break*
11:25 – 12:30	Discussion in break out groups, identification of KPIs ([redacted]) **7**

	Tuesday, May 12
**08:30 – 10:30**	**TASK FORCE III (WORKING WITH GOVERNMENTS) AND TASK FORCE IV (TRANSPARENCY AND GOVERNANCE)** (SESSION 3)
08:30 – 08:45	AMR survey update and future options ([redacted]) **3.1**
08:45 – 09:30	Discussions; recommended approaches, KPIs ([redacted]) **7**
09:30 – 10:30	Governance (Legal, audit, comms, admin, HLP, UNGC, KPIs) ([redacted]) **3.2, 3.2.1, 3.2.2, 3.2.3, 3.3, 3.3 (a), 3.4, 3.4(a), 3.4(b), 7**
10:30 – 10:45	*Break*

10:45 – 12:30 **TASK FORCE II (TRACEABILITY)** (Session 4)

10:45 – 11:30 Member presentations: ([redacted]) **4.1**
11:30 – 11:45 Presentation from GDST (Rapid Internal Assessment update, progress) **4.2, 4.2(a)**
11:45 – 11:55 Traceability ([redacted]) **4.3**
11:55 – 12:30 Discussions, recommendations, KPIs ([redacted]) **7**

Wednesday, May 13

08:30 – 10:30 **TASK FORCE V (PLASTICS)** (SESSION 5)

08:30 – 08:45 Plastics questionnaire responses and summary of initiatives ([redacted]) **5.1**
08:45 – 09:15 Member plastics initiatives presentations: ([redacted]) **5.2**
09:15 – 09:30 GGGI progress and opportunities ([redacted]) **5.3 (rev), 5.3.1, 5.3.2, 5.3.2(a)**
09:30 – 10:30 Discussions, recommendations, and KPIs ([redacted]) **7**

10:30 – 10:45 *Break*

10:45 – 12:30 **TASK FORCE VI (CLIMATE RESILIENCE)** (Session 6)

10:45 – 11:00 Climate resilience brief, and climate resilience survey results ([redacted]) **6.1(a), 6.1(b)**
11:00 – 11:15 Best practice ocean activity reporting by others ([redacted]) **6.2**
11:15 – 12:30 Discussions, Breakout groups, Recommendations and KPIs ([redacted]) **7**

Thursday, May 14

09:15 – 11:30 **SUMMARY SESSION**

09:15 – 10:15 Summary discussions, identification of next steps, assigning responsibilities and time-lines
10:30 – 11:30 Report to Her Royal Highness Crown Princess Victoria of Sweden

List of background documents

Science updates

1.2 Corporate Biosphere Stewardship in the Anthropocene- exploring the frontiers of science
2.1 Eyes in the Sky and Eyes on the Ground: Developing risk maps for IUU and labour abuse
2.2 Moving forward with human and labour rights issues
2.3 Fishery Improvement Projects (FIPs): Global overview and current SeaBOS activities
2.4(a) Voluntary Actions update
2.4(b) Voluntary Actions survey summary results for all
5.1 Plastics – Aggregated Survey Results
6.1(a) Addressing Climate Change Impacts on Fisheries and Aquaculture
6.1(b) Climate Resilience – Aggregated Survey Results
6.2 Survey of climate strategy and reporting: work in progress update

Agenda papers

1.1 A reminder on anti-competition requirements
2.5(rev) Next steps on IUU, endangered species and modern slavery
2.5(a) Meta-collaboration proposal on PSMA and GDST
2.5(b) Draft industry statement on GDST and PSMA
3.2 Governance of SeaBOS
3.2.1 SRC and SeaBOS operational MOU
3.2.2 SRC and SeaBOS research MOU
3.2.3 Draft principles for SeaBOS engagement
3.3 UN Global Compact and High Level Panel
3.3(a) UN Global Compact press release
3.4 SeaBOS communications informal network
3.4(a) SeaBOS informal communications network introductory letter
3.4(b) GGGI foreword in their 2019 annual report
4.2(a) GDST-SeaBOS Roadmap proposal
4.3 Traceability
5.3(rev) Reducing ocean plastics – a vision for SeaBOS
5.3.1 GGGI Corporate benefits 2020
5.3.1(a) SeaBOS Report foreword in GGGI 2019 annual report
5.3.2 Ocean Conservancy opportunities
5.3.2(a) Trash free seas program
7 Key Performance Indicators for SeaBOS

Resilience through Ocean Stewardship – Leadership, Actions, and Opportunities

Monday, October 5th

Time (UTC+2)

08:00 – 08:10 **SESSION 1: ADMINISTRATION** (10 MINUTES)

1.0 Meeting arrangements, approval of agenda, and apologies ([redacted])
1.1 Reminder on anti-trust legislation and requirements ([redacted])
1.2 Approved final minutes from Phuket Dialogue and May working meeting ([redacted])

08:10 – 09:35 **SESSION 2: THE BIOSPHERE CRISIS** (85 MINUTES)

- **Formal Welcome and opening of meeting** ([redacted]) (15 minutes)
- **Working with SeaBOS** ([redacted]) (15 minutes)
- **The Biosphere Crisis** ([redacted]) (20 minutes)
- **Our Shared Vision – SeaBOS commitments** ([redacted]) (15 minutes)
- **How are we going?** – Are there lessons from the impacts of the covid crisis which can apply to short- and longer-term resolution of the biosphere crisis? Comments from each CEO on their perception of progress in SeaBOS towards implementing biosphere stewardship to help avert the biosphere crisis. (20 minutes)
    - [redacted]
    - [redacted]
    - [redacted]
    - [redacted]
    - [redacted]
    - [redacted]
    - [redacted]
    - [redacted]
    - [redacted]
    - [redacted]
    - [redacted]
    - [redacted]
    - [redacted]

09:35 – 09:40 *Short break (please do not leave meeting, just mute audio and stop video)*

09:40 – 11:30 **SESSION 3: LEADERSHIP, ACTIONS, AND OPPORTUNITIES**

09:40 – 10:30 (50 minutes)

Task Force I (IUU and modern slavery)

Presentations of scientific background papers ([redacted])

1. Eyes in the Sky and Eyes on the Ground: Developing Risk Maps for IUU and Labor Abuse ([redacted])
2. Defining 'Modern Slavery' and Identifying Corporate Responsibility ([redacted])
3. Reporting and Benchmarking of Human and Labour Rights Reporting ([redacted])
4. Fishery Improvement Projects: Introduction and Global Overview ([redacted])

10:30 – 11:00 (30 minutes)

3.1 Recommendations to CEOs ([redacted]) (20 minutes)

- Illegal, Unreported and Unregulated fishing
- Modern Slavery
- Endangered Species
- Meta coalition proposal

Key decisions by CEOs: Agreement on time bound goals for addressing IUU and modern slavery

Task Force II (Traceability)

3.2 Recommendations to CEOs ([redacted]) (10 minutes)

- Traceability update

Key decisions by CEOs: Agreement to include traceability into Task Force I, and continue collaboration with GDST

11:00 – 11:05 *Short break (please do not leave meeting, just mute audio and stop video)*

11:05 – 11:30 **Our Leadership, Actions and Opportunities continued**

Task Force III (Working with Governments)

3.3 Recommendations to CEOs ([redacted]) (25 minutes)

- Anti-microbial resistance
- Working with governments

Key decisions by CEOs: Agreement on time bound goals for addressing AMR, and working with governments

Close of Day 1 (11:30)

Tuesday, October 6th

08:00 – 10:40 **SESSION 3: LEADERSHIP, ACTIONS, AND OPPORTUNITIES** (CONTINUED)

08:00 – 08:05 **Summary from previous day (Secretariat)**

08:05 – 09:35 **Our Leadership, Actions and Opportunities**

08:05 – 08:30 (25 minutes)

Task Force III (Working with governments)

High Level Panel and other initiatives ([redacted])

Scientific presentations on:

- Summary of Blue Papers ([redacted])
- Blue Foods Assessment update ([redacted])
- Progress on Ocean Stewardship ([redacted])

Discussions and summary actions

08:30 – 09:35 (65 minutes)

Task Force V (Plastics)

3.4 Recommendations to CEOs ([redacted]) (20 minutes)

- 'City to Sea' Plastics program for SeaBOS
    - GGGI
    - Coastal Clean up
    - Science of plastics in seafood
    - Packaging baseline assessments
    - Communicating goals on packaging
    - Collaboration with governments

Key decision by CEOs: Agreement on time bound goals for reducing ocean plastics

Task Force VI (Climate Resilience)

Presentations of scientific background papers ([redacted]) (25 minutes)

5. Addressing Climate Change Impacts on Fisheries and Aquaculture ([redacted])
6. Survey of Corporate Climate Change Strategies ([redacted])

3.5 Recommendations to CEOs ([redacted]) (20 minutes)

- Climate change impacts on seafood production
- Reduction of emissions
- Seafood as a solution to climate change
- Regulatory flexibility and support

Key decisions by CEOs: Agreement on time bound goals for reducing carbon emissions and setting science-based targets.

09:35 – 09:45 *Short break (please do not leave meeting, just mute audio and stop video)*

09:45 – 10:40 **Our Leadership, Actions and Opportunities continued**

09:45 – 10:00 (15 minutes)

Task Force IV (Governance and Transparency)

3.6 Recommendations to CEOs ([redacted])

Policy and administration

- Draft principles for SeaBOS engagement
- Research protocol for SeaBOS
- SeaBOS Secretariat and SRC Operational MOU
- Communications Task Force establishment

Key decisions by CEOs: Agreement to new policies and principles, and to set up a Task Force on Communications

10:00 – 10:40 (40 minutes)

We will limit this next section of the meeting to CEOs, [redacted] *Could everyone else please depart the zoom meeting, and re-join at the 10:40 break, thank you.*

3.7 SeaBOS Association Director elections, and legal requirements ([redacted])

- Election of Chair
- Election of Vice Chair
- Election of Board
- Election of New Member Selection Committee
- Other Formal requirements
    - Next meeting dates (Sunday evening 3 October until end Tuesday 5 October 2021 in Amsterdam; kind offer to host from Cargill/Nutreco)
    - Managing Director review of arrangements
    - Audit and annual report of the SeaBOS Fundraising Foundation
    - Insurance recommendations to the SeaBOS Fundraising Foundation
    - Review of relevant SeaBOS Association policies and guidelines
        - Rules of procedure for the Board of Management of SeaBOS Assosciation
        - Rules of procedure for the Board of Directors of SeaBOS Fundraising Foundation
        - Travel and expenses
        - Workplace Ethics, Health and Safety
        - Guidelines on Trade Competition
    - Budget recommendations to the SeaBOS Fundraising Foundation
        - USA Foundation support
    - New members

10:40 – 10:45 *Short break (please do not leave meeting, just mute audio and stop video)*

10:45 – 11:30 SESSION 4: THE VISION (45 MINUTES)

Outcome ([redacted])

- CEO views of meeting and 'next steps' – is our original vision correct, do our commitments require any changes? (2 minutes each)
    - [redacted]
    - [redacted]
    - [redacted]
    - [redacted]
    - [redacted]
    - [redacted]
    - [redacted]
    - [redacted]
    - [redacted]
    - [redacted]
    - [redacted]
    - [redacted]
    - [redacted]
- Closing remarks by [redacted]
- Closing remarks by [redacted]

Meeting closes at 11:30

List of background documents

Scientific background papers

1. Eyes in the Sky and Eyes on the Ground: Developing risk maps for IUU and labor abuse
2. Defining 'Modern Slavery' and Identifying Corporate Responsibility
3. Reporting and benchmarking of human and labour rights reporting
4. Fishery Improvement Projects (FIPs): Introduction and Global Overview
5. Addressing Climate Change Impacts on Fisheries and Aquaculture
6. Survey of corporate climate change strategies

Agenda papers

1.1 Anti-competition requirements
1.2.1 Final minutes from Phuket dialogue (2019)
1.2.2 Action items from Phuket dialogue (2019)
1.2.3 Final outcomes of Stockholm working meeting 11 – 14 May 2020
1.2.4 Summary outcomes for CEOs from May working meeting, and Task Force membership
3.1.1 Task Force I recommendations
3.1.2 Eliminating forced, bonded and child labour
3.2 Task Force II recommendations
3.3.1 Task Force III recommendations
3.4.1 Task Force V recommendations
3.4.2 Concept outline for GGGI and International Coastal Cleanup
3.5 Task Force VI recommendations
3.6 Task Force IV recommendations
3.6.1 Draft principles for SeaBOS Engagement
3.6.2 Research protocol for SeaBOS
3.6.3 SeaBOS Secretariat and SRC Operational MOU
3.7 SeaBOS Association Board of Management elections, and legal requirements
3.7.1 SeaBOS Association finalised statutes 19 December 2019
3.7.1.1 Rules of procedure for the board of management of the SeaBOS Association
3.7.2 Rules of procedures for the Board of Directors SeaBOS Fundraising Foundation
3.7.2.1 Unofficial compilation of statutes for SeaBOS Fundraising Foundation
3.7.3 Annual report and audit 2019 for SeaBOS Fundraising Foundation
3.7.4 Policy on travel and expenses for SeaBOS Foundation Board
3.7.5 Policy on workplace ethics, health and safety
3.7.6 Policy on anti-competition requirements
3.7.7 D&O insurance
3.7.8 General Liability and Product Liability Package insurance

Goals for 2021

- End IUU fishing and forced, bonded and child labour in their own operations & implement measures to address those issues in their supply chains
- Collaborate with the Global Ghost Gear Initiative to solve the problem of lost and abandoned fishing gear; and clean up plastic pollution
- Agree on a strategy for reducing impacts on endangered species and the use of antibiotics
- Set CO_2 emissions reduction goals and reporting approaches from each company

SeaBOS

Tool kit in support of SeaBOS commitments and goals (Task Force I)

Introduction

At the October 2020 meeting SeaBOS companies agreed to:

1. Have no IUU fishing products or forced/bonded/child labour in their own seafood operations by October 2021.
2. Put science-based measures in place that, when combined, substantially reduce the risk of IUU fishery products or forced/bonded/child labour in their supply chains.
3. Act swiftly and transparently on any evidence that IUU fishing and forced/bonded/child labour activities exist within their operations and supply chains, in recognition that these activities are endemic within the global seafood industry and require continuous vigilance by all actors.

Taking these challenges into account, SeaBOS members are convinced that the science-based measures that will be put in place, along with continued policy engagement, will support the elimination of both IUU fishing and forced/bonded/child labour in their supply chains. They will report in October 2022 and October 2025 on progress towards meeting their goals.

These commitments represent a substantial challenge for the complex operations of SeaBOS members and there are many possible ways to achieve the outcomes sought. This document, therefore, presents a 'tool kit' of actions that are likely to be helpful, along with techniques that have been used by SeaBOS members (including those relating to traceability) to address the challenges of IUU and labour issues in their own operations, and throughout their supply chains. As experience grows, additional tools will be added.

The recommendations and suggested tools to prevent the existence of IUU fishing products or forced/bonded/child labour have been carefully considered, but are nonetheless not to be understood as prescriptive. Each individual member company undertaking preventative measures must, at each time, evaluate the considered measure based on applicable and relevant conditions and also legally review any measures, including from a fair trade and anti-trust perspective.

1. Governance of own operations and supply chains

Supply chain management requires that policies are put in place; that there is an internal knowledge about the content and sustainability status of the supply chain; that due diligence is used to understand supply chain risks; and that companies actively engage with suppliers throughout their supply chains. Mechanisms that have been shown to be effective and are used by a number of SeaBOS members already, include:

1.1 Internal company policy documents

- Develop company policy documents (e.g. responsible sourcing policies) for raw materials caught or sourced that highlight the urgency of addressing, reducing and eliminating IUU fishing.
- Develop company policy documents (e.g. code of conduct) for addressing the issue of eliminating forced, bonded and child labour.
- These company policy documents should be publicly available (where not commercially confidential) and be used for communication and training purposes.
- Communication associated with company policy documents should include protocols for auditing compliance and remediating issues found.

1.2 Company policy documents for Tier 1 suppliers

- Ensure that Tier 1 raw materials suppliers demonstrate acceptance of company policy documents with respect to raw materials.
- Ensure that Tier 1 suppliers demonstrate acceptance of company policy documents with respect to labour standards.

Note: Learning can be derived from members who have already made these policy documents public.*

1.3 Mapping own operations and Tier 1 in the supply chain

Companies should map their own operations and Tier 1 in the supply chain by applying materiality analysis (i.e. capturing the most impactful aspects of their operations).

- Make an inventory of your portfolio of marine resources, the status of the resources used, and ways to improve traceability and sustainability.
- Map workers and operational sites in your own operations.
- Progressively develop greater insights of the status of raw materials from Tier 1 suppliers and where workers sit in your supply chain.

Note: Learning can be derived from members who have already mapped their marine resources.†

1.4 Science-based due diligence of risk and decision making

- Use a scientific assessment of risk, your own knowledge and other sources of intelligence to identify areas of activity that may require additional due diligence.
- Using your understanding of areas with higher levels of risk, design and implement auditing processes that will mitigate these risks (taking actions and reporting on findings where appropriate).
- Evaluate the relevance of the schedule of possible voluntary actions (see separate document) for mitigating risks you have identified.

1.5 Engaging in collaboration beyond Tier 1 suppliers

- Identify who are the most significant suppliers on a basis that is suitable to your business (e.g. by volume of raw materials or by financial spend) and informed by risk evaluation, and develop a dialogue with them as to how the whole supply chain might be free from IUU fishing and forced/bonded/child labour.
- Develop knowledge of supply chains taking in your own operations, Tier 1, Tier 2 and Tier 3 suppliers (this may need to be prioritized by species, perceived risk, or value of production).
- Collaborate within SeaBOS to support tackling forced/bonded/child labour issues (see e.g.‡)
- Collaborate with relevant governments and other agencies or initiatives on improving outcomes for IUU fishing and forced/bonded/child labour, and provide information on the effectiveness of these collaborations. Possible means to do this include promoting implementation of the PSMA agreement, transparent vessels registry, active engagement in Fisheries Improvement Projects (FIPs), as well as seeking to unite industry, government, civil society, and relevant organisations to focus actions against IUU fishing.
- Collaborate with other bodies (e.g. certification bodies and civil society) to support the credentials of raw materials and labour used in supply chains.

2. Advancing traceability

Traceability represents an opportunity to better understand the content and dynamics of complex supply chains and is instrumental for identifying data gaps and supply chain risks. Traceability also supports administrative improvements such as stock and inventory control; recall of products; QA processes, and branding initiatives. Additionally, it is perceived that using the IoT digital technology may prove an effective tool to address concerns and distinguish those businesses adopting latest onboard vessel technological tools. Substantial work among policy makers, NGOs and industry, and advances in technologies, are all contributing to developing leadership options. Tools for advancing traceability include:

---

* Cargill – Code of Conduct (available in 28 languages)
https://www.cargill.com/about/supplier-code-of-conduct

- Japanese: https://www.cargill.com/doc/1432110367992/supplier-code-of-conduct-pdf_ja.pdf
- Korean: https://www.cargill.com/doc/1432110368156/supplier-code-of-conduct-pdf_ko.pdf
- Norwegian: https://www.cargill.com/doc/1432110368605/supplier-code-of-conduct-pdf_no.pdf
- Thai: https://www.cargill.com/doc/1432110369378/supplier-code-of-conduct-pdf_th.pdf

Nutreco – Code of Conduct (https://www.nutreco.com/globalassets/nutreco-corporate1/corporate/code-of-conduct/code-of-conduct---english.pdf)

Thai Union – Code of Conduct (https://www.thaiunion.com/en/sustainability/code-of-conduct)

† Nissui supply chain methodology and results (page 28)
https://s3-ap-northeast-1.amazonaws.com/sustainability-cms-nissui-s3/pdf/en/2020_sustainability_full_en.pdf

‡ Alliance 8.7 https://www.alliance87.org/.

- Supporting adoption of Global Dialogue on Seafood Traceability (GDST) standards 1.0 as providing key data elements and interoperability systems for traceability.
- Identifying the Key Data Elements relevant to your operations for traceability.
- Connecting your traceability approach to those of your supply and value chain and encourage use of interoperability systems.
- Piloting of technologies that will support traceability development and providing feedback on their effectiveness.

Note: Experimentation and mutual learning have been spearheaded by Nutreco. Thai Union, Cargill Aqua Nutrition and Austral Fisheries can also support this work.

3. External reporting and accountability

External reporting clarifies objectives to external stakeholders and also represents an important process for increased internal communication and verification of progress made, including how time bound goals are being achieved and how companies are progressively implementing relevant components of this tool kit or using other tools to achieve these outcomes. Timelines for reporting on progress in Task Force I goals are October 2022 and October 2025. Reporting will be within SeaBOS members' own reporting routines while a combined 'SeaBOS' report will also be developed on these timelines.

With scientific support from the **Stockholm Resilience Centre** at Stockholm University, the **Beijer Institute of Ecological Economics** and the **Global Economic Dynamics and the Biosphere program** at the Royal Swedish Academy of Sciences, **University of Birmingham** and the **Stanford Center for Ocean Solutions** and finacial support from the **Walton Family Foundation**, the **David and Lucile Packard Foundation**, and the **Gordon and Betty Moore Foundation**.

SUPPORTING DOCUMENT
DECEMBER 2020

Voluntary procurement actions in support of SeaBOS commitments and goals (Task Force I)

This material could provide a basis for any company to develop its own policies for eliminating IUU fishing and forced/bonded/child labour in seafood operations.

Recognizing that:

- IUU fishing (IUU) and forced/bonded/child labour in wild capture seafood fisheries, fisheries destined for fishmeal in feeds, aquaculture operations and in processing activities associated with these activities are unacceptable;
- Many institutions are making efforts to reduce and eradicate IUU and forced/bonded/child labour from the seafood business, and SeaBOS companies wish to support those efforts;
- Yet IUU and forced/bonded/child labour persist, and are increasingly the topics of acute public scrutiny and governmental regulatory efforts;
- SeaBOS member companies wish to be, and be recognized as, enablers of solutions to these crimes and work in partnership with governments, NGOs and consumers to address these complex and persistent problems. Doing this will be fulfilling SeaBOS commitments to ocean stewardship:
    - SeaBOS companies need to get ahead of imminent regulations and show industry leadership through voluntary actions;
    - SeaBOS companies need to fulfil SeaBOS promises and demonstrate to their customers, consumers, and governments that they are part of the solution;
- SeaBOS and the Keystone actor's initiatives were created to produce transformational change, acting more quickly than governments and thereby complementing existing processes.

Proposed elements for a 'best practice' corporate policy statement

This list of voluntary actions is not prescriptive and will apply in different ways to different companies and along different timelines, depending on operational realities and contexts. It is however a robust list, based on a substantial scientific basis, experiences of individual companies and consultation with diverse stakeholders. These actions combined have the potential to substantially reduce the risk of IUU fishing and forced/bonded/child labour in seafood company operations and their respective supply chains.

1. Promoting good governance

Given the role that poor governance (and corruption) plays in sustaining illegal fishing and forced/bonded/child labour, actions that prevent, identify and punish corrupt practices and/or enhance good governance are fundamental to helping achieve SeaBOS aims. One element of this might be addressing issues raised by the use of flags of non-compliance. Likewise, identifying ports where poorer governance exists may also enable focused action to create locations where good governance is the norm.

Possible tools:
Data to support good governance. Note: this list identifies blockchain as a means to collate these data but other approaches for capturing information securely could also be used.

1.1 Fishing vessel captain must upload to blockchain (or similar) the vessel's entire crew list;

1.2 Each crewmember's passport (photo page) to be scanned and uploaded to blockchain (or similar);

1.3 Each crewmember's scanned facial recognition to be uploaded to blockchain (or similar);

1.4 Each crewmember's executed fishing labor contract to be scanned and uploaded to blockchain (or similar);

1.5 When relevant, each crewmember's government issued official document proving their embarkation was duly and lawfully carried out should be scanned and uploaded to blockchain (or similar);

1.6 All eligible fishing and carrier vessels must obtain and make visible an International Maritime Organization (IMO) number so they can be uniquely identified;

1.7 All vessels must be properly registered in national fishing registries that are publicly maintained and participating in the FAO Global Record of Fishing Vessels;

1.8 No vessels will register under "flags of non-compliance" or conduct activities in ports identified as high risk (using the science-based data platform developed by the secretariat);

1.9 All vessels will scan vessel ownership (including beneficial ownership), registration and home port documentation and upload to blockchain (or similar);

1.10 All vessels must scan all licenses for relevant fishing activities issued by flag and/or coastal states and/or RFMOs and upload to blockchain (or similar);

1.11 No vessels will appear on current "black lists" or their equivalent maintained by RFMOs or national authorities;

1.12 All vessels must make first landing in countries that are Party to, and implementing, the Port State Measures Agreement, or have equally effective port State measures in place, including with regard to vessels flagged to the port state;

1.13 All vessels (and/or port state authorities) will upload to blockchain (or similar) all formal landing documents and authorizations, including results of any port state inspections;

1.14 All vessels (either independent contractor or companied owned) will show demonstration of collaboration and partnership with key ports to support their ability to exercise due diligence in their operations;

1.15 All vessels will undergo verification and status of country of their registration to ascertain risk status regarding the country's commitment to ILO forced labour conventions 29, 98 & 182; the ILO work in fishing convention 188; the Palermo Protocols and; and the country's application of the IMO Ship Identification Number Scheme;

1.16 SeaBOS members will give full consideration to decisions of port states and market states, seeking to avoid sourcing products from vessels subject to landing or import restrictions under national regulations against commerce in IUU or MS products.

2. Electronic monitoring and tracking

Fishing activities that are 'out of sight' may contribute to illegal fishing and forced/bonded/child labour. As a result, any activity that increases the transparency of what is happening at sea may be useful and might include technologies such as cameras on deck, the physical presence of more observers, remote sensing of ship movements and blockchain (or similar) monitoring.

Possible tools:
The following will apply to all fishing and carrier vessels supplying fish products into SeaBOS member company supply chains:

2.1 All vessels will use Internet of Things (IoT) devices (i.e. GPS, AIS, VMS, facial recognition software and species recognition software);

2.2 All vessels will collect their relevant data (i.e. GPS tracks, fishing coordinates, fished volume recorded, species recognition (type and % distribution));

2.3 All relevant data will then be cross-checked compared with port landed data at recipient's station;

2.4 All relevant data will be visualized using "Business Analytics" and user friendly Dashboards;

2.5 All relevant data will be the foundation for "catch-to-plate" principles with demonstrated transparent traceability through to final market;

2.6 All authorized vessels will comply with any flag State, coastal State or RFMO manual reporting arrangements in place in case of a vessel monitoring system unit malfunction or failure and will return to port immediately if the unit continues to malfunction or fail;

2.7 All carrier vessels will carry a secondary/backup vessel monitoring system unit to be used in case of a primary unit malfunction or failure.

3. Risk based transhipment related actions

Transhipment refers to the practice of transferring catch and/or crewmembers while at sea from one fishing vessel to either another fishing vessel, a processing vessel or a cargo vessel. This activity may create more efficient fishing systems but can also increase the possibility that IUU fishing/fish goes undetected. From time to time transhipment is prohibited by nation states and/or regional fisheries management organizations and some companies have proposed transhipment bans for their supply chains.

Possible tools:

3.1 When any transhipment activity is to take place, it will be pre-authorized by relevant RFMOs and governments;

3.2 When any transhipment activity takes place, it will be conducted with either "observer" supervision or deck video recording of activity to be subsequently uploaded on blockchain (or similar);

3.3 All authorized vessels intending to tranship ensure they meet all flag State, coastal State or RFMO requirements for observer carriage and reporting;

3.4 All authorized carrier vessels intending to tranship within a specific RFMO shall provide electronic notification of their entry into those waters to the relevant flag State and RFMO Secretariat to include confirmation of the vessel's compliance with vessel monitoring system reporting requirements;

3.5 All authorized vessels intending to tranship submit electronic pre-notifications and post declarations within required timelines to the relevant flag State, port State, coastal State and RFMO Secretariat for every transhipment that occurs regardless of the location of transhipment.

From time to time, transhipment of crew takes place and has the effect of prolonging time at sea for fishers as well as making the tracing of employment harder to achieve. NGOs working on anti-slavery projects have identified that transhipment of crew is disproportionately associated with slavery.

Possible tools:

3.6 There will be no transhipment of crew in geographic marine areas identified as high risk (through science-based data platform/risk assessment);

3.7 There will be no transhipment of crews from/to vessels flying "flags of non-compliance";

3.8 Before, during and after transhipment of crew, vessels must report to relevant Flag State authorities or Coastal State authorities. Where applicable to Regional Fisheries Management Organizations and governments.

4. Changing recruitment practices

Employment brokers/agents play a valuable role in linking fishers to vessels but can also be the means by which enslavement is initiated. A common practice is for a fisher to pay employment brokers for their services (often pledged from their future earnings) and this may create an enabling environment for debt bondage to emerge.

Possible tools:

4.1 If a company utilizes employment brokers/agents it should demonstrate that it has utilized an 'employer pays principle';

4.2 Whenever possible and relevant, company will avoid employment brokers/agents and use own HR recruiting of vessel crews;

4.3 All vessels (either independent contractor or companied owned) need to comply with Criteria 1.1-1.6 above;

4.4 Verification and status of country where crewmembers are recruited and contracted to ascertain risk status regarding the country's commitment to ILO forced labour conventions 29, 98 & 182; the ILO work in fishing convention 188; and the Palermo Protocols;

4.5 Company must facilitate crewmember feedback through on-line surveys at regular intervals not to exceed twice annually.

5. Developing more robust payments methods

At the heart of forced labour is the failure to make appropriate payments for work or to make payments at all. A less direct method for detecting slavery, therefore, is the tracing of wage payments to ensure that these are of the quantity that one would expect given the vessel and work in question. Moreover, making these payments secure in terms of being paid directly to a bank account that only the individual fisher can access would be further proof that slavery is not present.

Possible tools:

5.1 All vessels (either independent contractor or companied owned) need to comply with Criteria 1.4 above;

5.2 All vessels (either independent contractor or companied owned) need to demonstrate monthly payment deposit into crewmember established bank account, scanned and uploaded to blockchain (or similar). Payment deposit slip intervals should not exceed monthly intervals;

5.3 Where relevant, companies should seek partners for the effective 'banking' of fishers crewmembers (from NGOs and nation states where banking would have to be achieved).

6. Marine raw material sourcing policy

Illegal fishing and forced/bonded/child labour arise in complex supply chains that are often beyond the direct control and influence of the ultimate purchaser of fish. There are cost related arguments as to why this type of economic arrangement is useful but it does, inevitably, create the possibilities for unfree labour. Changing the nature of relationships in the supply chain offers some possibilities for combating fisheries crimes but

only if enacted in conjunction with other mitigation actions. Likewise, designing 'best in class' codes of conduct and assessing compliance with those codes in a robust fashion will provide some protection against undetected issues in supply chains.

Possible tools:

6.1 All SeaBOS member companies will demonstrate supply chain human rights mapping which should calculate the full path of its product's value-chain from harvest to market;

6.2 Company will, whenever possible, give preference to company owned/controlled fishing vessel in supply chain;

6.3 Company will restrict independent contractor fishing vessels to Tier 1 and Tier 2 supply chain;

6.4 All SeaBOS member companies will participate in, and require their supply chains to participate in, digital, full-chain traceability systems that comply with prevailing industry standards (such as those promulgated by the Global Dialogue on Seafood Traceability), and with all applicable national or international legal traceability requirements.

7. Restricting areas of operation

In order to mitigate risks, a partial or full withdrawal of operations from certain regions might be in order. Examples of this include not fishing in areas where the risk of illegal fishing and forced/bonded/child labour slavery is high.

Possible tools:

7.1 No vessel (either independent contractor or company-owned) is allowed to enter prohibited marine sanctuaries, prohibited marine protected areas and other internationally recognized "no-go-zones" as published by FAO. All relevant vessel data will be visualized using "Business Analytics as defined in Criteria 6.4 above;

7.2 Where navigation through restricted areas (7.1) is essential, then pre-advice to the relevant authority of transit should occur, and the vessel must continue to steam at a constant rate (e.g. greater than 5 knots at all times) and via the most direct route through the marine sanctuary (i.e. no stopping unless in case of emergency and again that should require notification to the relevant authority in charge of that marine sanctuary).

With scientific support from the **Stockholm Resilience Centre** at Stockholm University, the **Beijer Institute of Ecological Economics** and the **Global Economic Dynamics and the Biosphere program** at the Royal Swedish Academy of Sciences, **University of Birmingham** and the **Stanford Center for Ocean Solutions** and finacial support from the **Walton Family Foundation**, the **David and Lucile Packard Foundation**, and the **Gordon and Betty Moore Foundation**.

THE 2021 VIRTUAL
WORKING MEETING

PROGRAM
May 2021

Seafood Business for Ocean Stewardship

4TH WORKING MEETING – UPDATES AND OPPORTUNITIES

Time (GMT+2)	Monday, May 17
**08:15 – 08:45**	**OPENING REMARKS AND OVERVIEW**
08:15 – 08:25	Opening statement ([redacted])
08:25 – 08:40	Welcome and presentation "Where have we come from over the past five years" ([redacted])
08:40 – 08:45	Overview of meeting and anti-trust reminder ([redacted])
**08:45 – 12:30**	**PROGRESS TOWARDS OUR GOALS AND COMMITMENTS**
08:45 – 11:20	**TASK FORCE I (IUU FISHING; ENDANGERED SPECIES; AND MODERN SLAVERY)**
	Update on progress using toolkit and voluntary actions; company progress reports
08:45 – 08:55	Summary update of Task Force I work ([redacted])
08:55 – 09:05	Summary of progress reports from companies ([redacted])
09:05 – 09:15	Pathways to operationalizing risk maps ([redacted])
09:15 – 09:35	Breakout groups to discuss opportunities and progress (All)
09:35 – 09:45	Reporting back; summation ([redacted])
09:45 – 10:00	Break
	Endangered species recommendations for time bound goals to minimise impacts
10:00 – 10:10	Science presentation ([redacted])
10:10 – 10:20	Presentation on key elements of an endangered species strategy ([redacted])
10:20 – 10:40	Breakout groups to consider key elements, feasibility, ambition level (All)
10:40 – 10:50	Summary of responses ([redacted] / All)
	Reducing IUU fishing; eradicating modern slavery, and minimising impacts on endangered species throughout our supply chains
10:50 – 11:05	Meta-coalition discussions ([redacted] All)<br>Summary update and goals of meta-coalition, and opportunity to use that to achieve transformations using science-based solutions from Task Force I
11:05 – 11:25	Presentation on opportunities to link via supply chain support for traceability and electronic monitoring programs ([redacted])
11:25 – 11:30	Short Break

11:30 – 12:30	**TASK FORCE VI (CLIMATE RESILIENCE)**
11:30 – 11:40	Summary update of Task Force VI work and progress reports ([redacted])
11:40 – 11:55	Presentation on Race to Zero Campaign ([redacted])
	Presentation on Science Based Targets Initiative ([redacted])
11:55 – 12:05	Discussions on presentations ([redacted] All)
12:05 – 12:20	Presentation on climate impacts on SeaBOS member identified fisheries ([redacted])
12:20 – 12:30	Discussions on EDF presentation and future work priorities ([redacted])

Tuesday, May 18

**08:15 – 12:30**	**PROGRESS TOWARDS MEETING OUR GOALS AND COMMITMENTS, CONTINUED**
08:15 – 08:20	Reminder on anti-trust and meeting organisation ([redacted])
08:20 – 09:40	**TASK FORCE III (WORKING WITH GOVERNMENTS AND AMR)**
08:20 – 08:30	Summary update of Task Force III work and progress reports ([redacted])
08:30 – 08:45	Presentation on antibiotics survey results ([redacted])
08:45 – 08:55	Discussion on results, and proposed approach ([redacted] All)
08:55 – 09:25	Breakout groups to discuss approach for Code of Conduct development, and Roadmap for ways to significantly reduce and/or phase out from aquaculture operations the use of High Priority Critically Important antimicrobials for human health, and Critically Important antimicrobials for human health (All)
09:25 – 09:40	Summary of responses and discussion (All / [redacted])
09:40 – 09:50	Update on UN Food Systems Summit and COP26 ([redacted])
09:50 – 10:05	Break
10:05 – 10:45	**TASK FORCE V (PLASTICS)**
10:05 – 10:15	Summary update of Task Force V work and progress reports ([redacted])
10:15 – 10:35	Breakout groups to discuss how best to unlock the partnership with Global Ghost Gear Initiative and International Coastal Cleanup, as well as considering plastic packaging footprint evaluation, towards transformation for a healthier ocean
10:35 – 10:45	Summary of responses and discussion (All / [redacted])
10:45 – 11:45	**TASK FORCE II (COMMUNICATIONS)**
10:45 – 11:00	Summary update of Task Force II work ([redacted])
11:00 – 11:15	Presentation of draft Communications Strategy ([redacted])
11:15 – 11:40	Breakout groups to discuss communications strategy and approach
11:40 – 11:50	Summary of responses and discussion (All / [redacted])
11:50 – 11:55	Short break

11:55 – 12:15	**TASK FORCE IV (TRANSPARENCY AND GOVERNANCE)**
11:55 – 12:05	Update of Task Force IV work ([redacted])
12:05 – 12:15	Discussions on future structure, function, and funding of SeaBOS (All)
12:15 – 12:30	**Progress and transformation: Are we on track for October? ([redacted])**

Wednesday, May 19

**08:15 – 12:30**	**MAINTAINING VISION AND STEWARDSHIP**
08:15 – 08:20	Meeting outline and anti-trust ([redacted])
08:20 – 08:30	Transformation for ocean stewardship ([redacted])
08:30 – 09:35	**Segment one: Food and Finance**
08:30 – 08:50	*Blue Food*
	Update presentations on collaborations<br>• Blue Foods Assessment and Blue Foods Coalition ([redacted])<br>• UN Food Systems Summit, COP26, and associated events ([redacted])
08:50 – 09:50	*Blue Finance*
08:50 – 09:05	Stage-setting presentation ([redacted])
09:05 – 09:10	Blue Bonds presentation ([redacted])
09:10 – 09:15	Sustainability loans presentation ([redacted])
09:05 – 09:10	UNGC Finance Action Platforms presentation ([redacted])
09:10 – 09:30	Breakout groups on how blue finance and blue food can be operationalized in the stewardship agenda and what that could mean for SeaBOS (All)
09:30 – 09:40	Summary of responses (All / [redacted])
09:40 – 09:50	Break
09:50 – 10:50	**Segment two: The opportunities of "30 x 30"**
09:50 – 10:05	Presentation on background to "30 x 30" ([redacted])
10:05 – 10:15	Presentation of SFP approach to marine protected areas (Sustainable Fisheries Partnership)
10:15 – 10:25	Presentation ARK spatial closures on krill ([redacted])
10:25 – 10:45	Breakout groups to discuss how spatial management and protected areas can contribute to ocean stewardship and ocean health, and what they mean for sustainable seafood production (All)
10:45 – 10:55	Summary of responses (All / [redacted])
10:55 – 11:10	Break

11:10 – 12:10	**Segment three: Ocean Equity**
11:10 – 11:30	Presentation on Ocean Panel Blue paper ([redacted])
11:30 – 11:50	Breakout groups to consider how equitable access to ocean resources and benefits can be fairly distributed, and that the most vulnerable are protected from harm, in the context of providing for sustainable seafood production (All)
11:50 – 12:00	Summary of responses (All / [redacted])
12:00 – 12:30	**Final wrap-up and close of meeting** (All)

List of background documents

Scientific background paper

1. *Endangered Species and Loss of Marine Biodiversity*

Scientific scoping papers

1. *Ocean Finance*
2. *Marine Protected Areas and 30x30*
3. *Ocean Equity*

Reference paper

1. *Summary outcomes on time-bound goals*

THE
KEYSTONE
DIALOGUE
October 2021 Virtual Meeting

PROGRAM
October 2021

Seafood Business for Ocean Stewardship

DELIVERING TRANSFORMATION FOR OCEAN STEWARDSHIP

Time (GMT+2)

Monday, Oct 4th

08:00 – 08:10 **Administration**

Meeting arrangements ([redacted])
Reminder on anti-trust legislation and requirements ([redacted])

08:10 – 09:40 **SESSION 1: Where do we want to be?**

08:10 – 08:15 **Formal welcome and opening of meeting** ([redacted])

08:15 – 08:30 **Five years after the first Keystone Dialogue - is SeaBOS a success?** ([redacted])

08:30 – 09:00 **The future – brief company perspectives** *on the most important steps taken this past year on SeaBOS commitments, and challenges and opportunities experienced in the journey to support SeaBOS goals, including to have no IUU fishing or modern slavery in our own operations, and setting climate targets.* (Four minutes for each CEO)

- [redacted]
- [redacted]
- [redacted]
- [redacted]
- [redacted]
- [redacted]
- [redacted]
- [redacted]

09:00 – 09:20 **The journey from a company perspective - how are we moving to transformations?** ([redacted])
To outline and explain what their journey with SeaBOS has been like so far; to identify the challenges they have had to overcome and believe are likely to face; and to express where they are striving to get to with SeaBOS in the future.

09:20 – 09:40 **Reflections and discussions** ([redacted] to lead joint discussions)

09:40 – 09:50 *Short break (please do not leave meeting, just mute audio and stop video)*

09:50 – 11:20 **SESSION 2: Where are we at - do we have a solid foundation?**

09:50 – 09:55 **Time bound goals from October 2020** ([redacted])
Overview of this session, to be focussed about reporting on goals from October 2020, and discussing activities and outcomes to date.

09:55 – 10:05 **What is credible reporting on time bound goals (and how far are we from it?)** ([redacted])

10:05 – 10:40 **Task Force I (IUU fishing/endangered species/forced labour): Results and outcomes**

- Presentation from [redacted] Activities, results, outcomes (10 minutes)
- Presentation from [redacted] on Nissui supply chain traceability (15 minutes)
- Presentation from [redacted] on outcomes (10 minutes)

10:40 – 11:25 **Task Force III (Working with governments and antibiotics): Results and outcomes**

- Presentation from [redacted] Fisheries Agency of Japan on new fisheries legislation (15 minutes)
- Presentation from [redacted] on Blue Food Assessment results and outcomes (10 minutes)
- Presentations from [redacted] Activities, results, outcomes (20 minutes)

11:25 – 11:30 *Short break (please do not leave meeting, just mute audio and stop video)*

11:30 – 12:30 SESSION 2, CONTINUED

11:30 – 11:45 **Task Force V (Plastics): Results and outcomes**

- Presentation from Dongwon on efforts in plastics reduction (10 minutes)
- Presentation from [redacted] (recorded): Activities, results, outcomes (5 minutes)

11:45 – 12:15 **Task Force VI (Climate resilience): Results and outcomes**

- Presentation from [redacted] on climate resilience (10 minutes)
- Presentation from [redacted] Activities, results, outcomes (20 minutes)

12:15 – 12:30 **Reporting on SeaBOS progress** (facilitated by [redacted])

Summary discussions on the communique from this meeting which touches on all elements of the October 2020 goals, and resultant outcomes. Also, to pose questions and a quick summary to think about overnight. Are we doing enough? Are we spread too thinly and need to focus even more? Where is evidence of transformation and what can we do to enhance that?

Tuesday, Oct 5th

08:00 – 08:10 Administration

Reminder on anti-trust legislation and requirements ([redacted])
Review of Day One ([redacted])

08:10 – 12:30 SESSION 3: WHERE ARE WE GOING NEXT?

08:10 – 09:10 **Future of Task Force I (IUU fishing/endangered species/forced labour)** ([redacted])

- Presentation from [redacted] on eliminating IUU fishing, followed by discussions (20 minutes)
- Presentation from [redacted] on eliminating forced, bonded and child labour practices, followed by discussions (20 minutes)

- Presentation from [redacted] on endangered species, followed by discussions (20 minutes)

09:10 – 10:10 **Future of Task Force III (Working with governments)** ([redacted])

- Anti-microbial strategy and direction [redacted] (15 minutes)
- How to take next steps together to address AMR ([redacted] 10 minutes)
- Working with governments and SeaBOS in the context of changing ocean governance ([redacted] 15 minutes)
- How to advance working with governments ([redacted] 10 minutes)
- Discussions to be led by [redacted] (10 minutes)

10:10 – 10:20 *Short break (please do not leave meeting, just mute audio and stop video)*

10:20 – 10:45 **Future of Task Force V (Plastics)** [redacted])

- Future direction and strategy (Myoung Woo Lee 10 minutes)
- Outline strategy and discussions (to be led by [redacted] 15 minutes)

10:45 – 11:15 **Task Force VI (Climate Resilience)** ([redacted])

- Next steps and strategic direction on climate action and resilience ([redacted] 15 minutes)
- Discussions led by [redacted] (15 minutes)

11:15 – 11:30 **Task Force IV** ([redacted])

- Structure, budget, legal, administration (5 minutes)
- Discussions led by [redacted] (10 minutes)

11:30 – 11:40 *Short break (please do not leave meeting, just mute audio and stop video)*

11:40 – 12:10 **Task Force II (Communications)** ([redacted])

- Presentation from [redacted] on strategy (10 minutes)
- Presentation from [redacted] (10 minutes)
- Discussions led by [redacted] on the Communications strategy and process to tie it all together (10 minutes)

12:10 – 12:20 **Discussions** (facilitated by [redacted])

12:20 – 12:30 **Closing comments** ([redacted])

12.30 **Formal close of meeting** ([redacted])

List of background documents

Information sheets

- *Elasmobranchs*
- *Seabirds*

Reference documents

- *Draft Endangered Species Strategy*
- *Blue Food Assessment*
- *Draft Antibiotics Roadmap*
- *Draft Communications Strategy*

Agenda papers

- *Task Force I (IUU fishing, endangered species, forced labour) summary update*
- *Task Force II (Communications) summary update*
- *Task Force III (Working with governments and AMR) summary update*
- *Task Force III 'Strategy Process' background paper*
- *Task Force IV (Transparency and governance of SeaBOS) summary update*
- *Task Force IV 'Finance and structure' paper*
- *Task Force IV 'Administration and policy' paper*
- *Task Force V (Plastics) summary update*
- *Task Force VI (Climate Resilience) summary update*
- *Science team summary update*
- *Final outcomes of SeaBOS Virtual Working Meeting, May 2021*
- *Draft communique from 2021 Keystone Dialogue*

SeaBOS

SeaBOS Endangered Species Strategy

Time-bound goals

1. Put science-based and operational measures in place that, when combined, substantially reduce the risk of harm to endangered elasmobranch (sharks & rays) and seabird species from our own operations by October 2022; and substantially reduce the risk of harm to these species in operations which are part of our supply chains.
2. We will publish, by January 2022, a list of "best practice" measures for limiting harm to endangered elasmobranch and seabird species.
3. By May 2022 we will develop a monitoring and reporting framework for interactions with endangered elasmobranch and seabird species for adoption in October 2022.
4. We will report in October 2023 and October 2025 on progress towards meeting Goal 1 and the rapid action intended to mitigate harm to endangered species whenever identified.
5. Drawing on lessons learned from focusing on elasmobranchs and seabirds in this initial phase of endangered species work, initiate process in October 2023 to expand the scope and focus of the SeaBOS endangered species work, with an aim to eliminate, in a step wise fashion, all negative impacts on endangered species.

Purpose: SeaBOS members are committed to advancing ocean stewardship, including by engaging in sustainable seafood production. Sustainable production can only happen if impacts on endangered species are minimized.

Definition: For the purposes of SeaBOS, we define "Endangered species" as the list of species identified as Vulnerable, Endangered or Critically Endangered by the IUCN, refined as appropriate with more recent or detailed scientific assessments, as well as those species designated as endangered, threatened or protected by relevant governmental or intergovernmental bodies.

Approach: SeaBOS members have committed to increase transparency about their own operations, evaluate if and how endangered species interact with their own operations, determine how negative interactions can be minimized and implement appropriate actions. This strategy aims to improve knowledge and advance transparency, while also ensuring that existing (and emerging) practices aimed at reducing risks to endangered species and their habitats are more widely applied across all aspects of the seafood industry, including fisheries, feeds and aquaculture operations. SeaBOS members working together with scientists will develop and pilot science-based solutions including evaluation of novel technologies that can help monitor the status of endangered species, mitigate negative impacts or incentivize compliance. Leadership will be provided on stewardship initiatives for species and habitats that are experiencing declines in health and coverage. This strategy is based on experiences derived from previous work in Task Force I, and similarly includes a series of iterative and co-designed steps.

Scope: Given the large number of species that are defined as endangered, we will initially focus (2021-2023) on elasmobranchs and seabirds as starting points for action.

Future scope: Recognizing the complexity and diversity of actions and learning required to achieve sustainable seafood production, at last three elements will be of particular focus when advancing the strategy beyond its initial scope in 2023. This expansion involves extending the activities to additional taxonomic groups (e.g., other fish species, mammals, reptiles), engaging in further actions aimed to prevent and reduce overfishing, including through building synergies with other SeaBOS Task Forces, and attention to the conservation and restoration of habitats and ecosystems of crucial importance for endangered species. Individual efforts in these areas are already underway, and will be systematically collected by the science team during the next few years, in order to support future expansion and a comprehensive approach to stewardship.

Why SeaBOS?

The international community has already developed clear international plans of action and management measures on seabirds and elasmobranchs and there are opportunities for SeaBOS companies to support such existing measures. SeaBOS has a unique capacity to accelerate existing means to reduce threats and an initial focus on these two species groups in a first phase (2021-2023) will generate new knowledge and practice, can contribute to mainstreaming existing approaches, and contributes to ocean stewardship. Our initial work with seabirds and elasmobranchs will provide an opportunity to collaboratively learn about best practices with an aim to expand such knowledge and practice also to other species groups.

A stepwise approach to achieve these time-bound goals

1. Scientific assessment and reporting

A range of scientific data sources include information on endangered species, their status, geographical distribution and interaction with seafood production. We will draw on the leading scientific organisations and ensure that information is collected and synthesized in ways that inform the strategic decisions and priorities of SeaBOS members.

2. Internal due diligence and assessment

A questionnaire will be developed for each SeaBOS company to help develop an understanding of potential and existing interactions between endangered species and seafood operations, whether or not the species is retained or discarded, as well as interactions with relevant habitats and ecosystems. This may involve consideration of target species & non-target species interactions, gear or infrastructure involved, or the geographic areas and time of the year at an appropriate resolution. Such identification would start with own operations and progressively also involve activities of supply chains.

3. Inventory of best practices and relevant organizations

Multiple existing policies (internal company policies, codes of conduct related to suppliers) and practices are available for reducing negative impacts on endangered species (e.g. through avoidance, mitigation and reduction of post-release mortality rates). Examples of existing best practice and novel technologies to minimize impacts on endangered species will be compiled and communicated to SeaBOS members. External expert organizations will be identified for potential partnerships.

4. Company revision to codes of conduct, procurement and other policies

Shared learning about problems, best practices and company specific priority areas will ensure that companies are able to engage in relevant activities. Actions may inform revisions of codes of conduct or updating of procurement policies.

5. Co-producing company specific solutions

Not all actions are necessary everywhere and all the time. We will for instance, focus on identifying company-specific areas of risk and opportunities for mitigating impact on endangered species, supported by strengthened monitoring, transparency and traceability, while also recognizing that sustainable management efforts that are transparent are more likely to be viewed as credible by stakeholders.

6. Definition of voluntary actions and associated reporting

This process includes the demonstrable removal of risks to endangered species from supply chains and implementation of existing policies to reduce impacts on endangered species, primarily based on existing best practices in wild capture and aquaculture operations, national legislation and guidelines, conservation organizations, and Regional Fishery Management Organization (RFMO) conservation and management measures (CMMs). This will support translating scientific knowledge and practice that are 'fit for purpose' in fisheries and aquaculture production. An increase in transparency via reporting can help to illustrate how each company is exposed to risks associated with harming endangered species.

7. Innovation in policy, scientific knowledge and practice

SeaBOS members have an ability to influence the existing practice associated with endangered species, but can also play an important part in generating new scientific knowledge, leading restorative stewardship and advocating for better policies – this applies to their individual national context, international organisations in which they are active (e.g. RFMO), certification bodies, and elsewhere.

This strategy was developed by SeaBOS Task Force I, with scientific support from the **Stockholm Resilience Centre** at Stockholm University, the **Beijer Institute of Ecological Economics** and the **Global Economic Dynamics and the Biosphere program** at the Royal Swedish Academy of Sciences, **University of Lancaster** and the **Stanford Center for Ocean Solutions** and finacial support from the **Walton Family Foundation**, the **David and Lucile Packard Foundation**, and the **Gordon and Betty Moore Foundation**.

SeaBOS

Antibiotics Stewardship Roadmap

Agreements on approach

- **Agree** to the stepwise process of the road map proposed in this paper that will form a "SeaBOS Antibiotics Code of Conduct" by October 2022
- **Agree** that the scope of the SeaBOS Antibiotics Code of Conduct includes members' own operations and to extend that into their supply chains engaged in aquaculture and feed production
- **Agree** to cease the use of HPCIA and CIA* in all areas of aquaculture production where their use is not specifically enabled through national legislation**
- **Agree** to a virtual workshop in February 2022 to refine the actions required and the metrics to demonstrate progress
- **Agree** to engage with annual SRC surveys on antibiotics stewardship in our own aquaculture operations, including from supply chains engaged in aquaculture production, to monitor and measure progress

* HPCIA and CIA = High Priority Critically Important Antimicrobial and Critically Important Antimicrobial for human health, as defined by the World Health Organisation (WHO).

** Where region-specific national legislation does exist providing for the use of HPCIAs or CIAs, members will work collaboratively with a range of appropriate experts, such as pharmaceutical companies, veterinarians, intergovernmental agencies, or governmental departments, to determine or develop effective alternatives such as vaccines or lower category antimicrobials, to ensure the health and wellbeing of those seafood products and reduce the use of HPCIAs and CIAs. We will report back in October 2023 on progress towards this goal.

Executive Summary

The threat of anti-microbial resistance (AMR) to human, animal and environmental health is high and growing. Anti-microbial residues are found in some farmed products and in the local environment around some aquaculture operations. Globally, inappropriate use of antibiotics in human medicine and animal health increases the risk of organisms becoming resistant to the key medications that we have. Whilst antibiotics are important tools to protect the health of humans and animals, it is essential that good stewardship is applied to their use, so that the risk of AMR is reduced, and the most critically important antibiotics are used in the most appropriate ways. The "One Health" concept, which shows that environmental health, human health, and animal health are all inter-related, should be applied, and through improved management of animal health, we can improve human and environmental health as well. This sets up the challenge to SeaBOS.

Aquaculture is flagged as a major user of antibiotics and whilst sectors of the industry are working towards reducing their use, the general lack of data on antibiotic use in some parts of the industry hampers efforts to demonstrate progress or refute broad allegations. However, there is also a real concern on health management in aquaculture, where the species, farming conditions and the diseases are so diverse that appropriate treatments are not always available.

A survey of SeaBOS members showed the need for tracking antibiotic use in aquaculture operations. There are differences in use between species and geographies. Working with suppliers increases the complexity.

The sensitivity of this work has highlighted the need for trust when conducting such surveys, and also the need to recognise and reward transparency where best practices may not yet be in place. Only by building such trust, can we identify where and how improvements can be made. Future surveys will be required to track progress from the baseline.

Discussions with various experts and organisations showed the urgency to address this issue, as an increasing number of studies have raised public, industry and government awareness. The discussions also highlighted the complexity of finding a common approach across diverse aquaculture operations and guided us to the concept of a high-level approach to the issue for SeaBOS. This led to a proposal for a SeaBOS Antibiotics Code of Conduct which can enable our members and other operations identify the pathway to improved antibiotic stewardship to reduce the risk of AMR. We had hoped to propose a roadmap and timeframe for improvement by October 2021, but the complexity of the issue has delayed this, and we hope to be able to address this further in a dedicated virtual workshop in February 2022.

The proposed SeaBOS Antibiotics Code of Conduct commits members to the Principles of Aquaculture Health Stewardship. These show a progression (see figure) from obtaining and managing data on stock health and health management, through improved antibiotic stewardship and developing a diagnostics service and obtaining access to appropriate treatments. Going beyond this, building capacities for a veterinary prescription service and detailed health plans on a site-by-site basis which leads up to appropriate area management strategies in collaboration with other local and regional aquaculture operations. Together this will lift aquaculture production up to a higher state of biosecurity management. The details of each level and measurable outcomes to verify the level has been achieved will be confirmed in the February 2022 workshop, together with details of how the Extended Producer Responsibility (EPR) of suppliers and subsidiaries will be addressed.

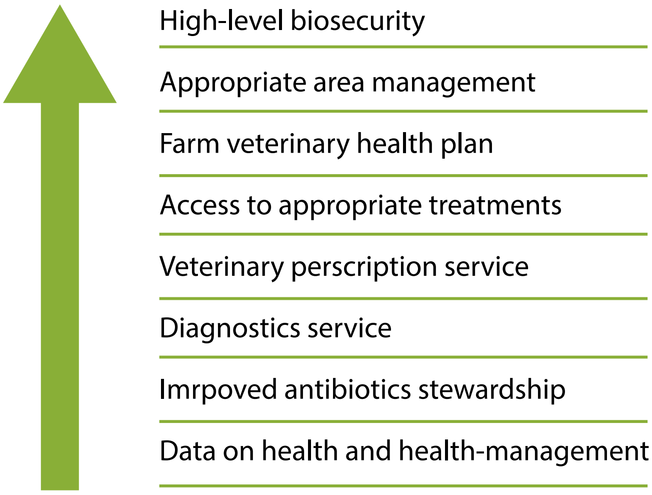

A critical part of SeaBOS membership is to demonstrate change towards our goals. Through regular surveys, members will be able to show progression against the proposed Principles over time. We will incorporate an annual on-line (confidential) survey, using a simpler format once the baseline data are established. We are proposing a three-tier system recognising basic, medium and high level progress on biosecurity, which will be substantiated by the survey data.

Workstream Activities and Proposals for 2022

Data

Since October 2020, we worked with companies individually to complete a survey of antimicrobial use based on each company and their subsidiaries. The survey on antimicrobial use in aquaculture highlighted many areas where we could make improvements, but more resolution is needed to identify the right interventions. We thus aim to reach out to companies during the remainder of this year for complementary data. This will provide an excellent opportunity to draw up trends in antibiotics use and verify the success of future interventions. We will also identify opportunities for members to share knowledge on a pre-competitive basis, to help each other to progress.

Whilst data were provided by all, the SRC team need to work with some of the companies to get increasingly detailed, quality data. It was also clear that it is essential to extend data collection to both subsidiaries and suppliers, to achieve a clear understanding of the challenges and possible solutions.

Access to the supply chain data is critical if we are to show progression and SeaBOS leadership in this area, and also in order to best guide the remainder of the work that needs to be carried out by the working group. We understand the contextual importance of how data is presented to accurately reflect the situation in production. However, in order to better demonstrate the context, we need higher resolution data of good quality. If we do not have good quality data, our work will not appropriately reflect the overall situation in SeaBOS, nor allow us to benchmark improvements and share learnings with others towards transformation.

Whilst we recognise that companies carry out export controls on aquaculture products, demonstrating that these products have antibiotic residues within legal limits, it is critical to have data also covering the on-farm use. Over-use, inappropriate use (such as using the wrong active ingredient or using antibiotics on diseases which do not respond to antibiotics), and not completing the appropriate dose are all examples of poor antibiotic stewardship. If stewardship is currently good, we should be able to report this – if it not good, we can work together to find solutions to improve it.

During early 2022 SRC will have completed the survey of

existing legislation and third party certification schemes in the countries where members have aquaculture operations, or aquaculture producing supply chains. This covers at least 24 countries and multiple languages, but it will be an important step to understanding the context of how the SeaBOS Antibiotics Code of Conduct will have to be applied. It will also enable us to identify legislative opportunities and potential barriers to good antibiotic stewardship and aquaculture health management. These innovations can then be addressed with the relevant governments as part of the work of Task Force III.

Collaborations

We have been engaging with a broad network of experts on antimicrobials in aquaculture as well as international organisations working towards improved understanding about the problem and potential solutions through multi-stakeholder dialogues. We have identified key experts in Australia and Hong Kong who can help identify the best ways to use antibiotics if and when they are required, but who are also looking at alternative options, particularly vaccines for fish.

We have had similar discussions with ASC (Aquaculture Stewardship Council) and BAP (Best Aquaculture Practice) initiatives who both promote good antibiotic stewardship through their certifications. Meetings have also been held with WorldFish, IDH, and Monterey Bay Aquarium who all have programs to reduce antibiotic use in aquaculture in different regions, with WorldFish and IDH particularly focussing on small scale farmers who are the least supported in terms of health support. From these discussions, we feel that we have generated a good network who SeaBOS can collaborate with, to support us develop towards our commitments.

SeaBOS Antibiotics Code of Conduct

We have reviewed a variety of options for the SeaBOS Antibiotics Code of Conduct, from very detailed and prescriptive to very high level. We have agreed that a high-level approach is best for SeaBOS, given the variety of interests and activities the members have. Our aim to create a framework which can be applied by all members, to their own activities and to subsidiaries and suppliers engaged in aquaculture production. This will provide a series of developmental steps required, not just for good antibiotic stewardship, but ultimately good animal health – which of course is good for the farmers and for sustainable aquaculture. This supports the "One Health" concept, whereby improved animal health will also support environmental and human health.

Our approach to this has been to develop a "ladder" of improved aquaculture health stewardship, building up core units as Principles. This is based on expectations from a broad range of existing third-party standards and expert opinions. Each step helps improve the overall aquaculture health stewardship, reducing the need for antibiotics and ensuring that where they are used, they should be used appropriately.

The ladder concept is summarised in the Executive Summary, but in Table 1, we show the concepts behind each step that must be put in place. A more detailed version will be developed with members to detail the type of actions required to achieve each Level. These can be documented and provided as evidence to support the progress up the ladder, which can be measured in direct operations and in suppliers as appropriate.

The details of the actions and supporting metrics ideally required to demonstrate the achievement of each level will be resolved with members and external experts in a workshop for this workstream. The plan is to hold a virtual workshop in February 2022, with an option to follow up with another workshop at the SeaBOS annual Working Meeting in Stockholm in May 2022.

As the ladder has many steps, we are proposing to simplify demonstration of progress into 3 levels, for example as suggested in Table 2. This will make communication on the degree of progress clearer. Using such a system, we can envisage engagement with key stakeholders such as retailers on this topic. Retailer support and pull for improvement will be essential for the long-term success of anti-microbial reductions. This approach will enable greater clarity for retailers and consumers on the use of antimicrobials in the seafood they purchase. A similar recognition is sought through Task Force I for progress on reducing IUU fishing and improving labour conditions.

Retailer engagement will provide a clear incentive for members and their supply chains to demonstrate progress against the SeaBOS Antibiotics Code of Conduct. But it is clear that other incentives for progress should be investigated through the SeaBOS work.

A key early step in the ladder will be to agree to exclude the use of HPCIAs and CIAs for human health as defined by the WHO, where they are not already provided for in national legislation. Where region-specific national legislative provisions do exist providing for the use of HPCIAs or CIAs, we will work collaboratively with a range of appropriate experts, such as pharmaceutical companies, veterinarians, intergovernmental agencies, or governmental departments, to determine effective alternatives such as vaccines or lower category antimicrobials, to ensure the health and wellbeing of those seafood products and reduce the use of HPCIAs and CIAs. This workstream will report back in October 2023 on progress towards this goal.

Through the SeaBOS Antibiotics Code of Conduct, SeaBOS members will support farm operations in their supply chains to apply standards at least equivalent to the SeaBOS Principles of Aquaculture Health Management and move through the ladder towards the top. In the first year, members will be provided with feedback from SRC on the current positions on the ladder for their own and supply chain production from the data provided. In subsequent years, the aim is to support and

**Table 1:** Description of the suggested status achieved at each level by a company in both their own operations and in their supply chains.	
**Levels**	**Description of preferable status**
Basic-level for health and health management	Complete data on stock health and health management for each farm site in place
Improve antibiotic stewardship	Antibiotic use is carefully managed and are used in accordance with recommendations and in responsible ways.
Diagnostics service	Capacity for rapid diagnoses of diseases exist on farm or are readily available within company, which enables recommendations for appropriate treatments.
Access to appropriate treatments	Correct treatments are ensured through knowledge and access to appropriate and good quality antibiotics.
Veterinary prescription service	A professional health care system is in place, ensuring that all medical treatments are prescribed by trained practitioners.
Farm veterinary health plan (VHP)	Annual health plan is in place for each farm, and this has been established in consultation with the associated veterinarian.
Appropriate area management	Health plan across neighbouring farms is in place and been established through collaboration between companies/farms.
High-level biosecurity	Area management considering farm connectivity is in place and this is being complemented by also considering risks from environmental quality changes.

**Table 2** Suggested three tier approach to recognizing progress			
**Achievement**	**Basic**	**Intermediate**	**High**
Data on health and health management	✓	✓	✓
Improved antibiotics stewardship	✓	✓	✓
Diagnostics service	✓	✓	✓
Veterinary prescription service	✓	✓	✓
Access to appropriate treatments	✓	✓	✓
Farm veterinary health plan (VHP)			✓
Appropriate area management			✓
Biosecurity Assessment	Basic	Intermediate	High

encourage members to move up the ladder steadily, importantly applying good antibiotic stewardship at an early point. We recommend establishing milestones in 2023 and 2025 for supply chains, to give members time to engage effectively with them.

Next Steps

With the agreement on the proposed Principles of Aquaculture Health Management and support for development of a SeaBOS Antibiotics Code of Conduct, the next step is to determine the starting position of each company and their supply chains. A road map can then be determined on how to progress through the next steps.

A virtual workshop will be convened in February 2022 with aquaculture health experts from the members and external experts on aquaculture health, focused on developing a systematic approach to its improvement. The outcomes from the survey data already provided by the members will be reviewed, maintaining confidentiality but enabling the current situation to be demonstrated. Options for progression will be discussed in more detail at this workshop, enabling members to explore these in more detail later. Before the workshop, further work with members will be carried out to improve on the existing data, to ensure the full scope of operations including supply chain production are covered, and that the data are suitably granular to show the members' performance against the various proposed criteria. SRC will also conduct a review of relevant national legislation on use of antimicrobials in aquaculture production, with support and input from companies.

This strategy was developed by SeaBOS Task Force III, with scientific support from the **Stockholm Resilience Centre** at Stockholm University, the **Beijer Institute of Ecological Economics** and the **Global Economic Dynamics and the Biosphere program** at the Royal Swedish Academy of Sciences, **University of Lancaster** and the **Stanford Center for Ocean Solutions** and finacial support from the **Walton Family Foundation**, the **David and Lucile Packard Foundation**, and the **Gordon and Betty Moore Foundation**.

Supplementary Data S2: Annual Reports to HRH Crown Princess Victoria of Sweden (2018-2020).

September 18, 2018

Your Royal Highness, Crown Princess Victoria of Sweden

It is our great pleasure, to present to You, on behalf of the Keystone Dialogues project and the Seafood Business for Ocean Stewardship (SeaBOS) initiative, the following update on our activities.

Please, let us first take this opportunity to thank You for Your inspiring and engaging leadership and passion, and for actively urging us on along our journey, in the Maldives (Nov 2016), in Stockholm (May 2017), New York (June 2017), and most recently in Karuizawa (Sept, 2018). We are indebted to the guidance, facilitation, and continuous support that You provide. Your role as Global Advocate for the SDGs, focusing on healthy oceans, provides additional incentives for SeaBOS members to seriously take on the challenge of ocean stewardship.

The Keystone actor hypothesis is generated by science and suggests that: *A small minority of powerful actors can influence a majority of smaller actors and cause cascading change. Shifting such keystone actors into ocean stewardship has the potential to become a short-cut towards sustainability*. Keystone actors are defined as companies that: *Dominate global production revenues and volumes, control globally relevant segments of production, connect ecosystems globally through subsidiaries, and influence global governance processes and institutions*. Ocean stewardship is defined as: *An adaptive and learning based, collaborative process, of responsibility and ethics, aimed to shepherd and safeguard the resilience and sustainability of ocean ecosystems for human well-being*.

Ten Keystone actors (transnational seafood corporations) are members of SeaBOS, including: *Maruha Nichiro Corporation, Nippon Suisan Kaisha, Thai Union Group, Marine Harvest ASA, Dongwon Industries, Nutreco (owner of Skretting), Cargill Aqua Nutrition, Cermaq (subsidiary of Mitsubishi Corporation), Kyokuyo,* and *Charoen Pokphand Foods*. The companies operate in wild capture fisheries and in aquaculture, and are based in North America, Europe and Asia. The agenda is to:

- Improve transparency and traceability in our own operations, and work together to share information and best practice, building on existing industry partnerships and collaborations.
- Engage in concerted efforts to help reduce IUU (Illegal, Unregulated and Unreported) fishing and seek to ensure that IUU products and endangered species are not present in our supply chains.
- Engage in science-based efforts to improve fisheries and aquaculture management and productivity, through collaboration with industry, regulators and civil society.
- Engage in concerted efforts to eliminate any form of modern slavery including forced, bonded and child labour in our supply chains.
- Work towards reducing the use of antibiotics in aquaculture.
- Reduce the use of plastics in seafood operations, and encourage global efforts to reduce plastic pollution.
- Reduce our own greenhouse gas emissions.
- Secure new growth in aquaculture, by deploying best practices in preventive health management, including improved regulatory regimes.
- Collaborate and invest in the development and deployment of emerging approaches and technologies for sustainable fisheries and aquaculture.
- Support novel initiatives and innovations for ocean stewardship.

As a group, SeaBOS companies have first focused their attention on ensuring that "the own house is in order". This is a long process which has only begun (Appendix 1). SeaBOS has been a source of inspiration and an important opportunity to learn from peers (see Appendix 2). Time has been invested to identifying priorities for action (supported by science) and ensuring that SeaBOS companies engage from a common starting position. This joint work between science and business,

is first focusing on ensuring there are no IUU products or modern slavery in SeaBOS member supply chains, and will increasingly also focus on reducing the use of antibiotics and plastics. We have engaged to substantially improve existing internal traceability systems and all member companies are working to become more transparent.

This work is conducted in five individual task forces, which have been coordinated and supported by the Stockholm Resilience Centre (SRC) at Stockholm University (in collaboration with the Beijer Institute and the GEDB programme of the Royal Swedish Academy of Sciences) in an "interim SeaBOS secretariat". SeaBOS members have recently agreed to fund and staff a more permanent solution, in a secretariat led by Knut Nesse, former CEO of Nutreco, one of the founding members of the initiative. Shigeru Ito, CEO of Maruha Nichiro Corporation, the world's largest seafood producer, is the first SeaBOS chair. SRC will remain the main scientific partner. This strong institutional foundation rests on Articles of Association, which all companies have committed to.

We find that our work is welcomed by industry peers, international organizations including the UN family, civil society organizations, academia, and governments (see Appendix 1). Its ripple effects extend far beyond our group. To take on the global ocean challenge is critical for wellbeing and survival, but also a task that we cannot do alone. To note that we have already inspired other industry leaders to take on a larger responsibility (e.g. within the UN Global Compact Ocean Action Platform) is therefore a very positive development. All SeaBOS members have received a letter of encouragement from a group of financial actors associated to the Principles for Responsible Investments (PRI).

As companies and academics, we realize that we have a lot to learn from each other. Together with You as a true source of inspiration, we have come to understand that change is indeed possible, and that we have a responsibility to do our best to enable change towards a healthy ocean. We will continue to work hard and lead by example – and we are looking forward to further opportunities to report to Your Royal Highness on our joint efforts, continued development and maturation as a global change maker and inspiration for seafood and other sectors.

Sincerely,

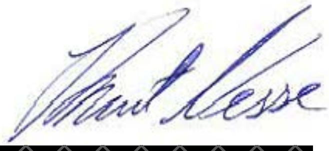

[redacted]

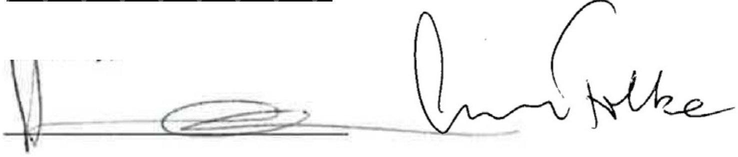

[redacted]

Appendix 1. SeaBOS progress to date

Joint SeaBOS action is currently taking place in five active task forces, led in collaboration between multiple member companies in collaboration with Stockholm Resilience Centre delivering the evidence-based scientific knowledge for action (see www.keystonedialogues.earth for more information).

- We have conducted a systematic survey of SeaBOS and a sample of their suppliers to develop a baseline understanding of how supply chains can be mobilized to achieve SeaBOS commitments
- We have engaged with UN agencies (FAO, ILO and WHO) to support their work on antibiotic resistance in aquaculture, social sustainability in wild capture fisheries and other ocean policy issues.
- We participated in the UN 2017 meeting on the ocean, hosted by Fiji and the Swedish government
- Funding has been secured for the secretariat and for SeaBOS as an organization.
- Mr Ito of Maruha Nichiro Corporation (the world's largest seafood producer) will serve as chairman of the new organisation and Mr. Knut Nesse has stepped down as CEO of Nutreco to serve as Managing Director of SeaBOS.
- SeaBOS members have agreed to develop a set of voluntary actions. These voluntary actions will build on a scientific understanding of the problems associated to IUU fishing, modern slavery and antibiotics, and an identification of where SeaBOS can have most impact.
- SeaBOS members have agreed to pilot block-chain technologies, with an ambition to discuss experiences and costs from such trials at the next SeaBOS meeting.
- SeaBOS members support the increased level of ambition among members, a move towards Global Reporting Initiative (GRI) reporting, and further learning opportunities.
- SeaBOS members have agreed to present an internal bench-marking of performance in transparency on an annual basis, supported by the SRC, with an ambition to develop a suggestion for joint and public reporting.
- SeaBOS members have agreed to produce a public statement that endorses the ongoing Global Dialogue on Seafood Traceability (GDST) aimed to develop global standards for seafood traceability
- SeaBOS members will provide leadership in reducing the use of antibiotics. The following five strategic priorities have been identified: a) improve awareness based on facts, b) improve internal surveillance and monitoring of antibiotics use, c) improved governance by regulatory agencies, d) sharing of best practices between companies and with international organizations, and e) investments to reduce antimicrobial use. The World Health Organization (WHO), Food and Agriculture Organization (FAO) and the World Organization for Animal Health (OiE) are identified as key partners.
- SeaBOS members will provide leadership to clean the ocean for plastics, have agreed that plastics is a key priority and will develop a strategy for SeaBOS members to address the plastics challenge, both within companies, with science, and with policy makers. A strategy will be presented and discussed at the next meeting.

Appendix 2. Member companies progress to date (by September 2018)

Maruha Nichiro Corporation
In 2018, Maruha Nichiro Corporation, the largest seafood company in the world, published its first integrated sustainability report, with a medium and long term sustainability strategy. The work has been ongoing since 2017 and the company has established a special team in 2018 to make sure sustainability is integrated in corporate activities. This work has strived towards a more sustainable corporate group as well as to contribute to achieving sustainable development goals through business activities. Maruha Nichiro Group will focus on three main values in business activities, *Economic Values, Social Values and Environmental Values*.

Nippon Suisan Kaisha (Nissui)
Nippon Suisan Kaisha has worked since 2016 with its revised sustainability strategy, which was published in February of 2018 in Japanese and English. The new strategy includes a new code of conduct and has drawn substantial knowledge and experience from the SeaBOS initiative. Among other things, the company has conducted a complete assessment of all its procured raw material and the corresponding status of the stocks from which they source. The company published its first materiality assessment in 2017 and has started reporting to the Carbon Disclosure Program (CDP) in 2016. The company is a member of the Global Sustainable Seafood Initiative (GSSI) and the Global Dialogue on Seafood Traceability (GDST).

Thai Union
Thai Union has been reporting in line with the Global Reporting Initiative (GRI) guidelines since the past five years through its publicly available annual sustainability reports. Thai Union regularly conducts a materiality analysis and has introduced "Trace My Report" in the company's 2017 Sustainability Report to improve traceability in their supply chain. Through their internal program *SeaChange*, Thai Union is advocating the SDGs, and it focuses on four main programmes including safe and legal labour. In 2017, the company signed an agreement with Greenpeace to drive positive change across the global seafood industry. In 2017, through their Business Ethics and Labour Code of Conduct, Thai Union contracted first tier suppliers, achieving 100 percent acceptance globally. In 2018, they aim to focus on the Vessel Code of Conduct for their wild- caught fish suppliers, focusing first on the tuna industry. In 2017, the company reduced GHG emission intensity (emissions per unit produced) by 11% compared to 2016, thereby saving USD 16 million.

Marine Harvest
Marine Harvest is the largest producer of farmed salmon in the world. They are reporting on sustainability in line with the SDGs, and *traceability, transparency and commitment to sustainability, quality, and food safety* are some of the main pillars of the company. In 2017, they implemented several energy-saving initiatives with the aim of reducing energy consumption in their plants to decrease GHG emissions by 10% by the end of 2018. The company is a member of Global Salmon Initiative (GSI) and certify their farms according to the standard developed by the Aquaculture Stewardship Council (ASC) a WWF endorsed certification. Marine Harvest is investing in research and development under their four main guiding principles: *Planet, Product, People and Profit* and are establishing a *Blue Revolution Centre* (BRC): a new, large-scale research station, which will be operationalized in collaboration together with the Norwegian University of Life Sciences (NMBU) and SINTEF Ocean. Marine Harvest has started to investigate the use of plastic material in their supply chain, in order to develop methods and approaches for all SeaBOS members to reduce the use of plastic in seafood production.

Dongwon
Dongwon has been focusing on improving its transparency through various efforts within SeaBOS, such as participating in a pilot study within SeaBOS to investigate the potential for using block-chain technology, in order to dramatically improve traceability in global supply chains. Dongwon is also engaging in SeaBOS taskforce to eradicate IUU fishing and modern slavery. Moreover, to proactively prepare for Protein Challenge 2050 and to connect fisheries and aquaculture, Dongwon is actively seeking collaboration with the aquaculture sector.

Skretting (subsidiary of Nutreco)

Skretting has a long tradition of transparent sustainability reporting and a long-term strategy aligned with the UN SDGs, consisting of four pillars: *nutritional solutions, ingredients, operations* and *commitment*. Nutreco is reporting based on the GRI Sustainability Standards and eight of the 17 UN-SDGs. In 2016, Skretting, Cargill Aqua Nutrition, and the National Fishing Association of Peru (Sociedad Nacional Pesquería) partnered to develop a comprehensive and ecosystem-based Fisheries Improvement Project (FIP) for the Peruvian anchovy fisheries. Nuterra, is Nutreco's sustainability program encompassing Skretting and other group companies. Nuterra is focuses on *community development, antimicrobial resistance (AMR),* and *innovative business development opportunities*. Nutreco has made substantial investment in community development since 2015, when the company launched assisting small-scale farmer's living at the poverty line in developing economies; to raise themselves out of poverty -consistent with the "creating shared value" concept. Some of these projects have resulted in doubling family income by guiding farmers on better production skills. Nutreco also conducts its annual *Global Community Day*, which in 2017, included 4000 people from 33 countries. Nutreco is performing a full materiality assessment in 2018 (for the 4th time) to ensure that perspectives from 600+ stakeholders polled from customers, suppliers, NGOs, internal management team and academia and resulted in a 42% response rate is being utilized to develop Nutreco's Roadmap to 2025.

Cargill Aqua Nutrition

Cargill Aqua Nutrition demonstrated its increased commitment to sustainability, primarily in relation to transparency and traceability, with the launch of its sustainability report in April 2018. The company employs a value chain approach to sustainability, starting with the needs of the consumer for healthy and sustainable fish, working back up the chain through farmers and their needs for healthy fish, to raw material suppliers and their environmental and social impacts. In previous reports, the company had provided sustainability reporting on their salmon feeds in Norway, Chile, Canada, UK, and Vietnam, but they now also report on their feeds produced for shrimp, tilapia and 30 other species, in all their 17 feed mills in 12 countries – a total of 1.5 million tonnes. Cargill Aqua Nutrition have managed to get all their 700 suppliers of raw materials to sign up to their Supplier Code of Conduct, which includes key aspects of environmental and social impacts, including risks associated to child labor and modern slavery. The company is active in the Seafood Task Force in Thailand in the Global Salmon Initiative (GSI) and work with an expressed commitment to the SDGs. Sustainable raw materials are the starting point of sustainable aquaculture and Cargill are delivering in this area. For marine ingredients, 29 of the fisheries used by Cargill hold MSC certification and 24 more are working to achieve this (reaching 45% of fishmeal and oil used). Further, of the marine ingredients used, one third was from trimmings and waste from fish caught for direct human consumption, and in total 30% of the raw materials used to manufacture feed were from by-products. Cargill is also addressing their waste, recycling 70% of the total weight generated.

Cermaq (subsidiary of Mitsubishi Corporation)

Cermaq has been rated the most transparent seafood company in the world in 2017 by *Seafood Intelligence*. *Transparency, performance*, and *partnership*, represent the three pillars of their sustainability strategy, which is also closely aligned with the SDGs. The salmon farming company is active in the Global Salmon Initiative (GSI), focusing on standardization and a sustainable development of the salmon industry, SeaBOS and the *FReSH* initiative, a joint project by EAT and World Business Council for Sustainable Development (WBCSD). Cermaq has played an instrumental part in developing the UN Global Compact Ocean Action Platform. Cermaq is engaging in Research and Innovation to strengthen its sustainability performance, including through exploring the concept *iFarm*, a sensor-based technology that will enable individualized farming, thereby improving environmental performance and increasing fish health and welfare.

Kyokuyo

Kyokuyo has published annual reports with social and environmental performance since 2007, and started publishing dedicated corporate sustainable reporting in 2017. The report for 2018 is in its final stages of preparation and will, for the first time, follow GRI reporting standards. A CSR division of Kyokuyo was established in January of 2018 in order to promote CSR activities, including by

integrating two different sections (one on Environment Managing Systems and another for research of world fisheries and international certificates), into one larger organization. A new chief of this division was appointed, to work with SeaBOS, as well as their Environmental Management System, and certification such as MSC/ASC and other standards.

CP Foods

Charoen Pokphand Foods (CPF) is listed as a member of the Dow Jones Sustainability Index for the 4th consecutive year in 2018 and a member of the UN Global Compact. The company is selected for the 2nd year, as a member of the FTSE4Good Emerging Indexes, which measures the environmental, social and governance performance of companies, in addition to receiving multiple other sustainability awards. The three pillars to sustainability in their strategy are: *balance of nature, self-sufficient society* and *food security*, and their reporting is aligned with the SDGs. The company is active in the *Seafood Task Force*, focusing on eliminating forced labor, human trafficking and IUU fishing, and is also a member of the *GDST* and the *FReSH* initiative.

SeaBOS

September 23, 2019

Your Royal Highness, Crown Princess Victoria of Sweden

It is our great pleasure, to present to You, on behalf of the Keystone Dialogues and the Seafood Business for Ocean Stewardship (SeaBOS) initiative, the following update on our activities.

Please, let us first take this opportunity to thank You for Your inspiring and engaging leadership and passion, and for actively urging us on along our journey, including in the Maldives (Nov 2016), Stockholm (May 2017), New York (June 2017), Karuizawa (Sept, 2018), and Bergen (May 2019). We are indebted to the guidance, facilitation, and continuous support that You provide.

Your role as Global Advocate Alumni for the SDGs, focusing on a healthy ocean, provides additional incentives for SeaBOS members to seriously take on the challenge of ocean stewardship. We very much appreciated having Your kind contributions forwarded to us at our meeting, and hope this outline may help update You on our progress.

Ten Keystone actors are members of SeaBOS, including: *Maruha Nichiro Corporation, Nippon Suisan Kaisha, Thai Union Group, Mowi, Dongwon Industries, Skretting, Cargill Aqua Nutrition, Cermaq (subsidiary of Mitsubishi Corporation), Kyokuyo*, and *Charoen Pokphand Foods*. The companies operate in wild capture fisheries and in aquaculture, and are based in North America, Europe and Asia.

Our commitments are to:

- Improve transparency and traceability in our own operations, and work together to share information and best practice, building on existing industry partnerships and collaborations.
- Engage in concerted efforts to help reduce IUU (Illegal, Unregulated and Unreported) fishing and seek to ensure that IUU products and endangered species are not present in our supply chains.
- Engage in science-based efforts to improve fisheries and aquaculture management and productivity, through collaboration with industry, regulators and civil society.
- Engage in concerted efforts to eliminate any form of modern slavery including forced, bonded and child labour in our supply chains.
- Work towards reducing the use of antibiotics in aquaculture.
- Reduce the use of plastics in seafood operations, and encourage global efforts to reduce plastic pollution.
- Reduce our own greenhouse gas emissions.
- Secure new growth in aquaculture, by deploying best practices in preventive health management, including improved regulatory regimes.
- Collaborate and invest in the development and deployment of emerging approaches and technologies for sustainable fisheries and aquaculture.
- Support novel initiatives and innovations for ocean stewardship.

As a group, SeaBOS companies are making progress, recognizing it is a long process, which has only recently begun (Appendix 1). Individual member companies have also made significant progress in the past year, in multiple areas, as outlined in Appendix 2. The individuals involved in this work are listed in Appendix 3.

SeaBOS formally established itself in June 2019 in Sweden, as the SeaBOS Fundraising Foundation, and appointed Martin Exel as Managing Director on 1 July, to progress the initiative as the head of the secretariat. SeaBOS members have paid their first tranche of membership fees to the Foundation, towards the purpose of providing for a healthy ocean, and ensuring sustainable aquaculture and wild catch fisheries.

The SeaBOS Fundraising Foundation has five Directors, including:

- [redacted]
- [redacted]
- [redacted]
- [redacted]
- [redacted]

We are very fortunate that [redacted] has agreed to participate on the Foundation, given her deep experience in the worlds of science, academia, and government; including in her past roles as the Under Secretary of Commerce for Oceans and Atmosphere, and the Administrator of the National Oceanic and Atmospheric Administration (NOAA). She was nominated by President Obama in December 2008 as part of his "Science Dream Team," and is a marine ecologist and environmental scientist by training, with expertise in oceans, climate change, and interactions between the environment and human well-being.

SeaBOS member companies agreed to establish a "SeaBOS Association" in coming months, to provide clarity of membership roles and criteria for new members, as well as ensure the proper governance of the stewardship from the companies can be maintained.

The work of SeaBOS over recent years has been conducted in five individual task forces, which have been coordinated and supported by the Stockholm Resilience Centre (SRC) at Stockholm University (in collaboration with the Beijer Institute and the GEDB program of the Royal Swedish Academy of Sciences). We have added a sixth task force on "Climate Resilience" aimed at addressing solutions for the challenges of climate change in the seafood industry, and to help identify the benefits of sustainable seafood production and a healthy ocean to the World, including reducing the impacts of climate change.

We are connecting with global initiatives to harness and enhance their reach, and to accelerate outcomes towards improvements across the spectrum of the Ocean and seafood sustainability. This year we intend to focus on issues such as reducing antibiotics use in seafood production; removal of ghost gear from our oceans (in conjunction with the Global Ghost Gear Initiative); new technology pilot programs for traceability in seafood supply chains (with the Global Dialogue on Seafood Traceability), and support for the UN Global Compact Initiative. We are also in active dialogue with the High-Level Panel for a Sustainable Ocean Economy.

At the core of our initiative is science, and the collaboration with industry leaders to achieve outcomes based on sound scientific data and analyses. With industry knowledge and expertise, we can create linkages across the multiple sectors and interest groups in ocean governance, and we will continue to do what we can to enhance progress, globally, to achieve the purpose of our Foundation.

As Your Royal Highness is well aware, the SeaBOS initiative, and the identification of its members, is based on a scientific analysis. Scientists from the Stockholm Resilience Centre, the Royal Swedish Academy, and the Beijer Institute (all based in Stockholm) continue to work closely with SeaBOS members, to ensure they have the science they need to be able to deliver on their commitments. An increased leadership by SeaBOS members of their initiative, now means that the responsibility and work load of scientists associated with coordinating and facilitating the work of SeaBOS, is reduced. This is now the task of the Managing Director (Martin Exel). The team of scientists will now instead increase their efforts in monitoring progress of the initiative, and draw out scientific insights from the process of facilitating the Keystone Dialogues, and its outcomes. Science will now continue to strengthen its role and ability to develop the frontier of science to the SeaBOS initiative and the Keystone Dialogues. The science foundation has proven to play a significant role for moving the SeaBOS process forward.

The scientific work to date has resulted in the establishment of strong collaborative partnerships between the Stockholm-based organizations, with the University of Birmingham, and with Stanford University, among others. Several additional scientific partnerships are in development. We are also very happy about having recently published a scientific report on the potential role of transnational corporations for sustainability, which has been directly inspired by this work, led by Prof. Carl Folke (https://www.nature.com/articles/s41559-019-0978-z).

The three philanthropy foundations (the Walton Family Foundation, the David and Lucile Packard Foundation, and the Gordon and Betty Moore Foundation), have funded the Keystone Dialogues from their origin, and the SeaBOS initiative to date. All three foundations continue to provide financial support for the science associated with this work. Adequate funding has been obtained until the end of 2021, thereby providing both longevity and flexibility.

We are looking forward to further opportunities to report to Your Royal Highness on our joint efforts; continued progress on transforming the global seafood sector, and source of inspiration for seafood and other sectors.

Sincerely,

[redacted]

[redacted]

[redacted]

[redacted]

SeaBOS

Stockholm Resilience Centre
Sustainability Science for Biosphere Stewardship

Stockholm University

THE BEIJER INSTITUTE OF ECOLOGICAL ECONOMICS

KUNGL. VETENSKAPS-AKADEMIEN
THE ROYAL SWEDISH ACADEMY OF SCIENCES

GLOBAL ECONOMIC DYNAMICS AND THE BIOSPHERE
THE ROYAL SWEDISH ACADEMY OF SCIENCES

Appendix 1. SeaBOS progress to date

Joint SeaBOS actions have taken place in five task forces, led in collaboration between multiple member companies, in conjunction with the Stockholm Resilience Centre and its partners, in delivering the evidence-based scientific knowledge for action (see www.seabos.org and www.keystonedialogues.earth for more information).

Highlights of SeaBOS progress from this year include:

- We have created a new Task Force on "Climate Resilience" in recognition of the substantial impact that climate change can have, and is having, on the seafood industry globally. We are also aware of the substantial health benefits and positive impacts at reducing global greenhouse gases by changing our diets from beef and other sources of land-based protein, to seafood. Sustainable aquaculture and wild catch production (with resultant increases in seafood production) could therefore benefit both human health and provide benefits for global climate mitigation.
- SeaBOS continues to support the Global Dialogue on Seafood Traceability (GDST) to connect experts, scientists, and industry. In addition, members agreed to scale up the successful proof of concept trial last year for block-chain technologies and traceability, as well as convene a workshop of invited experts in March 2020 to establish a clear outline of "key data elements" for any traceability platform, which could apply globally.
- SeaBOS members (Thai Union, Nutreco and Cermaq), together with SRC, hosted a panel at the North American Seafood Expo, in March 2019. A leading industry journal, covered the event, and wrote: "Anyone who attended the panel of the Seafood Business for Ocean Stewardship (SeaBOS) at this year´s Seafood Exhibition of North America (SENA) might just have heard the most important development in the entire seafood industry". https://www.undercurrentnews.com/2019/03/28/worlds-top-seafood-companies-see-seabos-shaking-up-entire-industry/
- SeaBOS members have participated in various scientific programs with SRC, including a survey on a series of possible "voluntary actions" which could be used as a future "resource kit" to address results from another piece of scientific work, on the IUU and forced labour Risk Mapping. That IUU risk map is expected to be finalised by May 2020, when the various options for how to address high risk ports by SeaBOS members will be refined, and pilot studies undertaken.
- SeaBOS Chairman Shigeru Ito, together with Norwegian Prime Minister Erna Solberg, published a letter in Financial Times, advocating for ratification of the UN Ports State Measures Agreement as an important tool to address illegal fishing, in June 2019. https://www.ft.com/content/fbe581e6-9819-11e9-8cfb-30c211dcd229
- SeaBOS members will continue to provide leadership in reducing the use of antibiotics in their operations and supply chains, and will host an expert workshop in May 2020 to devise key strategies for future action to reduce the use of antibiotics in aquaculture. There will also be a trial program to gather data and supply chain evidence to identify impacts of SeaBOS member operations, and review the implications of imposing a ban on the use of antibiotics which are for human health, in our aquaculture operations.
- Following a review of options on reducing plastics in our oceans, a webinar on a global initiative, and briefings from members, SeaBOS agreed to use a substantial portion of their membership fees to join the Global Ghost Gear Initiative, which aims to remove abandoned, discarded, and lost fishing gear from our Ocean. This partnership will be announced at "Our Ocean" conference in Oslo, on October 23 and 24 this year.
- Further on plastics, members have asked the task force to identify global initiatives which SeaBOS can support that are working to address the issues of micro-plastics, or packaging, or supply chain plastic use in the seafood industry. This will be progressed in the coming year.
- SeaBOS members agreed to 100% member companies reporting to the Global Reporting Initiative (GRI) standard by the next meeting of SeaBOS, in October 2020.
- SeaBOS members also agreed to collaborate with, and support the UN Global Compact Action Plan on Sustainable Ocean Initiative, including on programs such as transparency, traceability, and elimination of IUU.

- SeaBOS is part of the Advisory Panel to the High-Level Panel for Sustainable Ocean Economies, which is comprised of 14 Heads of Government and the UN Secretary General's Special Envoy for the Ocean. It is jointly Chaired by the Prime Minister of Norway and the President of the Republic of Palau, and is working on several initiatives where SeaBOS can have direct positive influence and impact. We are specifically looking to develop strategies and collaborations in the areas of climate resilience, and IUU elimination, over the coming year.
- Members have supported the Secretariat establishment having paid membership fees of USD50,000 per company for 2019, and continue to support the efforts of SeaBOS both financially and in their actions.
- [redacted] Maruha Nichiro Corporation (the world's largest seafood producer) has successfully Chaired the first meeting of SeaBOS since we established the Foundation; and
- [redacted] was appointed as the Managing Director of SeaBOS from 1 July 2019.

Appendix 2. Member companies progress to September 2019

Maruha Nichiro Corporation

In July 2018, Maruha Nichiro Group has formed a new Medium to Long term Sustainability Management Plan, ensuring to move towards a corporate group that promotes sustainability in internal corporate values and outward social & environmental development.
In past year we have continued promoting the sustainable use of fishery resources (MSC/ASC), reduce CO2 emissions and reduce waste, and improve recycling rates. We have conducted a cooking class regarding MSC products, have started to tackle plastic issues starting with making survey on the usage of the fishing and farming operations gears and tools related to plastics, how it is handled and preparing the group management regulation. In addition to our work on restoring eelgrass bed in Yokohama, we will restart our coastal clean-up event in coming October. In November Maruha Nichiro is preparing to report for the first time, in line with the Global Reporting Initiative (GRI) guidelines.
In addition, we have participated in the Clean Ocean Material Alliance, a new initiative that aims to promote sustainable use of plastic products and the development and introduction of alternatives to plastics, as well as to accelerate innovations as an effort to solve issues concerning marine plastic debris, a newly emerging global challenge from January 2019.

Nippon Suisan Kaisha (Nissui)

In the past 12 months Nissui has made steady progress regarding relevant themes of SeaBOS. We have newly established Marine Environment & Plastics subcommittee in the CSR Committee which is in direct control of CEO. We have started to attack plastic issue in various ways like considering reduction of usage of plastic in our processed food packages, making survey on the usage and controlling method of gears and tools made by plastic in our fishing or fish farming operations, and conducting clean-up events at river bank of the Arakawa river in Tokyo area whose river basin population is the 2nd largest in Japan. We also participate in the initiative CLOMA (Clean Ocean Material Alliance) which is organized by the Japanese government. Nissui is preparing for the 2nd survey of its procurement of seafood resources of all group companies around the world, covering procurement in 2019. Survey process will be re-started in 2020 for getting result in 2021. GRI is now in introduction stage in Nissui's reporting system and our English web-site content has become more substantial in coverage for improved transparency especially outside of Japan. Nissui is also invited as an Advisory Network member of the High Level Panel for a Sustainable Ocean Economy.

Thai Union

Thai Union has been reporting publicly in line with the Global Reporting Initiative (GRI) guidelines since 2013 and this year for the first time has reported under the Seafood Stewardship Index (SSI).
We provided input to the SSI development, in the belief that greater transparency in the seafood industry is essential to achieving our overarching objectives that the seas are sustainable, our workers are safe and legally employed, and that traceability is the backbone of our sustainability strategy, *SeaChange*. We have continued to be strong supporters of other global initiatives, including the Global Dialogue on Seafood Traceability, and the Global Ghost Gear Initiative. In 2018 Thai Union was ranked number 1 in the world in the Dow Jones Sustainability Index, being industry leaders in topics such as Codes of Conduct, Materiality, Human Rights, Packaging, and Supply Chain Management. Through *SeaChange*, Thai Union is advocating the SDGs, focusing on four main programs including Safe and Legal Labor, Responsible Sourcing, Responsible Operations and People and Communities. We continue to make progress on the agreement signed with Greenpeace in 2017 to drive positive change across the global seafood industry. In 2018, we turned our focus to the seas with the roll out of our Vessel Code of Conduct and Vessel Improvement Program as well as the ongoing focus on 11 tuna Fishery Improvement Projects (FIPs) around the world. Thai Union continued to reduce GHG emission intensity at our manufacturing locations (emissions per unit produced) year on year, working on projects including energy and operational efficiency as well as projects such as solar energy installations.

Mowi

Mowi's commitment to transformational change as part of SeaBOS has continued over the last year. On Task Force I (Reducing IUU fishing and eliminating modern slavery), Mowi participated in the survey to verify feasibility of the voluntary actions proposed to eliminate IUU fishing and modern slavery. On Task Force II (Improving transparency and traceability in global seafood), Mowi met with GDST and WWF to discuss a pilot study to further understand and test the key data elements and inter-operability proposed by the Global Dialogue on Seafood Traceability. On Task Force III (Working with governments to improve regulations), Mowi shared the success case of reduced antibiotic use in the Norwegian salmon farming with the other companies and continues to work on testing a new vaccine in Chile. On Task Force V (Reducing plastic in seafood supply chains), Mowi continues to implement its strategy towards a responsible plastic use which includes setting targets for the first time on this topic. Mowi's new targets are: 1. By 2025, 100% of our plastic packaging will be reusable, recyclable or compostable, 2. By 2025 at least 25% of plastic packaging will come from recycled plastic content and 3. By 2022, 100% of farming equipment is reused or recycled. Mowi also welcomes the establishment of the new Task Force on Climate Resilience and is ready to share its experience on climate accounting, reporting and performance with the other companies. We are happy to announce that seafood in general is doing well regarding sustainability risks and that Mowi has been recognized as the most sustainable animal-protein producer in the world according to the FAIRR (farm animal investment risk & return) benchmark.

Dongwon Industries

Dongwon Industries has been actively engaging with various NGOs to seek possible projects in traceability, transparency and sustainability improvement. In addition to pursuit of MSC certification, Dongwon has been reviewing initiation of various Fishery Improvement Project, which is expected to be initiated within a few months. Once the FIPs initiate, Dongwon Industries will have most of its fishing operations either MSC certified or in FIP. Processing factory in Busan recently received MSC Chain of Custody (CoC) certification and fishing vessels are currently under MSC CoC Audits. To ensure traceability of Bluefin tuna products that we import from Mediterranean farming sites, we've been collecting data to establish a platform that could trace the tuna from end product to farm site, with ultimate goal of putting a QR code on each product in the future. Moreover, in order to reduce plastic usage and improve plastic retrieval, reuse and recycling, Dongwon Industries appointed a Total Plastic Officer to monitor, strategize and manage plastic usage. Various trials and researches on biodegradable FAD (Fish Aggregating Device) is continuing as well to reduce concerns on marine plastic or marine debris. Regarding labor related issues, regular surveys are conducted on all of our crews to check whether various workers' rights are duly protected. Monthly periodicals are sent to our vessels to enhance our communication with crews who are fishing out in the distant sea, and the periodical contains emergency contact information so that crews can contact them at any time in regards to grievances, concerns, or any other issues they may have. Top managements of Dongwon is traveling to our vessels during long holidays such as New Year's Day or Korean Thanksgiving (Chuseok), to have traditional Korean holiday meals with our crews and talk to them in order to listen and find if there is anything the company can do to make any improvements. Although Dongwon Group is reporting in accordance to Global Reporting Initiative (GRI), it has been recently pointed out that the sustainability report of Dongwon Group does not fully capture the seafood related aspects. Dongwon therefore is now working to solve the issue.

Nutreco (owner of Skretting)

In the past year, Nutreco continued to publish its annual sustainability report following GRI compliance, third-party verified and reporting on the prioritized eight SDG aligned with our strategy. This annual sustainability report was nominated a finalist at the UK -RSA accredited Edie Sustainability Leaders Awards. Skretting also published its consolidated annual sustainability report. Nutreco/Skretting were also nominated as a finalist and won the Edie award in Sustainable Innovation Product of the Year for its product Microbalance FLX which is a farmed salmon feed with no marine-based ingredients. Nutreco also designed and coordinated the successful five-month Proof of Concept (PoC) exercise off the west coast of Mexico on a sardine purse-seiner. This PoC used facial recognition and

species recognition software as well as allocating a date/time/position stamp on all data automatically uploaded onto a Unisys blockchain platform. The IT component of this PoC won the Dutch IT Innovation of the Year award. Nutreco also coordinated feedback and revisions from NGOs, WWF and Pew Charitable Trust, on the 45-Criteria in the Voluntary Actions Document

Cargill Aqua Nutrition USA

Cargill Aqua Nutrition applies a value chain approach to sustainability – looking at where the raw materials for our feeds come from and how to ensure they are responsibly produced; how we make the feeds themselves and reducing the environmental footprint of our factories; and how our feeds help our customers develop more sustainable aquaculture and produce delicious and nutritious seafood around the world. Focusing on raw materials, we have increased our study of our suppliers, building on the work in 2018 and continuing to ensure all our suppliers are signed to our Supplier Code of Conduct, which addresses key aspects of environmental and social impacts, including risks associated with child labor and modern slavery. Looking at specific supply chains, we partnered with WWF on our global sourcing of marine ingredients – fishmeal and oil from whole fish and trimmings. This identified that we are well on our way to our 2025 goal of sourcing all material from MSC certified fisheries (in some countries we are nearly at 70% already), but that there are some specific areas that need improving, although the perceived risk of IUU was very low in our supply chains. To this end, we are continuing our work with key Fishery Improvement Projects (FIPs), notably the Peruvian anchoveta fishery and another in Ecuador, as well as two in Thailand. The latter is through our membership of the Seafood Task Force, which also helps to address our risks around labor in fisheries and on land in the seafood supply chain in a high risk region. We also work on key terrestrial supply chains and to develop novel sources of protein and oils, particularly the long chain omega-3 fatty acids EPA and DHA, which are otherwise only available through marine supply chains. Cargill Aqua Nutrition works to support our customers to demonstrate the impact of our nutrition and health feeds on their sustainability, for example through Global Salmon Initiative (GSI) reporting and through BAP and Global GAP certification and compliance with ASC feed requirements pending the launch of their feed standards. We play an active role in the certifications, sitting on advisory committee with ASC and BAP. We also have created a range of health and welfare feeds for salmon and shrimp, which are a keystone for integrated health management in aquaculture and help reduce the need for antibiotics and other medicines.

Overall, this is wrapped up in our annual sustainability report (https://www.cargill.com/sustainability/aquaculture/aquaculture-sustainability-reporting), which continues to be in accordance with GRI Standards as it has for 10 years. Participation in SeaBOS has helped us learn from other members and share our experiences. We now look forward to SeaBOS providing global leadership for the rest of the seafood industry to develop more sustainable practices.

Cermaq (subsidiary of Mitsubishi Corporation)

In the past year, Cermaq has considerably increased its efforts for global multi-stakeholder alignment around SDG 14, most notably by taking a leading role in establishing the Action Platform for Sustainable Ocean Business in the UN Global Compact, which Cermaq co-founded in 2018. Cermaq seconded its Head of Sustainability, Wenche Grønbrekk, to the UN Global Compact in New York the past year to build the initiative, including its innovation and seafood action tracks. A central objective of this work is to provide ocean policy recommendations to governments and the UN from a business perspective. This includes developing global roadmaps and solutions to lift the standard of the fisheries and aquaculture sectors globally. The foundation established is intended to integrate and accelerate SeaBOS contributions in this area. In its operations, Cermaq continues its work to decrease GHG emissions by connecting facilities in Norway to land based electricity run mainly on hydropower, exploring wave energy and integrated farming of seaweed on its facilities in Canada, while it continues to invest heavily in R&D to further strengthen the sustainability of its Chilean operations.

Kyokuyo Co Ltd

Kyokuyo issued the first CSR report compliant with GRI Standard in September 2018, and are now in the process for finalizing the report for 2019. As SDGs, which aims to solve various social issues and achieve a sustainable society, helps develop our ESG-focused business activities, contribute to the society through solving social issues, and realize sustainable improvement of corporate value, we decided to distribute SDGs badges to employees and to wear it in order to instill the idea of SDGs in-house in July 2019. By wearing SDGs badges, we also intend to express our commitment to SDGs to our customers and other stakeholders. In April, in order to solve marine plastic litter issues, Kyokuyo Co., Ltd. became a member of "Clean Ocean Material Alliance" established by the Ministry of Economy, Trade and Industry. This initiative aims to promote sustainable use of plastic products and development and introduction of alternative materials to solve marine plastic litter issues and accelerate innovation. Since April in 2017 we have been a sponsor, as an official partner, for the Japan Canoe Federation, a public interest incorporated association, and held competition, the "Canoe Slalom Japan Cup-Kyokuyo Series" with 7 competitions a year. After each competition, we clean up around the competition course with people who attend for watching competition, athletes, and event officials. We call this activity "Clean River Activity". Since April 2017 total 20 sessions have been held and total participants have come to 1,357 people.

Charoen Pokphand Foods

C.P. Foods strongly believe that being a member of SeaBOS also strengthens our global partnership to achieve the world's 2030 SDG agenda, especially Sustainable Development Goal number 17.
In the past year, C.P. Foods has undertaken global standards such as BAP, IFFO RS and ASC in our shrimp supply chain. In Thailand operation, it is important to note that one-hundred-percent of our fishmeal is sourced from the traceable by-product raw material, which comes from fishmeal plants certified by IFFO RS. And, we are extending our sustainability model & best practices to our operation in other countries such as India, Vietnam and the Philippines. Respect for human rights is our top priority, especially in our labor recruitment process and their welfare practices. Moreover, C.P. Foods has supported the establishment of "Fishermen Life Enhancement Center or FLEC" at the port in Songkhla province in the south of Thailand. The center has both provided basic knowledge to fishing crews on various subjects such as labor rights and improved the livelihood of around 2,000 fishermen and their families. We announced "Sustainable Packaging Policy" to promote responsible use of plastic related to packaging and in our processing lines. We target to use 100 percent of plastic that can be recycled, upcycled and reused in the near future.

Appendix 3. Key collaborators

COMPANY REPRESENTATIVES

Maruha Nichiro
[redacted]

Nippon Suisan Kaisha
[redacted]

Thai Union
[redacted]

Mowi
[redacted]

Dongwon Industries
[redacted]

Nutreco (owner of Skretting)
[redacted]

Cargill Aqua Nutrition USA
[redacted]

Cermaq (subsidiary of Mitsubishi Corporation)
[redacted]

Kyokuyo Co Ltd
[redacted]

Charoen Pokphand Foods
[redacted]

SEABOS SECRETARIAT

[redacted]

SCIENCE PARTNERS

Stockholm Resilience Centre
[redacted]

Royal Swedish Academy of Sciences
[redacted]

The Beijer Institute
[redacted]

Birmingham University
[redacted]

Stanford Centre for Ocean Solutions
[redacted]

University of British Columbia
[redacted]

ADVISORS

Oregon State University
[redacted]

[redacted]

December 2020

SeaBOS

Your Royal Highness, Crown Princess Victoria of Sweden

It is our great pleasure, to present to Your Royal Highness, on behalf of the Keystone Dialogues and the Seafood Business for Ocean Stewardship (SeaBOS) initiative, the following update on our activities.

Please, let us first take this opportunity to thank You for Your inspiring and engaging leadership and passion, and for actively urging us on along our journey, including in the Maldives (Nov 2016), Stockholm (May 2017), New York (June 2017), Karuizawa (Sept, 2018), Bergen (May 2019), and in our "virtual dialogues" (May 2020 and October 2020). We are humbled and indebted to the guidance, facilitation, and continuous support that You provide.

Your passion and longsighted perspective for a healthy ocean provides strong incentives for scientists and SeaBOS members to seriously take on the challenge of ocean stewardship. Your role as an Advocate Alumni for the UN Sustainable Development Goals gives the members even more strength in their work. We very much appreciated having Your kind contributions forwarded to us at our meeting, and hope this outline may help update You on our progress towards creating a transformation to sustainable fisheries and aquaculture production, and a healthy ocean.

Through this unique science-business collaboration, SeaBOS companies are making firm progress towards their commitments and goals (Appendix 1), even with the constraints evidenced this year from covid-19, which had substantial impacts not just on humanity, but also global seafood production and supply chains, including restrictions on movements of crew members, official observers, scientists, agencies, factory and transport workers, and many others associated with seafood. Your Royal Highness, please find more details about progress for the group of SeaBOS companies in Appendix 2, on the science associated to the initiative in Appendix 3. More details from individual member companies can be found in Appendix 4. The individuals involved are listed in Appendix 5 to this report, and a summary of the outcomes from each Task Force of SeaBOS is at Appendix 6.

SeaBOS has firmly established all its legal and policy fundamentals this year, including implementation of a formal 'SeaBOS Association' comprising membership of all the member companies, alongside the SeaBOS Fundraising Foundation which was established in June 2019 in Sweden, and all members have paid their contribution to the foundation in 2020. The new website for SeaBOS at www.seabos.org provides access to all the legal frameworks and policies for transparency, and to assist others who may wish to replicate the model, as well as details about the scientific background associated with the initiative.

During 2020, there were two changes to CEOs leading member companies, with both [redacted] replacing [redacted] replacing [redacted] Both have been active supporters of the model, and continue with their company participation.

The SeaBOS Fundraising Foundation has five Directors, including:

- [redacted]
- [redacted]

- [redacted]

This year, the Foundation elected two replacement Directors, following the resignation of [redacted] (and the Foundation), and the changed role taken on by [redacted] who moved upwards to become the [redacted]. They were replaced by:

- [redacted]
- [redacted]

The work of SeaBOS over recent years has been conducted in six individual task forces, coordinated and supported by the Stockholm Resilience Centre (SRC) at Stockholm University (in collaboration with the Beijer Institute and the GEDB program of the Royal Swedish Academy of Sciences) and the Secretariat. This year, it was agreed to amalgamate the Task Force on Traceability into the Task Force on IUU fishing and modern slavery, and create a new Task Force on Communications. This was in recognition of the fundamental role traceability plays in combatting IUU fishing and modern slavery, as well as the key role that Communications plays in extending the messages from SeaBOS, towards creating the necessary transformations both within SeaBOS companies and their (over 600) subsidiaries, and externally.

We remain connected with global initiatives to harness and enhance their reach, and to accelerate outcomes towards improvements across the spectrum of the Ocean and seafood sustainability. That includes partnerships with the Global Ghost Gear Initiative; the Global Dialogue on Seafood Traceability; the UN Global Compact Action Platform for Sustainable Ocean Business; and active participation in the Advisory Networks as well as providing scientific input to the High-Level Panel for a Sustainable Ocean Economy. And we are intending to extend those partnerships further, in the coming year.

In December, the Ocean Panel, consisting of 14 Heads of State completed and launched their report https://www.oceanpanel.org/ under the banner of "Give it 100%". This report drew together over 250 experts with peer reviewed research, including SeaBOS scientists. We were very fortunate to have the [redacted] where she was able to provide us with insights and expertise to help guide our activities. A number of scientists associated with SeaBOS were involved in producing scientific background reports to the High Level Panel (Appendix 3) which have been presented during the year and integrated in the final report, and these scientists have also been engaged in the High Level Panel Expert Group https://oceanpanel.org/expert-group. SeaBOS companies, as well as SeaBOS as a group, contributed to the Advisory Network https://oceanpanel.org/advisory-network.

SeaBOS members were also part of the launch panels, and there was consistent positive global recognition during those launches, of the potential for SeaBOS to provide leadership in transforming seafood production to being sustainable, and achieving greater ocean health. Their report highlighted five key areas for transformation; being Ocean Health, Ocean Wealth, Ocean Equity, Ocean Knowledge, and Ocean Finance – and there is considerable overlap between the Panel report and recommendations, and the work of SeaBOS, which we will continue to extend and promulgate over coming years.

At the core of our initiative is science, and the collaboration with industry leaders to achieve outcomes based on sound scientific data and analyses. With industry knowledge and expertise, we can create linkages across the multiple sectors and interest groups in ocean governance, and we will continue to do what we can to enhance progress, globally, to achieve the purpose of our Foundation and transform the industry towards ocean stewardship.

As Your Royal Highness is well aware, the SeaBOS initiative, and the identification of its members, is based on a scientific analysis. Scientists from the Stockholm Resilience Centre, the Royal Swedish

Academy, and the Beijer Institute (all based in Stockholm) continue to work closely with SeaBOS members, to ensure they have the science they need to be able to deliver on their commitments. An increased leadership by SeaBOS members of their initiative now means that the responsibility and work load of scientists associated with coordinating and facilitating the work of SeaBOS is in part taken on by the Managing Director (Martin Exel). As a result, the team of scientists increasingly are focusing their efforts on designing an evaluation structure (using insights from organizational science), in drawing out novel scientific insights from the process of facilitating the Keystone Dialogues, and bringing new insights on topic areas covered by the commitments.

The scientific work to date has resulted in the establishment of strong collaborative partnerships between the Stockholm-based organizations, with the University of Birmingham, and Stanford University, among others, and a range of scientific results that when combined, substantially advance the scientific frontiers associated with science-business collaboration. Several additional scientific partnerships are in development. We have published several scientific articles on the role of keystone actors across many ocean sectors as well as on corporate biosphere stewardship (Appendix 3). The wider SeaBOS science team was also influential in the High Level Panel 'Blue Paper' series and [redacted] presented at the 2020 CEO meeting. We find that SeaBOS and the Keystone dialogues inspire others to act, in other sectors and businesses. Hence, the spillover of the SeaBOS lessons is substantial. The role of Your Royal Highness in this context is essential. Looking forward, the science team will deepen their evaluation of SeaBOS itself as well as the idea of Keystone Dialogues as a way address socio-ecological problems and solutions.

Three philanthropic foundations (the Walton Family Foundation, the David and Lucile Packard Foundation, and the Gordon and Betty Moore Foundation) have funded the Keystone Dialogues from their origin, and the SeaBOS initiative to date. All three foundations continue to provide financial support for the science associated with this work. Adequate funding has been obtained until the end of 2021, thereby providing both longevity and flexibility.

We are looking forward to further opportunities to report to Your Royal Highness on our joint efforts; continued progress on transforming the global seafood sector, as well as improving ocean health, and being a source of inspiration for seafood and other sectors.

Sincerely,

[redacted]

[redacted]

[redacted]

[redacted]

Appendix 1. SeaBOS membership and commitments

Ten Keystone actors are members of the SeaBOS Association, including: Maruha Nichiro Corporation, Nippon Suisan Kaisha, Thai Union Group, Mowi, Dongwon Industries, Nutreco/Skretting, Cargill Aqua Nutrition, Cermaq (subsidiary of Mitsubishi Corporation), Kyokuyo, and Charoen Pokphand Foods. The companies operate in wild capture fisheries, feed production, post-harvest production and in aquaculture, and are based in North America, Europe and Asia.

Their commitments are to:

- Improve transparency and traceability in our own operations, and work together to share information and best practice, building on existing industry partnerships and collaborations.
- Engage in concerted efforts to help reduce IUU (Illegal, Unregulated and Unreported) fishing and seek to ensure that IUU products and endangered species are not present in our supply chains.
- Engage in science-based efforts to improve fisheries and aquaculture management and productivity, through collaboration with industry, regulators and civil society.
- Engage in concerted efforts to eliminate any form of modern slavery including forced, bonded and child labour in our supply chains.
- Work towards reducing the use of antibiotics in aquaculture.
- Reduce the use of plastics in seafood operations, and encourage global efforts to reduce plastic pollution.
- Reduce our own greenhouse gas emissions.
- Secure new growth in aquaculture, by deploying best practices in preventive health management, including improved regulatory regimes.
- Collaborate and invest in the development and deployment of emerging approaches and technologies for sustainable fisheries and aquaculture.
- Support novel initiatives and innovations for ocean stewardship.

Appendix 2. SeaBOS progress highlights for 2020

Joint SeaBOS' activities have taken place in six task forces, led in collaboration between multiple member companies, in conjunction with the Stockholm Resilience Centre and its partners, in delivering the evidence-based scientific knowledge for action (see www.seabos.org for more information).

Highlights of SeaBOS progress from this year have been summarised for each Task Force, at Appendix 6, however they include the completion of virtual meetings of the SeaBOS working groups in May where Your participation was much appreciated; and the virtual SeaBOS dialogue in October where Your attendance, support, and enthusiasm for positive outcomes provided considerable inspiration for all. As a reminder, a number of key elements agreed from that October meeting include:

- Agreement on a number of 'time bound goals' towards achieving our commitments, including confirmation that SeaBOS members will, by the end of 2021:
    - Eliminate IUU fishing and forced, bonded and child labour in their own operations – and implement measures to address those issues in their supply chains – with public reporting on progress in 2022 and 2025;
    - Extend their collaboration with the Global Ghost Gear Initiative to solve the problem of lost and abandoned fishing gear; and combine with the International Coastal Cleanup group to remove plastics pollution from our coasts and waterways;
    - Agree on a strategy for reducing impacts on endangered species and the use of antibiotics; and
    - Set CO2 emissions reduction goals and reporting approaches from each company.
- SeaBOS members also finalised two major elements of work from Task Force I towards eliminating IUU fishing and modern slavery: being the voluntary procurement actions outlined at https://seabos.org/wp-content/uploads/2020/12/Voluntary-procurement-actions-Task-Force-I.pdf and also a tool kit which can be used in support of those activities, at https://seabos.org/wp-content/uploads/2020/12/Tool-kit-Task-Force-I.pdf. Both of these outputs from Task Force I have been made publicly available, should others wish to follow the transformative approaches, or learn from SeaBOS activities.
- The work from Stanford Centre for the Oceans to produce a global mapping of risk of IUU fishing and labour abuse was completed (paper in prep.) and this will form a powerful additional risk assessment tool for SeaBOS members to consider when evaluating their supply chains and production of seafood; towards helping focus future efforts to eliminate IUU fishing and modern slavery.
- Our traceability workshop from Task Force II, planned for early in 2020 was unfortunately cancelled due to covid-19, however we ran multiple webinars instead, to demonstrate shared learning, as well as communication of the opportunities for traceability throughout SeaBOS member operations. SeaBOS has continued to support the Global Dialogue on Seafood Traceability, https://traceability-dialogue.org/ who launched their global standards for interoperable traceability systems and key data elements in May this year. Those standards will be used as guidance by SeaBOS members, when adopting new traceability systems.
- After only the first year of work by the Climate Resilience Task Force VI, there was evident alignment of approach from all members. Work in 2020 included a scientific survey of members' operations and impacts from climate change; scientific briefings on the global understandings of Climate Change; as well as shared learning from companies on approaches for climate mitigation. As a result, SeaBOS members took a bold step internationally, when they acknowledged that climate change is having a significant impact on seafood production, and that they can all do their share to mitigate that – through their own emission reduction targets, and advocacy for implementation of the Paris Agreement. The members also highlighted the need for government regulations to support sustainable fisheries and aquaculture management to effectively mitigate climate change risks and impacts, and provide for 'climate smart' seafood production, and healthy protein to help feed the growing population.

- The science survey of antibiotics use by SeaBOS members was presented by webinars, due to cancellation of the workshop planned by Task Force III. It was clear from the assessment and complex issues identified, that a new paradigm was required to identify, and implement, strategies to reduce the impacts of antibiotics on human health. Due to the challenges identified, Members agreed to establish a roadmap by October 2021 to identify ways to significantly reduce and/or phase out from aquaculture operations "High Priority Critically Important Antimicrobials for human health, and Critically Important Antimicrobials for human health" as defined by WHO (https://www.who.int/foodsafety/publications/antimicrobials-sixth/en/).
- SeaBOS reiterated its support under Task Force V to continue using a substantial portion of Foundation contributions to participate with the Global Ghost Gear Initiative, which aims to remove abandoned, discarded, and lost fishing gear from our Ocean. This partnership was extended to include a coordinated plastics pollution clean-up campaign in 2021, with the aim being to both help reduce some of the plastic pollution in our coasts and waterways, but also to highlight and communicate the dire need to remove plastic pollution from our ocean, through SeaBOS members and their subsidiaries, as well as to external parties.
- Extensive administrative and legal links between science, industry, and Foundations were formalized this year, to ensure we have a sound fundamental governance structure into the future. Members thanked Chairman Shigeru Ito for his leadership and guidance over the past two years in his role as inaugural Chair of the SeaBOS Association, and elected for two-year terms, [redacted]
- We are planning further webinars and updates for 2021, including in particular the use of Fishery Improvement Programs to help build capacity in areas identified as at high risk for IUU fishing and/or modern slavery, as well as increased sustainability for wild catch and aquaculture, and greater insights on ways to reduce fishery impacts on endangered species.
- Most recently, we have agreed to establish a 'meta-coalition' with the Global Tuna Alliance, and the International Sustainable Seafood Foundation with support of the Friends of Ocean Action, to work jointly to encourage governments at the APEC meeting in late 2021, to implement fully the Port State Measures Agreement towards eliminating IUU fishing. It is also intended to trial as many of the Task Force I voluntary measures and tool kit approaches as possible, to demonstrate how they can be used 'at scale' towards achieving transformative outcomes. The coalition will continue to endorse the Global Dialogue on Seafood Traceability standards and key data elements, throughout the seafood supply chain, as well as explore additional methods to extend transparency yet further for seafood production. A key issue in past actions on traceability has been the challenge of maintaining robust alignment along the full supply chain, and it is hoped that trials with the meta-coalition may be able to demonstrate how this can be achieved in future. Funding and policy encouragement for the program is also being provided from the US Foundations who contribute finances to SeaBOS science. A formal announcement of the new meta-coalition and its goals, will be made public in late January, 2021.

Appendix 3. Scientific progress during 2020

The scientific work associate to the keystone dialogues and the Stockholm Resilience Centre, the Beijer Institute of Ecological Economics, and the Royal Swedish Academy of Sciences, has contributed to develop a unique branch of transdisciplinary science, in collaboration with global corporations. Key partners in this work include the University of Birmingham and Stanford Centre for Ocean Solutions. The most relevant papers published by these scientists are listed below, organized under four headings: *Understanding transnational corporations, Learning from science-business cooperation, Investigating Anthropocene dynamics, and Background papers to the High Level Panel*

Understanding transnational corporations

- Carmine, G., Mayorga, J., Miller, N.A., Park, J., Halpin, P.N., Ortuño Crespo, G., Österblom, H., Sala, E., Jacquet, J. (2020) Who is the high seas fishing industry? *One Earth* https://www.cell.com/one-earth/pdfExtended/S2590-3322(20)30607-2
- Virdin, J., Vegh, T., Blasiak, R., Jouffray, J.B., Mason, S., Österblom, H., Vermeer, D., Werner, N. (2021). The Oceans 100: Transnational Corporations in the Ocean Economy. *Science Advances*

Learning from science-business cooperation

- Österblom, H., Cvitanovic, C., van Putten, I., Addison, P. Blasiak, R., Jouffray, J.-B., Bebbington, J., Hall, J., Ison, S., LeBris, A., Mynott, S., Reid, D., Sugimoto, A. (2020) Science-industry collaboration – sideways or highways to ocean sustainability? *One Earth* 3: 79-88. https://www.sciencedirect.com/science/article/pii/S2590332220303006
- Österblom, H., Bebbington, J., Folke, C., Blasiak, R., Crona, B., Henriksson, P., Jouffray, J-..B. Merrie, A., Rockström, J., Schultz, L., Selig, E., Spijkers, J., Troell, M., Wabnitz, C. (2020) 海洋管理に向けた水産事業 (SeaBOS) の取り組み –発足の経緯と初期成果 [The Seafood Business for Ocean Stewardship (SeaBOS) initiative : Origin, Development and First Results] *Environmental Information Science* 49, 72-82
- Norström, A.V., Cvitanovic, C., Löf, M.F., West, S., Wyborn, C., Balvanera, P., Bednarek, A.T., Bennett, E.M., Biggs, R., de Bremond, A., Campbell, B.M., Canadell, J. G., Carpengter, S.R., Folke, C., Fulton, E.A., Gaffney, O,. Gelcich, S., Jouffray, J.-B., Leach, M., LeTissier, M., Martín-López, B., Loutre, M.-F., Meadow, A.M., Nagendra, H., Payne, D., Peterson, G., Reyers, B., Scholes, B., Speranza, C.I., Spierenburg, M., Stafford-Smith, M., Tengö, M., vad der Hel, S., van Putten, I., Österblom, H. (2020) Principles for knowledge co-production in sustainability research. *Nature Sustainability* 1:9 https://www.nature.com/articles/s41893-019-0448-2

Investigating Anthropocene dynamics

- Brodie Rudolph, T., Swilling, M., Ruckelshaus, M. Allison, E.H., Österblom, H., Gelcich, H., Mbatha, P. (2020) A transition to sustainable ocean governance. *Nature Communications* 11: 3600. https://www.nature.com/articles/s41467-020-17410-2
- Jouffray, J.-B, Blasiak, R., Norström, A.V., Österblom, H., Nyström, M. (2020) The Blue Acceleration: The Trajectory of Human expansion into the ocean. *One Earth* 2(1) 43-54. https://www.cell.com/action/showPdf?pii=S2590-3322%2819%2930275-1
- Crona B, Wassénius E, Troell M, et al (2020) China at a Crossroads: An Analysis of China's Changing Seafood Production and Consumption. *One Earth* 3:32–44. https://www.cell.com/action/showPdf?pii=S2590-3322%2820%2930302-X
- Wernli, D., P.S. Jørgensen, E.J. Parmley, M. Troell et al. 2020. Evidence for action: A One Health learning platform on interventions to tackle antimicrobial resistance. *Lancet Infect. Dis.* 20: e307-e311 https://www.thelancet.com/action/showPdf?pii=S1473-3099%2820%2930392-3
- Jørgensen, P. S., C. Folke, P. J. G. G. Henriksson, K. Malmros, M. Troell, A. Zorzet, and Living with Resistance project. 2020. Coevolutionary Governance of Antibiotic and Pesticide Resistance. *Trends in Ecology and Evolution*: 36: 484-494. https://www.cell.com/action/showPdf?pii=S0169-5347%2820%2930013-6

Background papers to the High-Level Panel (Blue Papers)

- Swilling, M., Ruckelshaus, M., Brodie Rudolph, T. Allison, E.H., Gelcich, S., Mbatha, P., Österblom, H. 2020. The Ocean Transition: What to Learn from System Transitions. Washington, DC: World Resources Institute. https://www.oceanpanel.org/blue-papers/ocean-transition-what-learn-system-transitions
- Österblom, H., Wabnitz, C.C.C. Tladi, D., Allison, E.H., Arnaud-Haond, S., Bebbington, J., Bennett, N., Blasiak, N., Boonstra, W. Choudhury, A., Cisneros-Montemayor, A., Daw, A., Fabinyi, M., Franz, N., Harden-Davies, H., Kleiber,D., Lopes, P., McDougall, C., Resosudarmo, B.P. Selim, S.A. 2020 Towards Ocean Equity. Washington, DC: World Resources Institute. https://www.oceanpanel.org/sites/default/files/2020-04/towards-ocean-equity.pdf
- Blasiak, R., R. Wynberg, K. Grorud-Colvert, S. Thambisetty, et al. 2020. The Ocean Genome: Conservation and the Fair, Equitable and Sustainable Use of Marine Genetic Resources. Washington, DC: World Resources Institute. https://www.oceanpanel.org/blue-papers/ocean-genome-conservation-and-fair-equitable-and-sustainable-use-marine-genetic
- Sumaila, U.R., M. Walsh, K. Hoareau, A. Cox, et al. 2020. Ocean Finance: Financing the Transition to a Sustainable Ocean Economy. Washington, DC: World Resources Institute. https://www.oceanpanel.org/blue-papers/ocean-finance-financing-transition-sustainable-ocean-economy

Appendix 4: Member companies' progress in 2020

Cermaq

Cermaq continues its commitment to driving progress and building awareness around the central role of sustainable seafood in the 2030 Agenda, through its partnerships with the UN Global Compact, the Ocean Panel, the FAO and in food systems dialogues.

The past year, Cermaq led the working group that launched the UNGC Aquaculture Guidance to the Sustainable Ocean Principles in September this year, the first of its kind. Cermaq has the past year mapped its GHG emissions and is in the process of establishing a climate target.

Cermaq continues investing in sustainable farming technologies and took an important step forward by stocking its iFarm sites in Norway in September. iFarm aims to develop image recognition for identification of each individual salmon and individual follow-up of each fish. If successful, iFarm be a game changer for sustainability and animal welfare.

Mowi

Our big goal is to produce more food from the ocean for a growing world population in a way that respects the ocean's assimilative capacity, allows local communities to flourish while offering consumers products that are tasty, healthy and of the highest quality. In combination, this will ensure long-term profitability.

Mowi's Sustainability Strategy, *Leading the Blue Revolution Plan* (https://corpsite.azureedge.net/corpsite/wp-content/uploads/2020/06/Mowi-Sustainability-Strategy.pdf, has further been implemented in 2020. Our sustainability strategy underpins commitments across our social and environmental performance through the value chain. Our commitments are set to make our business future-proof and are aligned with the UN Sustainable Development Goals. During 2020, Mowi has strengthened its commitment to risk-assess suppliers by starting to implement a global onboarding and risk-assessment tool which will include human rights screening. This comes in addition to our already implemented Code of Conduct for suppliers, audits and adhering to recognized certification schemes. Issues like modern slavery and child labour will be screened throughout this process.

On traceability, Mowi continues its engagement with the Global Dialogue on Seafood Traceability and running a pilot testing with one of the largest UK retailers. This activity comes in addition to our already implemented traceability systems. On antimicrobial use, Mowi continues to implement its policy on responsible us of antimicrobials and apply biosecurity measures in the different farming countries to minimize the use of antibiotics (https://corpsite.azureedge.net/corpsite/wp-content/uploads/2020/03/Mowi-Policy-on-use-of-antimicrobial-agents.pdf).

In 2020, Mowi continued its efforts to use plastics in a responsible way (https://corpsite.azureedge.net/corpsite/wp-content/uploads/2020/02/Mowi-Plastics-Policy.pdf). We have increased the share of farming equipment that is either re-used or recycled and use alternative packaging solutions that are designed for material reduction, reuse, recycling and/or composting. Finally, on climate resilience, Mowi strongly believes that producing more food from the ocean will play a key role on offering a climate-friendly alternative protein to consumers, an alternative that is both healthy and with a low carbon footprint. We have set Science Based Targets for GHG emissions reduction and are running key initiatives across our value chain to reduce our emissions (https://corpsite.azureedge.net/corpsite/wp-content/uploads/2020/02/Mowi-Climate-Change-and-Energy-Use-Policy.pdf). This includes dialogues with our feed raw material suppliers, increase the use of renewable electricity and find alternative to reduce our dependency on fossil fuels. All in all, Mowi is fully committed to be part of transformative change to ensure we continue to unlock the potential of our ocean in a sustainable way.

Kyokuyo

In February this year, we formulated "Kyokuyo Group Basic Procurement Policy" for building a sustainable society through responsible procurement. In order to achieve the goal, it is required to

work with our suppliers in procurement activities. Now, we are preparing supplier guidelines on the basis of Kyokuyo Group Basic Procurement Policy, and going to ask our suppliers for understanding of our policy and cooperation for responsible procurement, which supports achievement of sustainable society. We believe that we can move forward in eliminating IUU fishery products and forced labor in our supply chain by deploying our supplier guidelines to our business partners.

We have been continuously increasing handling/registered number of MSC certified species in our business. Up to the moment, it comes to 33 species. We are going to increase handling number of MSC certified species in future, as well.

Last year, we launched aquaculture of other species in addition to blue fin tuna, such as sea bream and amber jack. We are now under preparation for obtaining certificate of marine eco-label for this new aquaculture business. By above measures, we are continuing to make effort for increasing procurement of seafood from properly managed marine resources.

Besides above, as a part of the internal communication, we provided almost every employees of Kyokuyo Group companies in Japan with e-learning for their better understanding of our group's activities and mindset on sustainability including MSC/ASC and SeaBOS.

NISSUI

In the past year NISSUI has made steady progress in various themes which are related to SeaBOS activities. In Jun 2020 we announced in public NISSUI Group Basic Policy regarding the problems of Plastics which shows our position towards Plastic issue. NISSUI is now preparing our Group's consolidated Human Right Policy and hopefully it will be established before long.

CSR procurement policy has been also implemented and we have started to apply it to our major suppliers step by step. We believe these establishments will help us push forward the activity to proceed IUU and Forced Labor free procurement in our supply chain from now on.

NISSUI has the Marine environment & Plastic subcommittee in our CSR Committee and in this subcommittee we have checked current usage off plastic fishing gears(in both fisheries and aquacultures) and way of control them in operations of our group companies. We have got relevant information or some advice from Global Ghost Gear Initiative which helped us a lot in proceeding our work.

NISSUI has started to introduce GRI standard to make our CSR report and we have also improved the contents of English version of our Company Report or CSR Report so that we can transmit our information to all over the world and this could improve our transparency to our stakeholders abroad.

The 2nd survey of our procured marine products is now underway. Data of all procured marine products in our group companies in 2019 has already been collected and we are going to check sustainability of fish stock of each marine product. Using these information we have collaborated with the research team of Stanford University about their Risk Mapping Project.

NISSUI continues to be a member of Advisory Network of the High Level Panel for a Sustainability Ocean Economy.

Dongwon Industries

During the past year, Dongwon reached several milestones in our journey towards sustainable fisheries and oceans.

In order to address Protein Challenge 2050, we took our first step into the aquaculture business. Although Dongwon takes a part of the business as a shareholder, we seek to expand our expertise and presence in the field through this opportunity.

We achieved 2 different MSC fishery certifications along with CoC certification, through which Dongwon became one of the first companies to hold multi-gear MSC certification. Three Fishery Improvement Projects are under way as well, two being already launched in the Pacific and Atlantic Ocean.

We’ve also established detailed plans to reuse, recycle and reduce plastic within our own operation. Our goal of reducing plastic with specific figures was publicly announced, covered by various media sources. Some of the examples include converting from conventional FADs to bio-degradable FADs and substituting 2-litre bottled water with 20-litre water containers, which are supplied to our crews as a part of the crew welfare improvement. The initial plan spans out throughout 2020~2022 but we seek to extend the project beyond 2022 after reevaluating our achievement at the end of the timeline. Total Plastic Officer (TPO) was designated to carry out this specific task.

Crew management has been strengthened as a result of constant monitoring of crew welfares. Top managements and crew management team also visit our vessels on a regular basis to conduct field inspection on our crew welfare management, which includes interviews with the crews and sharing words of encouragement. Regular surveys have been conducted on all of our crews since 2016 and as a result we’ve been making significant improvements throughout the years and during the past year especially, we focused on whether crews were duly paid of their salaries electronically in full, they are aware of their contract conditions, their grievances are addressed in a proper manner, their breaks and rests were duly guaranteed, and rights are well protected under Dongwon’s umbrella. Also, duration of a contracted embarkation period has been shortening over time in order to reduce fatigue of our crews. However, the contract duration differs by each individual based on their preferences.

Lastly, we are currently looking to implement traceability system that can be used and accessed by end-consumers. Although ideas are still being developed, we hope to see the results soon.

Maruha Nichiro Corporation

In the past year, Maruha Nichiro has published its integrated report for the first time in line with the Global Reporting Initiative (GRI) guidelines. In coming weeks we will publish the 2020 report. This year for the first time we will prepare the report in English on our website.

We have continued promoting the sustainable use of seafood resources (MSC/ASC), reduce CO2 emissions and reduce waste, and improve recycling rates. We have conducted a cooking class using MSC products, continued to tackle plastic issues on the usage of the fishing and farming operations’ gears and tools related to plastics, how it is handled and preparing the group management guidelines. We have conducted coastal clean-up event in line with International Coastal Cleanup group.

The acquisition of "ASC certification" for amberjack in July 2019 was the first case certified in the world. In addition with joint test research among,” industry, government and academic institutes”, we have received ASC certification for land based farming of cherry salmon in March 2020. This was the first case in Japan of acquiring ASC-certified salmon by land-based aquaculture.

We have conducted two surveys for “marine resources” and “supplier assessment”. Hope to share our results starting from 2021. Continued our participation in the Clean Ocean Material Alliance, UN Global Compact, and got in touch with the High Level Panel for a Sustainable Ocean Economy.

Thai Union

Thai Union has continued to support the Sustainable Development Goals, and has recently received the SDG Impact Award at the Responsible Business Awards 2020. As we come to the end of the first period of our SeaChange sustainability strategy, we have been looking back at our progress, as well as looking forward to the challenges for the next five years to address in SeaChange 2025. We are proud to report that in the middle of 2020 Thai Union met its global Tuna Commitment, to establish 11 Fishery Improvement Projects in tuna fisheries around the world, and ensure that 75% of its branded tuna is sourced from either a FIP or from a MSC certified fishery.

We have continued our strong collaboration with the Global Dialogue for Seafood Traceability, and are implementing trials for the transfer of the Key Data Elements using the interoperability framework. We have continued our program to install Electronic Monitoring on the tuna vessels from which we source, working with The Nature Conservancy to bring greater transparency to the seafood sector. We continued to implement our Vessel Code of Conduct and Vessel Improvement Program in both the

international tuna fleet, as well as the Thai fleet from which we source. We transparently reported on findings of the audits in our 2019 Sustainability Report, recognizing that to admit to finding where there are problems with IUU fishing and modern slavery in supply chains is the first step to finding solutions. One example of solutions has been to work with the International Transport Workers Federation (ITF) to deliver Health and Safety training to fishers in Thailand.

Our work with the Global Ghost Gear Initiative (GGGI) has also continued, as we implement the activities in our Workplan. After a very successful Glost Gear Dive in June 2019, we have started working with fishermen at ports throughout Thailand to understand what they see as the challenges with the recovery of ghost gear and map the issues. In March 2020 we signed up to EP100, The Climate Group's global initiative on smarter energy use. As part of our strategy to tackle climate change and its impact on the world's oceans, Thai Union is aiming to double its energy productivity by 2041, from a 2016 baseline. We were the first food company, and first company in Thailand to join EP100.

Our work on ethical recruitment and human rights continues to be a key platform for us and our workers in our own facilities and supply chains. We introduced a global third-party whistleblowing hotline, Navex, for all staff across Thai Union sites globally. The independent review of our Ethical Migrant Recruitment Policy by Impactt, published in 2019, has helped us refine and define our ethical recruitment journey for the next five years, and we continue to collaborate with other partners on platforms to help improve worker voice mechanisms and utilize technology for positive change.

Cargill Aqua Nutrition

Cargill Aqua Nutrition is exposed to many markets through its global operations and many of our suppliers and customers have been heavily impacted by the global COVID-19 pandemic. However, over the last 6 months we have been able to continue to work with our value chains on a variety of sustainability projects despite the difficulties, reflecting the great importance we all attach to these issues – which is also highlighted in our annual sustainability report for 2019 (link).

In fisheries, we have worked with the team from Stanford University to envisage how companies could use their risk assessment maps on IUU fishing and labour abuses – an incredible resource which can help us to identify and really address these issues at source. But we are also working with several fishery improvement programs to improve the management of the target fisheries. In particular, our 4-year engagement with the Peruvian anchovy fisheries in north-central Peru looks like it will soon conclude, with the largest fishery in the world now ready for full assessment to MSC! This is a huge achievement, showing that complex fisheries can be sustainably managed. During the same period, we are also working hard in Europe to find a solution for them to establish quotas to ensure future sustainable management practices for mackerel, Atlantic herring and blue whiting. Cargill Aqua Nutrition has led work to raise awareness of the issues and we are now partnering with NAPA (North Atlantic Pelagic Advocacy group) on an advocacy program to the relevant regulators.

We are using SeaBOS' link to GDST to work on traceability in our marine ingredients supply chain, to see how we can receive data directly from our supplier that will detail the origin of the fish in each batch of fishmeal and oil we receive. This data will be analysed and can be shared with our value chain, to help demonstrate how sustainable our supply chains are, not just to our customers but right down to the consumer level if needed. We represented SeaBOS in a digital conference, Seafood 2030, to talk about the importance of traceability in the seafood sector – both from addressing illegal activities and from encouraging sustainable management.

Looking forwards with SeaBOS, we are pleased that the level of engagement in Task Force III will increase, as we believe that the issue of antimicrobials is very important to the aquaculture industry, but it is very detailed and will be a big challenge to address effectively. Whilst aquaculture is currently a relatively low emitter of GHG, we can always do better, and must, to ensure that we can grow our industry sustainably.

Nutreco (owner of Skretting)

In the past year, Nutreco continued to publish its annual sustainability report following GRI compliance, third-party verified and reporting on the prioritized eight SDG aligned with our strategy. This annual sustainability report was nominated a finalist and awarded "Honorable Mention" at the accredited 2020 Reuter's Responsible Business Awards. Skretting also published its consolidated annual sustainability report. Nutreco/Skretting adopted its newly developed sustainability strategy entitled, RoadMap 2025, where sourcing sustainably produced marine ingredients and rigorous targets addressing AMR and sustainable packaging are clearly and specifically addressed.
After over a decade of failed attempts to engage the Peruvian anchoveta fishery in a comprehensive FIP towards ultimate MSC assessment, Skretting, in partnership with Cargill Aqua Nutrition and the local fishing association SNP, successfully completed a three-year comprehensive FIP which will enable the fishery to apply for MSC full assessment.

In April 2020 Nutreco/Skretting joined 985 other private companies by publicly committing to ambitious, yet realistic, CO2 reduction targets in alignment with the 2015 Paris Agreement guidelines [1]. As aquaculture feed trends continue to focus on reducing the inclusion of fishmeal, the popular replacement commodity is soy and soy by-products. This industry trend is fueling expansion of soy production in South America and the associated deforestation of virgin forests. Nutreco/Skretting have addressed this by being one of three companies pledging funds to "payment for environmental service" -type initiative with the development of The Cerrado Funding Coalition [2].

CP Foods

In the past year, CP Foods has continued to promote global standards such as BAP, MarinTrust RS (previously named IFFO RS) and ASC in our shrimp supply chain. In Thailand operation, it is important to note that one-hundred-percent of our fishmeal is sourced from the traceable by-product raw material, which comes from fishmeal plants certified by MarinTrust RS.

We have collaborated with Thai Sustainable Fisheries Roundtable (TSFR) in the name of Thai Feed Mill Association in trawler fishing projects in both the Gulf of Thailand and the Andaman Sea. In the Gulf of Thailand, we have adopted the MarinTrust RS Version 2.0, the latest version, for GAP analysis and assessment. We also consistently collaborate with the MarinTrust to develop assessment guidelines for mixed trawl fisheries management to pilot and initiate the first sustainable fishery standard in the world that is applicable to the Southeast Asian region. We are pleased to report that Fishery Action Plan-FAP for the Gulf of Thailand, has been recently approved by MarinTrust Governing Body Committee. This is the first multispecies Fisheries Improvement Project-FIP and is paving the way towards sustainable fishery for other oceanic zones with similar fishery characteristic.

For labour aspect, we have reviewed and conducted the Human Rights Due Diligence Process, covering all groups of stakeholders, including vulnerable groups such as ethnic or religious minority groups, disabled group, and children. The process integrated within all the business units in Thailand.

We have continued to support "Fishermen Life Enhancement Center or FLEC" at the port in Songkhla province in the south of Thailand. Main aims are to eradicate illegal labor in vessels, leverage quality of life of fishery laborers and their families. The efforts resulted in concrete outcomes of the country's reputation on labor issues and expansion of networks to avoid future problems. In the past year, FLEC has provided labor practices related training and consultations on healthcare, rights and welfare, services in nursing room and first aid kit on vessels to 2,089 fishermen.

As per the announced Sustainable Packaging Policy last year, we now commit that 100% of plastic packaging for food products to be reusable or recyclable or upcyclable, or compostable by 2025 and 2030 using innovative packaging design and reducing unnecessary plastic packaging throughout the value chain.

Appendix 5. Key collaborators

COMPANY REPRESENTATIVES

Maruha Nichiro
[redacted]

Nippon Suisan Kaisha
[redacted]

Thai Union
[redacted]

Mowi
[redacted]

Dongwon Industries
[redacted]

Nutreco (owner of Skretting)
[redacted]

Cargill Aqua Nutrition USA
[redacted]

Cermaq (subsidiary of Mitsubishi Corporation)
[redacted]

Kyokuyo Co Ltd
[redacted]

Charoen Pokphand Foods
[redacted]

SCIENCE PARTNERS

Stockholm Resilience Centre
[redacted]

Royal Swedish Academy of Sciences
[redacted]

The Beijer Institute
[redacted]

Birmingham University
[redacted]

Stanford Centre for Ocean Solutions
[redacted]

Oregon State University
[redacted]

SEABOS SECRETARIAT

[redacted]

Appendix 6: Summary outcomes from the 2020 Keystone Dialogue

Task Force I: IUU fishing and modern slavery ([redacted] lead from Nutreco, Dongwon, Maruha Nichiro, CP Foods, Stanford, Birmingham, and SRC)

AGREED TO:

1. Have no IUU fishing products or modern slavery in our own seafood operations by Oct 2021; and to
2. Put science-based measures in place that, when combined, substantially reduce the risk of IUU fishery products or modern slavery being in our supply chains. These measures and their associated time plan for implementation, will be publicly announced by SeaBOS by 31 December 2020; and
3. In recognition that IUU fishing and modern slavery are endemic within the global seafood industry and require continuous vigilance by all actors, we will act swiftly and transparently on any evidence that these activities exist within our operations and/or supply chains. Taking these challenges in to account, we are convinced that the science-based measures that will be put in place, along with continued policy engagement, will support the elimination of both IUU fishing and modern slavery in our supply chains. We will report in October 2022 and October 2025 on progress towards meeting our goals.

AGREED to refine 'modern slavery' definition to being 'forced, bonded, or child labour'

AGREED to participate in a meta-coalition to promote PSMA, traceability, transparency, and SeaBOS science-based solutions;

NOTED the effective science-based solutions developed by Task Force I with scientific support;

SUPPORTED science work to provide 'best practice' examples for minimising impacts on endangered species by Oct 2021;

AGREED to recommend clear time-bound goals on minimising impacts on endangered species for approval by CEOs by Oct 2021; and

INCORPORATED Traceability from Task Force II, into the work of Task Force I (with identified person in charge).

Task Force II: Traceability ([redacted] from Thai Union, Nutreco, Royal Swedish Academy of Science)

AGREED TO:

- Continue collaboration with Global Dialogue on Seafood Traceability; supporting adoption of those standards as appropriate; recognising critical need for full supply chain involvement; and supporting continued completion of GDST surveys by members, along with voluntary trials and shared learning of experiences with implementation of the standards by some companies.
- Incorporate Task Force II into Task Force I (with an identified company member nominated to remain lead on traceability) given fundamental nature of traceability in work to prevent IUU fishing and modern slavery

Task Force III: Working with governments and AMR ([redacted] lead from Cargill Aqua Nutrition, Nissui, Dongwon, CP Foods, SRC, Beijer)

RECOGNISED there are local, national, regional, and international variations in regulations and requirements on the use of different antibiotics for fish health;

NOTED that there is clear evidence that human health and wellbeing relies on the reduction of usage for particular antibiotics, as soon as possible; and

NOTED the desire to identify opportunities and develop innovative solutions to redress the challenges of antibiotic reductions for particular species, and regions; and

AGREED to establish a roadmap by October 2021 to identify ways to significantly reduce and/or phase out from aquaculture operations "High Priority Critically Important Antimicrobials for human health, and Critically Important Antimicrobials for human health" as defined by WHO (https://www.who.int/foodsafety/publications/antimicrobials-sixth/en/). As part of that process, SeaBOS will:

- **Develop** a recommended Code of Conduct for antibiotic use including preventive practices, recommended therapeutic treatments, and collaborative, pre-competitive Research & Development by October 2021; and
- **Contribute** with higher resolution data enabling refinement of the conducted antibiotic survey of SeaBOS members; and
- **Establish** collaborations and exchanges with relevant expert organisations towards identifying alternative approaches.

AGREED to continue collaborations with governments and inter-governmental groups such as the UN Global Compact Action Platform for sustainable ocean business; the High-Level Panel for sustainable ocean economy; and others.

Task Force IV: Transparency and Governance ([redacted] from Cermaq and [redacted] from Nissui as joint leads, SRC, Birmingham)

FINALISED administrative and legal links between science, industry, and Foundations and **AGREED**:

- Principles for SeaBOS engagement;
- Research protocol for SeaBOS (as updated with legal advice);
- SeaBOS Secretariat and SRC Operational MOU:
- Anti-trust policy as updated with legal advice

THANKED [redacted] for his leadership and guidance over the past two years, as inaugural Chair of SeaBOS

ELECTED, for two-year terms:

- [redacted];
- [redacted] (and **RECOMMENDED** he replace [redacted] at the Fundraising Foundation board);
- All CEOs of the remaining eight companies as Board members;
- [redacted] as members of the New Member Selection Committee;

APPOINTED [redacted] as Managing Director;

AGREED to hold the next annual SeaBOS Association meeting in Amsterdam from Sunday evening 3 October to end of Tuesday 5 October 2021;

NOTED that SeaBOS Fundraising Foundation received an unqualified audit report for the 2019;

RECOMMENDED the Fundraising Foundation extend the insurances at the existing levels of cover for General Liability and Product Liability as well as business travel;

RECOMMENDED an increase in cover when extending the Directors and Officers Liability insurance to 25 million SEK (approx. $2.85M USD) for any one claim;

RECOMMENDED continuation of existing SeaBOS policies and guidelines

- Rules of procedure for the Board of Management of the SeaBOS Association
- Travel and expenses
- Workplace ethics, health and safety
- Anti-trust policy as updated with legal advice;

RECOMMENDED a 2021 budget for the SeaBOS Fundraising Foundation with expenditure estimated at $522,000 USD;

NOTED a positive update on support from USA Foundations for the work of SeaBOS; and

AGREED to report on new membership options by October 2021.

ESTABLISHED a 'Communications Task Force'

- Membership to be advised
- Strategy to October 2021 Keystone Dialogue; and

NOTED the need to develop a sustainable funding and structure model by October 2021.

Task Force V: Plastics ([redacted] from Thai Union, Mowi, Kyokuyo, SRC)

ESTABLISHED a 'City to Sea' framework;

RECOMMENDED:

- Focus to be on areas where seafood businesses can have greatest impact;
- Continued membership of GGGI at cost for the 2020/2021 year of $100,000USD;
- Having an annual coordinated International Coastal Cleanup process for SeaBOS members and their subsidiaries by September 2021 at estimated cost of up to $25,000USD;

AGREED:

- To raise our concerns with both governments and civil society, that plastics are an issue in our ocean but also emphasize that sustainable seafood, a healthy and nutritious form of protein, is reliant on ocean health
  - This would be as part of our new communications strategy, and could incorporate scientific studies and outputs on microplastics in seafood.
- To provide at least biennial reporting on plastics packaging footprints, along with shared learning webinars during 2021 on innovative solutions to make plastics lighter; re-use, reduce, recycle, or make plastics compostable.

Task Force VI: Climate Resilience ([redacted] lead from Mowi, Maruha Nichiro, Cermaq, Cargill Aqua Nutrition, SRC, Royal Swedish Academy of Science, Beijer)

AGREED:

- companies would establish science-based goals and reporting approaches for reduction of greenhouse gas emissions by October 2021;
- to include the SeaBOS climate resilience/leadership in the strategy for our new communications task force;
- to support a SeaBOS Association statement that highlights:
  - the impacts of climate change on seafood production;
  - the actions we are taking individually and collectively to mitigate our greenhouse gas emissions;
  - the need for government regulations to support flexibility and capability to effectively mitigate climate change risks and impacts, and provide for 'climate smart' sustainable seafood production; and provides
  - recognition of the benefits of sustainable seafood as a healthy, climate friendly protein source to feed our increasing world population; and
  - Continued collaboration with groups supportive of SeaBOS goals towards improved regulations and approaches to deal with climate change impacts on sustainable seafood production

Stockholm Resilience Centre
Sustainability Science for Biosphere Stewardship

Stockholm University

Beijer Institute
OF ECOLOGICAL ECONOMICS

KUNGL. VETENSKAPS-AKADEMIEN
THE ROYAL SWEDISH ACADEMY OF SCIENCES

GLOBAL ECONOMIC DYNAMICS AND THE BIOSPHERE
THE ROYAL SWEDISH ACADEMY OF SCIENCES

UNIVERSITY OF BIRMINGHAM

Stanford | Center for Ocean Solutions

WALTON FAMILY FOUNDATION

the David & Lucile Packard FOUNDATION

GORDON AND BETTY MOORE FOUNDATION

CPF

MARUHA NICHIRO

CERMAQ

NISSUI

MOWI

nutreco

KYOKUYO

Dongwon

Cargill

Thai Union

WALTON FAMILY FOUNDATION

the David & Lucile Packard FOUNDATION

GORDON AND BETTY MOORE FOUNDATION

Supplementary Data S3: Research Protocol for SeaBOS.

Research protocol for SeaBOS

SeaBOS is a novel way for science and business to interact and learn from each other, while also advancing knowledge and action. The aim of this collaboration is to investigate if industry leaders, with support from science, can stimulate large-scale, transformative change towards ocean stewardship.

This document aims to clarify the relationship between science and business in this process, which requires two different modes of research, namely:

1) scientific support for achieving progress – *Science for SeaBOS;* and
2) scientific monitoring to document progress – *Science of SeaBOS*. The rationale and practical conditions for each mode of research are described below.

1. Science for SeaBOS

This mode of research primarily provides SeaBOS members with knowledge that supports the implementation of commitments made by companies. For instance, how can a commitment to eliminate modern slavery be enabled, what is the state of the art of existing knowledge and practice, what new knowledge is necessary, and what has worked before or in other sectors? This mode of research supports the operational science-business interaction that takes place in individual task forces. The primary place for presenting results is through background briefs presented at SeaBOS meetings and on the associated web-page. Scientific publications are also likely to emerge from this work.

Science for SeaBOS requires that:

1. The research task is specified according to the dual criteria of scientific rigour and business needs (aligned to SeaBOS commitments);
2. The required capacity is defined, including any funding or other resources;
3. The research design is clear, for instance the definition of roles and responsibilities of SeaBOS members and SRC (and scientific partners), including an agreement on timelines.

The principles that apply are:

1. Work will be co-designed (following the same spirit of the Keystone Dialogue process).
2. Research ethics (and especially issues of confidentiality) are addressed clearly.
3. Legal and commercial issues arising from the research are addressed clearly.
4. The research team will be able to use material from the work (subject to confidentiality) in developing original research.

General specification (Science for SeaBOS):

Sourcing the research need	Funding and other resources	Design and logistics
There are three potential sources of research needs:<br><br>1. The extension and refinement of existing collaborative work. Given that much of the research undertaken will be novel, it is expected that the outcomes of some of the research will prompt additional lines of inquiry.<br><br>2. Requests for specific insight might emerge from the SeaBOS members (as a group, or prompted by an individual member or task force) or from the SRC Science Director.<br><br>3. Proposals might come from the SRC in response to a need to better understand the Keystone Dialogue process or from emerging questions in the regulatory or academic landscape.	Potential resources for work:<br><br>1. Existing SRC grant from the consortium of philanthropic bodies (there is an existing research programme specified in these grants).<br><br>2. External academic contributions 'in kind' from the existing partnerships which are funded by general university resources or from grants held outside of SRC (e.g. PhD studentships and post-doc posts at University of Birmingham and Burgos).<br><br>3. Contributions 'in kind' from SeaBOS members (e.g. vessel or personnel time in trialling programs).<br><br>4. Specific new grant funding (from academic or philanthropic sources) that has been obtained to support specific or general work on the Keystone Dialogues.	Issues that need to be agreed for work to be done in a timely fashion:<br><br>1. Both identify and specify the work to be conducted (including time lines and nature of work being undertaken).<br><br>2. Both identify how this work fits with the priorities of SeaBOS and the SRC.<br><br>3. SRC identify who from the SRC will lead the work, who will undertake the work, and who will provide quality control.<br><br>4. Both identify who from SeaBOS will be needed to collaborate in the work.<br><br>5. Both identify any external participants who should be involved in this work and the nature of those interactions.<br><br>6. SRC identify if proper ethical oversight/agreements for the work are in place.<br><br>7. Both identify the form in which work will be delivered (e.g. background briefs, working papers, academic/practice/policy articles etc).

2. *Science of SeaBOS*

This mode of research provides an understanding of the process and its outcomes. The development of a Keystone Dialogue process (which has resulted in the creation of SeaBOS) is innovative and unique.

As a result, the SRC (supported by the SeaBOS science funders) are monitoring how SeaBOS evolves over time. This involves understanding the internal dynamics of SeaBOS, the importance of existing networks and initiatives, and the role of partners, including individuals (e.g. The Hon. Jane Lubchenco Ph.D.) and organisations (eg High Level Panel for a Sustainable Ocean Economy, Global Dialogue on Seafood Traceability, the UN Global Compact on sustainable ocean business), as well as the critical support provided by Her Royal Highness Crown Princess Victoria of Sweden, a global SDG advocate Alumni).

The main audience for this information is the international scientific community, SeaBOS members, international policy makers, international seafood industry, philanthropic funders, environmental NGOs, and other stakeholders.

Science of SeaBOS will answer questions such as: "What has been achieved and how?". Science of SeaBOS will develop an understanding of *if and how* industry can accelerate change towards ocean stewardship, providing insights of what works well, and what does not work well. Scientific publications are the main venue for presenting results. This mode of research clarifies the need for, and potential in, transformative change, while also describing the Anthropocene reality and providing a systems perspective that clarifies the new reality for companies.

It is hoped that this science work will inform other attempts at Keystone Dialogues in different production sectors, as well as increase understanding of how corporations engage in transformations for stewardship.

Part of this work takes place naturally alongside the Science for SeaBOS, including participating in meetings and through working alongside SeaBOS staff. From time to time, however, it is necessary to more formally reflect upon the SeaBOS work and this might take the form of process documentation (e.g. collecting data on SeaBOS meetings), interviews and questionnaire surveys of SeaBOS participants, or studies of public SeaBOS members reports. Combined, these and other sources of information provide perspectives on the initiative, the processes it follows, and the results it generates.

3. *Principles of all research engagement*

1. Any data asked for will be either used as part of the direct scientific support for SeaBOS (Science for SeaBOS), or the science aimed at monitoring SeaBOS (Science of SeaBOS). The purpose of the data asked for will be made clear at the onset.

2. Information submitted will normally be anonymized and only used in an aggregated form. At times (for example, where shared learning is being sought) individual companies may be identified. In these instances, data will only be shared within SeaBOS. When this latter situation pertains, this will be made clear at the outset of any particular survey.

3. Participation in all targeted surveys is voluntary and consent to participate in such surveys is obtained (most usually by deciding to participate in the survey).

4. As scientists we follow best research practice by complying with existing laws and practice on confidentiality when collecting, analyzing, storing and publishing data.

5. All research will adhere to the requirements of anti-trust law. For example, research will not deal with or share any commercially sensitive information. Commercially sensitive information covers a wide range of information, and includes pricing or pricing strategies, costs, revenues, profits, margins, output, business or strategic plans, marketing, advertising, promotion plans, unannounced investments or developments plans, proposed R&D, marketing strategies, future market entry, price increases, discounts, tenders or whether to submit tenders, market shares and volumes or other aspects of competition.

6. Where agreement is obtained, aggregated results from research undertaken by the scientists will be made publicly available (for example as scientific background briefs on the web-page, in scientific publications, or other forms).

7. All data collected by the science team and any results it generates will be held by the science team.

8. The scientific engagement by scientists with SeaBOS as an organization or its members companies does not mean that these scientists, their institutions, or funders, necessarily agree with or endorse the activities of SeaBOS or its members.

…………………..

Supplementary Data S4: Secretariat and SRC Operational Principles.

SeaBOS Secretariat and SRC Operational Principles

Clarifying roles and responsibilities: These operational principles are developed between the SeaBOS secretariat and SRC in relation to the respective roles and responsibilities of the two organizations

Compiled by: [redacted]

Background

The Stockholm Resilience Centre (SRC) and the Royal Swedish Academy of Sciences (KVA) identified a number of Keystone Actors of the global seafood industry in 2015 (Österblom et al. 2015), described as having a disproportionate ability to influence change. These actors are defined as dominating revenues and production volumes, controlling major segment of production, connecting ecosystems through subsidiaries, and influencing governance processes and institutions. It was perceived that joint leadership in sustainability by such actors, could potentially generate cascading effects throughout the industry.

The SRC and KVA subsequently engaged with these keystone actors, including by organizing a series of Keystone Dialogues, which resulted in the establishment of the SeaBOS (Seafood Business for Ocean Stewardship). Scientists and business representatives have co-developed a shared vision and agenda, in a process between 2015-2019, including through bilateral dialogues (2015-2016). A series of Keystone Dialogues with the CEOs of the Keystone Actor companies has been organized (November 2016 in the Maldives, May 2017 in Stockholm, September 2018 in Karuizawa) – where commitments to engage in ocean stewardship, and associated activities were agreed on and developed. Two SeaBOS working meetings (May 2018 in Amersfoort, May 2019 in Bergen) have been organized to advance these commitments and exchange learning between operational staff and among SeaBOS members.

The SRC and the KVA have been responsible for facilitating and coordinating this process, with support from SeaBOS members. The SRC has operated as an interim SeaBOS secretariat between May 2017 and until now (May 2019). As the SeaBOS initiative is developing, an increased responsibility for funding and coordination will be transferred to the SeaBOS members and its secretariat. The aim is that SeaBOS matures into an independent, industry-led and mostly self-financing sustainable seafood and oceans organization that is measuring its members' commitments to sustainability and is growing its membership base during the coming years. Members of SeaBOS agree that this development requires support from independently funded research, conducted by the SRC and the KVA.

An agreement was reached between SeaBOS members in September 2018, that the companies will staff and fund an independent secretariat, registered in Sweden. SeaBOS members also appointed their first Chairman at this meeting, and early 2019 has been devoted to recruiting a Managing Director for the SeaBOS secretariat and to formalize the registration of the SeaBOS secretariat as a legal entity.

Purpose

This document specifies the roles and responsibilities of the SRC, the SeaBOS secretariat and the SeaBOS members. It represents a set of principles that applies in the relationship between the SRC and the SeaBOS secretariat. This document and the outlined roles and responsibilities (Table 1) should be regarded as general, whereas further details will be regulated in separate documents, and formalized agreements as necessary. It should also be noted that the roles and responsibilities will shift over time (Figure 1), towards increased responsibility of the SeaBOS secretariat to manage coordination, along with internal and external communication. The SRC represents a key scientific partner to SeaBOS, but has no formal responsibility for the performance of SeaBOS.

Science

The SeaBOS initiative is a result of the Keystone Dialogues. The Keystone Dialogues is a scientific approach to investigate the potential of keystone actors to stimulate transformative change. The SeaBOS secretariat will support this effort. SeaBOS members will continuously engage with science, including through dialogue and cooperation that advances the scientific endeavor to study the role of keystone actors in the seafood production system (e.g. through interviews and surveys). The specific commitments by companies to engage in science, including rules associated with anonymity and ethics, will be developed in a separate agreement.

Funding

The SRC and the SeaBOS secretariat will operate with individual and independent budgets.

Duration

This document is open-ended, and either party can terminate this cooperation if the legitimacy of SeaBOS is perceived to have been compromised .

Table 1. The roles and responsibilities of SRC, the SeaBOS secretariat and SeaBOS members.

	**Activity categories**					
	**Task forces**	**Meetings***		**External communicatio n**	**Monitoring impact of SeaBOS initiative**	**Science and learning**
		Keystone Dialogues (CEO-level)	Working meetings (operational level)			
**Stockholm Resilience Centre (SRC)**	Scientific and strategic support	Scientific support and agenda development. Scientific leadership participation in meetings.	Scientific support and agenda development. All relevant scientists participation in meetings.	Communicatio n at scientific conferences, trade shows, UN conferences. Communicatio n with partners (emphasis on scientific community, NGOs, governments)	SRC leads monitoring efforts. Engages in co-design with SeaBOS members and secretariat	SRC leads all scientific activities. Engages in co-design of scientific program with SeaBOS Secretaria t and members based on identified needs.
**SeaBOS Secretaria t**	Strategy, coordination, management, internal communication s	Lead for coordination, logistics, content and execution of meetings.	Lead for coordination, logistics, content and execution of meetings.	Communicatio n at trade shows and UN conferences (in collaboration with SRC). Communicatio n with partners and broader landscape (emphasis on business and governments).	Co-design monitoring strategy with SRC	Co-design scientific program based on identified science needs
**SeaBOS members**	Active leadership and management (at the level of operational change)	CEO-level participation	Operational staff participation	Participation as appropriate	Active and regular contributio n of data for monitoring	Co-design scientific program based on identified science needs

** Note: Meeting participation costs covered by SRC (for SRC reps) by SeaBOS Secretariat (for Secretariat reps) and by member companies (for their reps)*

Figure 1. A time plan for the transition towards independence.

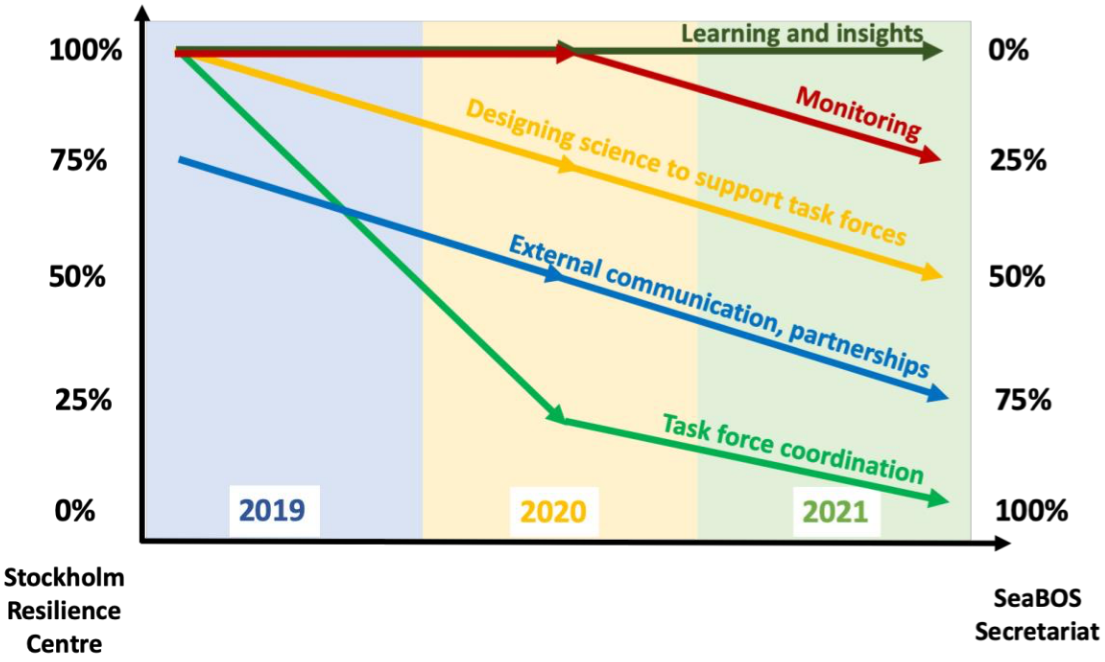

Supplementary References

1 Österblom, H. *et al.* Transnational Corporations as 'Keystone Actors' in Marine Ecosystems. *PLOS ONE* **10**, e0127533, doi:10.1371/journal.pone.0127533 (2015).

2 Geerts, B., Beukers, R. & Nakamura, K. Seafood Stewardship Index methodology. (World Benchmarking Alliance, Amsterdam, 2019).

3 Nissui. Sustainability Report 2019. (Nippon Suisan Kaisha, Ltd., Tokyo, Japan, 2019).

4 MNC. *マルハニチロク ゙ルーフ ゚取扱水産物の資源調査結果*, <https://www.maruha-nichiro.co.jp/corporate/sustainability/environment/maintenance/pdf/resource_survey_results.pdf> (2021).

5 Solberg, E. & Ito, S. in *Financial Times* Vol. June 27, 2019 (2019).

6 Cargill, Skretting, BioMar & Mowi. Fish feed majors call for scientific limits for fishing quotas. *Salmon Business* (2020).

7 SeaBOS. *SeaBOS calls on governments to support boat crews and ocean workers*, <https://seabos.org/news/seabos-calls-on-governments-to-support-boat-crews-and-ocean-workers/> (2020).

8 Pickerell, T., Jackson, S., Bergjord, T. L., Schorr, D. & Wisse, H. *Statement on Traceability and Port State Measures*, <https://seabos.org/wp-content/uploads/2021/02/Coalition_Statement_traceability-and-port-state-measures.pdf> (2021).

9 GTA, ISSF & SeaBOS. *Global seafood coalition applauds Japanese government on actions to eliminate IUU fishing; calls for further collaboration between industry and governments*, <https://seabos.org/news/global-seafood-coalition-applauds-japanese-government-on-actions-to-eliminate-iuu-fishing-calls-for-further-collaboration-between-industry-and-governments/> (2021).

10 EDF & SeaBOS. *Climate Resilience and Equity Highlighted in COFI Declaration - Vision for 21st century includes recognition of need to address climate impacts*, <https://www.edf.org/media/climate-resilience-and-equity-highlighted-cofi-declaration> (2021).

11 Anonymous. (Undercurrent News, https://www.undercurrentnews.com/2019/10/25/group-of-worlds-largest-seafood-firms-join-ghost-gear-initiative/, 2019).
